# A Genome-Wide Association Study of Resistance to Stripe Rust (*Puccinia striiformis* f. sp. *tritici*) in a Worldwide Collection of Hexaploid Spring Wheat (*Triticum aestivum* L.)

**DOI:** 10.1534/g3.114.014563

**Published:** 2015-01-20

**Authors:** Marco Maccaferri, Junli Zhang, Peter Bulli, Zewdie Abate, Shiaoman Chao, Dario Cantu, Eligio Bossolini, Xianming Chen, Michael Pumphrey, Jorge Dubcovsky

**Affiliations:** * Department of Plant Sciences, University of California, Davis, California 95616; † Department of Agricultural Sciences (DipSA), University of Bologna, Bologna 40127, Italy; ‡ Department of Crop and Soil Sciences, Washington State University, Pullman, Washington 99164-6420; § USDA-ARS, 1605 Albrecht Blvd, Fargo, North Dakota 58105; ** Department of Viticulture and Enology, University of California, Davis, California 95616; †† USDA-ARS, Wheat Genetics, Quality Physiology, and Disease Research Unit, and Department of Plant Pathology, Washington State University, Pullman, Washington 99164; ‡‡ Howard Hughes Medical Institute, Chevy Chase, Maryland 20815

**Keywords:** disease resistance, genetic map, yellow rust, bread wheat, association mapping

## Abstract

New races of *Puccinia striiformis* f. sp. *tritici* (*Pst*), the causal pathogen of wheat stripe rust, show high virulence to previously deployed resistance genes and are responsible for large yield losses worldwide. To identify new sources of resistance we performed a genome-wide association study (GWAS) using a worldwide collection of 1000 spring wheat accessions. Adult plants were evaluated under field conditions in six environments in the western United States, and seedlings were tested with four *Pst* races. A single-nucleotide polymorphism (SNP) Infinium 9K-assay provided 4585 SNPs suitable for GWAS. High correlations among environments and high heritabilities were observed for stripe rust infection type and severity. Greater levels of *Pst* resistance were observed in a subpopulation from Southern Asia than in other groups. GWAS identified 97 loci that were significant for at least three environments, including 10 with an experiment-wise adjusted Bonferroni probability < 0.10. These 10 quantitative trait loci (QTL) explained 15% of the phenotypic variation in infection type, a percentage that increased to 45% when all QTL were considered. Three of these 10 QTL were mapped far from previously identified *Pst* resistance genes and QTL, and likely represent new resistance loci. The other seven QTL mapped close to known resistance genes and allelism tests will be required to test their relationships. In summary, this study provides an integrated view of stripe rust resistance resources in spring wheat and identifies new resistance loci that will be useful to diversify the current set of resistance genes deployed to control this devastating disease.

Stripe rust disease of wheat, caused by the fungus *Puccinia striiformis* Westend. f. sp. *tritici* Erikss. (henceforth *Pst*), poses a major threat to global wheat production (Chen 2005; Milus *et al.* 2009) and negatively affects grain quality (Dimmock and Gooding 2002). Although multiple fungicide applications can control this pathogen, developing resistant varieties is the most efficient and environmentally sustainable means for reducing losses due to this disease (Line 2002; Chen 2005).

*Pst* accessions are classified into physiological races based on their virulence profile on a set of wheat differential lines (Wan and Chen 2014) (*e.g.*, Supporting Information, Table S1), whereas *Pst* resistance genes are classified as either race-specific or race nonspecific based on their effectiveness against different *Pst* races. Race-specific resistance genes, which are effective only against a subset of races, usually are expressed from the seedling to the adult plant stages and generally result in a strong hypersensitive response associated with high levels of resistance. Race nonspecific resistance genes are effective against all *Pst* races, generally are expressed at adult plant stages, and are characterized by various degrees of resistance (quantitative or partial resistance). Many of these partial resistance genes exhibit enhanced resistance at high temperatures and are designated as high-temperature adult plant resistance genes (Chen 2013). Pyramiding multiple partial resistance genes is required to confer adequate levels of resistance (Singh *et al.* 2000; Singh *et al.* 2005). Both race-specific and race nonspecific resistance or partial resistance genes have been used in breeding for rust resistance (Chen 2005; Lowe *et al.* 2011a). However, high genetic variation in the pathogen population and the rapid rate of selection for new virulent races have forced wheat breeders to focus on pyramiding strategies that combine multiple race-specific and/or race nonspecific resistance genes to increase the durability of the deployed resistance.

The 2013 Catalogue of Gene Symbols for Wheat (McIntosh *et al.* 2013) and the 2013−2014 Supplement (http://wheat.pw.usda.gov/GG2/Triticum/wgc/2013/2013-2014_Supplement.pdf) include 67 officially named *Yr* genes (*Yr1*−*Yr67)* and 42 with temporary *Yr* designations. At least 16 of the named genes have been introduced into wheat varieties and breeding lines from wheat relatives and alien species (Chen *et al.* 2014). Unfortunately, most of these resistance genes are no longer effective against a new group of *Pst* races that appeared around the year 2000 (Chen *et al.* 2010), which has generated the need for the identification of new sources of resistance. Although recent quantitative trait loci (QTL) studies have documented additional sources of resistance (Lowe *et al.* 2011b; Sukhwinder-Singh *et al.* 2012; Vazquez *et al.* 2012; Christopher *et al.* 2013), the use of biparental populations limits the extent of the germplasm that can be explored for new sources of resistance.

Genome-wide association studies (GWAS) provide comprehensive surveys of germplasm collections and are an excellent complement to biparental mapping studies. Because GWAS exploits historical recombination events accumulated over multiple generations, the short evolutionary history of polyploid wheat (Dubcovsky and Dvorak 2007) and its self-pollinating reproductive system result in lower mapping resolution compared with outcrossing species with a longer evolutionary history such as maize (Chao *et al.* 2010). An advantage of the high levels of linkage disequilibrium (LD) reported in wheat is that the number of markers required for finding marker-trait associations is greatly reduced (Chao *et al.* 2010).

To avoid the discovery of false marker-trait associations, GWAS studies require adequate assessment and correction for population structure. (Flint-Garcia *et al.* 2003; Yu *et al.* 2006; Kang *et al.* 2008; Stich *et al.* 2008; Larsson *et al.* 2013). Even with this correction, GWAS has limited power to detect allele variants present in rare frequencies (Brachi *et al.* 2011; Wallace *et al.* 2014; Zuk *et al.* 2014) or loci with multiple allelic variants (Zhang *et al.* 2012).

Despite these limitations, GWAS has been used successfully in mapping QTL for different traits in several plant species (Brachi *et al.* 2010; Zhao *et al.* 2011; Wang *et al.* 2012; Jia *et al.* 2013; Lipka *et al.* 2013). In wheat, GWAS has been used successfully to study agronomic traits (Breseghello and Sorrells 2006; Yao *et al.* 2009; Dodig *et al.* 2012), quality traits (Reimer *et al.* 2008; Reif *et al.* 2011), preharvest sprouting (Mohan *et al.* 2009; Kulwal *et al.* 2012), and disease resistance (Adhikari *et al.* 2011; Hao *et al.* 2012; Kollers *et al.* 2013; Letta *et al.* 2013).

In this study, we evaluated 1000 accessions from the US Department of Agriculture Agricultural Research Service (USDA-ARS) National Small Grains Collection (NSGC) spring wheat core collection for resistance against *Pst*. Resistance was evaluated both at the seedling stage in controlled environments (to four specific *Pst* races) and at the adult plant stage in multiple years and field locations in the western United States (to mixtures of naturally occurring *Pst* races). We found evidence for 10 high-confidence associations that were consistent across locations and compared their chromosome locations with previously mapped *Pst* resistance genes and QTL. We also identified the colinear regions in the rice and *Brachypodium* genomes to accelerate the identification of additional markers for these QTL regions.

## Materials and Methods

### Plant materials

One thousand accessions were selected randomly from the 2235 *Triticum aestivum* ssp. *aestivum* accessions available in the spring wheat core collection assembled by the USDA-ARS Small Grains and Potato Germplasm Research Unit. This core collection was assembled using passport and phenotypic data and includes accessions from the different wheat producing regions of the world. Accessions from 89 countries were represented in this study, including accessions from South America (20.9%), Africa (20.6%), Europe (19.6%), Asia (29.1%), North America (7.0%), and Australia (2.8%). Seeds and DNAs used in this study were obtained from single plant selections increased in a nursery grown at the USDA-ARS Small Grains and Potato Germplasm Research Unit, Aberdeen, Idaho. The genetic characterization of these 1000 accessions [see *SNP genotyping* of *Material and Methods* below] resulted in the selection of 875 nonredundant accessions for the GWAS analysis, which are listed in File S1.

### Stripe rust response evaluation: adult-plant field conditions

Accessions were evaluated under natural disease epidemics in six field trials performed at three locations: Mount Vernon (48° 25′ 12′′N 122° 19′ 34′′W) in the western side of Washington state, Pullman (46° 43’ 59′′ N 117° 10′ 00′′W) in the eastern side of Washington state, and Davis (38° 33′ 14′′N 121° 44′ 17′′W) in northern California. Trials in Davis and Pullman were performed in 2011 and 2012, and those in Mount Vernon in 2012 and 2013. The different year-location combinations are referred hereafter as “environments.” Planting was in mid-November in Davis and mid-April for both Mount Vernon and Pullman. Oversummering and overwintering of stripe rust can occur in both Pacific Northwest and California, facilitating local recurrent epidemics (Sharma-Poudyal *et al.* 2013). *Pst* inoculum often is shared between California, Arizona, New Mexico, and Northwestern Mexico. Additionally, *Pst* oversummering in northeastern California may constitute a source of inoculum for central California (Kolmer *et al.* 2009) and for the Pacific Northwest (Chen 2005).

In all field trials, accessions were evaluated as nonreplicated single rows. Rows were 2.0 m long with 0.40 m spacing between rows in experiments at Davis, and 1.0 m long with 0.25 m spacing at both Mount Vernon and Pullman. The susceptible checks used were “D6301,” planted every six rows in Davis, and “Lemhi,” planted every 20 rows in Washington. The same susceptible checks also were planted as spreader rows bordering the nurseries to ensure production of sufficient inoculum to provide uniform stripe rust infection.

Stripe rust response was evaluated twice during the mid- to advanced-phases of disease development to limit the number of escapes. These evaluations were performed between plant heading (Zadoks 50) and grain filling stage (Zadoks 80), when most flag leaves of the susceptible checks displayed a disease severity of at least 50%. Only the evaluation showing the highest average disease pressure between the two stages (usually the second one) was used in the GWAS analysis. The infection type (IT) was scored using a 0−9 scale described previously (Line and Qayoum 1992). Disease severity (SEV) was scored as percentage of infected leaf area. Additionally, days to heading and plant height were evaluated to test their correlation with *Pst* resistance. Other morphophysiological traits such as occurrence of pseudo-black chaff, awns, wax, and glume hairiness were recorded as controls for line identification.

### Stripe rust response evaluation: single-race seedling test

Seedlings of the 875 accessions were evaluated for IT response to four *Pst* races, PSTv-4, PSTv-14, PSTv-37, and PSTv-40 (Wan and Chen 2014), under controlled greenhouse conditions. The *Pst* races were maintained at the USDA-ARS, Washington State University. PSTv-37 is a predominant and widely distributed race in the United States, whereas PSTv-4, PSTv-14, and PSTv-40 are predominantly found in the Pacific Northwest and California. The virulence/avirulence formulas of the four races are described in Table S1 (Wan and Chen 2014).

Three seeds of each accession were planted per well in a 96-well tray containing Sunshine mix growing medium (SunGro Horticulture, Agawam, MA). Lines of the stripe rust differential set and the stripe rust-susceptible “Avocet S” were seeded in a separate 96-well tray. The seeded trays were placed in a rust-free greenhouse at 20° with 50% relative humidity. Seedlings were watered daily, and the gibberellin inhibitor (2-chloroethyl trimethylammonium chloride; Ohp, Inc, PA) was used at a concentration of 1500 ppm to slow down the seedling growth rate. Trays of 11-d-old seedlings were inoculated with urediniospores of each race mixed with talc. The inoculated seedling trays were placed in a dark dew chamber overnight at 10° with 100% relative humidity for 20 hr. After the incubation, seedlings were transferred to a greenhouse with a diurnal temperature cycle programmed to change gradually from 20° at 2:00 pm to 4° at 2:00 am. Day/night regimes of 16-hr light and 8-hr darkness were maintained throughout the experiment. ITs were scored 18−20 d after inoculation when the rust was developed fully on the susceptible checks. ITs were scored using a 0−9 scale (McNeal *et al.* 1971) similar to that used for the adult plants under field conditions. Accessions with resistant to moderately resistant IT scores of 0−6 were retested with each respective *Pst* race.

### SNP genotyping

For each accession, genomic DNA was extracted from the same plant used to increase the seeds evaluated in this study. DNA extractions were performed at the USDA-ARS Small Grains and Potato Germplasm Research Unit using the CTAB protocol (Stewart and Via 1993). The DNA was precipitated by adding isopropanol, followed by washing of the pellet with ice-cold 70% ethanol, and resuspension in 200 µL of Tris HCl ethylenediaminetetraacetic acid (pH 8.0).

Genotyping was carried out at the USDA-ARS genotyping laboratory at Fargo, North Dakota, using the Infinium wheat SNP 9K iSelect assay from the Illumina platform (Illumina Inc., San Diego, CA) developed by the International Wheat SNP Consortium (Cavanagh *et al.* 2013). The raw Illumina SNP data were processed with the GenomeStudio v2011.1 software (Illumina). The array yielded 5234 scorable SNP markers. The polymorphic SNPs were ordered according to the scaled map positions of the hexaploid wheat 9K SNP consensus map (Cavanagh *et al.* 2013). The arm orientation of chromosomes 4A, 5A, and 5B presented here is in opposite orientation to the published consensus map (Cavanagh *et al.* 2013).

The dataset was filtered using a 10% cutoff for missing data in either loci or accessions (23 accessions were eliminated). On the basis of the filtered SNP data, a triangular identity-by-state genetic similarity matrix (Kang *et al.* 2008) was then obtained for all possible pairs of accessions. For groups of accessions with ≥0.99 genetic similarity, only one representative accession (the one with the lowest number of missing data) was retained per group. After applying these filtering criteria, a total of 875 accessions were retained for the GWAS. Only SNPs with minor allele frequency (MAF) ≥0.10 (*i.e.*, minor allele present in at least 87 accessions) were considered for GWAS. Of the 4585 SNPs that satisfied this criterion, 4374 were positioned on the consensus map. Low-frequency SNPs were discarded to focus on SNPs with greater statistical power (Turner *et al.* 2011). The downside of this approach is the potential loss of true resistance loci present at low frequency (increase in false negatives). In this study we prioritized the reliability of the detected QTL over the sensitivity of the analyses.

Molecular markers tightly associated to two well-characterized loci conferring resistance to multiple pathogens were included as internal controls. The diagnostic *KaspLr34* assay (http://www.cerealsdb.uk.net/cerealgenomics/CerealsDB/SNPs/Documents/MAS, = wMAS000003) was designed around a 3-bp indel in exon 11 of the *Lr34/Yr18* gene (Lagudah *et al.* 2009). Marker *csSNP856* (=*Kasp856*) is tightly linked to the *Lr67/Yr46* locus but is not diagnostic for the resistance gene (Forrest *et al.* 2014). Data for these two control markers are summarized in File S3.

### Population structure and genetic diversity

The mapped SNP data, together with the map information from Cavanagh *et al.* (2013), were analyzed with HAPLOVIEW v4.2 (Barrett *et al.* 2005) using the tagger function *r*^2^ = 1.0 to define a set of 3114 nonredundant SNPs for calculation of the kinship matrix. A subset of 1036 highly informative, nonredundant representative SNPs (tagSNPs) also were selected with HAPLOVIEW using the tagger function *r*^2^ = 0.25. The tagSNPs were used for population structure analysis using a combination of distance- and model-based clustering analysis. Distance-based cluster analysis was carried out using the Ward clustering algorithm in R (R Core Team 2013) as implemented in “stats” package (hclust). The model-based quantitative assessment of subpopulation memberships of the accessions was carried out in STRUCTURE v 2.3.4 (Pritchard *et al.* 2000) using inferences based on molecular data only and admixture model of population structure with correlated allele frequencies. Numbers of hypothetical subpopulations ranging from *k =*1 to 10 were assessed using 50,000 burn-in iterations followed by 100,000 recorded Markov-Chain iterations. To estimate the sampling variance (robustness) of population structure inference, five independent runs were carried out for each *k*. The output from STRUCTURE was analyzed in STRUCTURE HARVESTER (Earl and Vonholdt 2012).

The *Δk* statistics based on the rate of change in the logarithm of the probability of likelihood [*LnP(D)*] value between successive *k* values (Evanno *et al.* 2005) was used to predict the optimum number of subpopulations. On the basis of the final *k* values (four groups and seven subgroups, respectively), two Q-matrices (875 × k) including the corresponding population membership coefficients were obtained.

To determine the level of differentiation among subpopulations, we calculated the fixation index (*Fst*) among all possible pairwise combinations of the seven subpopulations using the software Arlequin v. 3.5 (Excoffier *et al.* 2005). For this calculation we used all 4585 SNPs and used 1000 permutations at *P =* 0.001. We also calculated the gene diversity (D) of each of the 7 subpopulations using the 4585 SNPs with published formulas (Weir 1996).

### LD analysis

HAPLOVIEW was used to obtain the LD squared allele frequency correlation (*r*^2^) estimates for all pairwise comparisons between intrachromosomal SNPs and to visualize the local LD patterns. To analyze the overall pattern of LD decay over genetic distances, syntenic pairwise LD *r*^2^ estimates from all chromosomes were plotted *vs.* the corresponding pairwise genetic distances and a nonlinear regression model was fitted in R based on the equation relating LD, recombination rate, and population size (Sved 1971; Rexroad and Vallejo 2009). The curve fitting parameter *α* was set to 1 (no mutations). Nonlinear fitting of the model was carried out using the nonlinear least squares method in R. The map distance at which LD fell below the *r*^2^ thresholds of 0.3 was used to define the confidence intervals of QTL detected in this study. This is a frequently used LD threshold for QTL detection (Ardlie *et al.* 2002; Shifman *et al.* 2003; Khatkar *et al.* 2008; Lawrence *et al.* 2009).

### Phenotypic data analysis

To improve normality of the original phenotypic data, logit, square root, arcsine, and log transformation methods were tested. For IT, the data were square root transformed [IT_transf_ = √ (IT + 0.25)/10)] and for SEV the data were arcsine-square root transformed [SEV_transf_ = arcsine (√ 1-(SEV+0.25)/100)]. The Shapiro-Wilk normality test confirmed the improved normality of the transformed data relative to the original data. Even though the correlations between normal scores and best linear unbiased estimates (BLUE) values from all six locations (BLUE-all) calculated from the transformed data were relatively high (IT W = 0.98 and SEV W = 0.99), departures from normality were still significant.

The transformed data were subjected to combined analyses of variance (ANOVA) over environments using a mixed linear model procedure including genotype, environment and genotype by environment interactions as random factors. Broad sense heritability (*H^2^*) estimates (Table 1) were calculated using the Restricted Maximum Likelihood method (Corbeil and Searle 1976). BLUE values were obtained across locations and years considering genotypes as a fixed effect in the model. BLUE values were then used to perform GWAS.

**Table 1:** Means and ranges for response to *Puccinia striiformis* f. sp. *tritici* of 875 spring wheat accessions from the NSGC in six environments (three locations × two years)

	Mount Vernon (WA)		Pullman (WA)		Davis (CA)		Across Environments	
	IT	SEV	IT	SEV	IT	SEV	IT	SEV
Mean	4.5	51.8	4.1	50.7	3.9	48.3	4.1	50.3
Min.	0.0	0.0	0.0	0.0	0.0	0.0	0.0	0.0
Max.	9.0	100	9.0	100	9.0	100	9.0	100
$\sigma_{G}^{2}$	0.0241****	0.1199****	0.0208****	0.0759****	0.0231****	0.1201****	0.0207****	0.0951****
$\sigma_{E}^{2}$	0.0003^ns^	0.0112^ns^	0.0077^ns^	0.0302^ns^	0.0019^ns^	0.0088^ns^	0.0025^ns^	0.0100^ns^
$\sigma_{GE}^{2}$	0.00	0.00	0.00	0.00	0.0066**	0.0295*	0.00	0.0004^ns^
$\sigma_{e}^{2}$	0.0091**	0.0248****	0.0086****	0.0287****	0.0066**	0.0328*	0.0123***	0.0486****
$H^{2}$	0.84	0.87	0.72	0.72	0.75	0.77	0.89	0.91

Covariance estimates from the random model were calculated using the restricted maximum likelihood method on the transformed data. NSGC, National Small Grains Collection; IT, infection type; SEV, disease severity;$\sigma_{G}^{2}$, genotype variance estimate; $\sigma_{E}^{2}$, environment variance estimate; $\sigma_{GE}^{2}$, genotype × environment variance estimate; $\sigma_{e}^{2}$, residual variance estimate; $H^{2}$, broad sense heritability; ns, not significant.

* *P* < 0.05; **P < 0.01; ***P < 0.001; ****P < 0.0001.

Pearson correlation coefficients among environments were calculated for IT and SEV values to evaluate the consistency of the resistance responses. Correlations between field-based *Pst* resistance responses and both heading date and plant height were calculated to investigate the influence of these traits on resistance. The proportion of variation (*R^2^*) in *Pst* response across accessions explained by population structure was calculated using multiple regression of single environment- and adjusted mean-phenotypes on the quantitative Q-7 STRUCTURE membership coefficient matrix.

### Association analysis

GWAS for loci governing *Pst* response in the filtered set of 875 accessions was performed using 4585 informative SNPs and the compressed mixed linear model (Yu *et al.* 2006; Zhang *et al.* 2010) implemented in the R package GAPIT (Lipka *et al.* 2012). Association tests were carried out for: (1) all single environment data sets, (2) BLUEs across experiments (years) for each location, and (3) BLUEs across all six environments.

Different association test models were compared using the Bayesian information criterion calculated using GAPIT for IT and SEV data from all environments (Table S2). The following models were tested: (1) fixed general linear model with no correction for population structure, (2) general linear model models corrected for population structure using the first 10 eigenvectors from principal component analysis (PC10), percent membership coefficients based on STRUCTURE (Q4 and Q7), and qualitative assignment to Ward clusters (W4 and W7, where 4 indicates the four main groups and seven the subpopulations), (3) mixed linear model with the 875 × 875 kinship matrix (K), and (4) all possible combinations of K and the other five methods. Models were also evaluated using plots of observed *vs.* expected cumulative *P* values based on the IT data from Davis 2011 (Stich *et al.* 2008) (Figure S1). All mixed linear models including K performed similar to each other and better than models without K (Table S2 and Figure S1). In addition, the K mixed linear model without additional corrections showed the best Bayesian information criterion value for both IT and SEV across all environments (Table S2) and was selected for all GWAS analyses. Association probability values were estimated and both marker-wise and experiment-wise thresholds were selected for different analyses. The selected experiment-wise threshold was based on the Bonferroni-corrected method with *α* = 0.10, which is roughly equivalent to a marker-wise threshold *P* value = 1 × 10^−4^ (9.65 × 10^−5^ for 1036 tag-SNPs).

QTL that were significant (*P* ≤ 0.05, marker-wise) in at least three of the six environments for IT and/or SEV, with at least one environment with highly-significant differences (*P* ≤ 0.01, marker-wise) were first selected to identify *Pst* resistance loci with broad-spectrum resistance effective across multiple locations. The same QTL selection criteria were applied to a second GWAS that included only accessions with BLUE across all locations (BLUE-all) IT scores greater than 3. The objective of this second analysis, which included 593 accessions, was to find QTL for partial resistance that might have been masked by the presence of major resistance genes. The 97 QTL that passed the previous two selection criteria for IT or SEV are summarized in the Supplemental Information, Table S4, Table S5, Figure S2, and Figure S3. Among these 97 QTL, only 10 were significant for the Bonferroni test (experiment-wise *P* ≤ 0.10) and are discussed in detail in this study. The cumulative effect of the 10 significant QTL and their interactions was estimated using an ANOVA model including population structure (Q7) as covariate.

Multiple colocating and/or adjacent SNPs in the consensus map significantly associated with the disease response were assigned to a single QTL when the LD *r*^2^ values among markers were ≥ 0.3, inter-marker distances were within the QTL confidence interval, and the SNPs showed consistent direction of the effects. The SNPs with *P* values indicating the strongest association was considered as the QTL-representative marker (henceforth, QTL-tagging SNP).

### Enrichment of markers in the QTL regions and preliminary annotation

To increase the number of markers in each QTL region, the consensus map from the Illumina 90K SNP assay (Wang *et al.* 2014) was projected onto the 9K SNP consensus map (Cavanagh *et al.* 2013), which was used as reference map in BIOMERCATOR v4.2 (Sosnowski *et al.* 2012). Flanking sequences of all the 9K and 90K Illumina SNPs that mapped within the QTL confidence intervals (± 1.6 cM) were then used to BLAST *T. turgidum* (Kronos) transcriptome, *T. urartu* transcriptome (Krasileva *et al.* 2013), and *A. tauschii* cDNA database (http://plants.ensembl.org/Aegilops_tauschii/Info/Index) to obtain longer query sequences for colinearity analysis. Sequence hits with alignment length more than 99% of the query and more than 98% identity were used as query in BLASTX searches of *Brachypodium* (*Brachypodium distachyon* (L.) P. Beauv) and rice (*Oryza sativa* L.) databases using Gramene (Monaco *et al.* 2014) (http://www.gramene.org). Annotations for the orthologous *Brachypodium* and rice genes were retrieved from Phytozome v9.1 (http://www.phytozome.net/). Results from protein domain databases (pFAM domain descriptions), Protein ANalysis THrough Evolutionary Relationships, EuKaryotic Orthologous Groups description, and Gene Ontology terms are summarized in File S2. Based on these annotations, we identified *Brachypodium* and rice *R* genes, which encode proteins that recognize pathogen effectors or their modified host targets. These proteins are characterized by the presence of a variable leucine-rich repeat (LRR) and include CC-NB-LRR (coiled-coil domain, nucleotide binding site, LRR), RLP (receptor-like proteins coupled with extracellular LRR), and RLK (kinase domain coupled with LRR) (Chisholm *et al.* 2006).

### Comparison of QTL locations with previously reported *Yr* genes and QTL

For comparison with previous studies, the 10 experiment-wise significant QTL identified in the GWAS, 56 named *Yr* genes (McIntosh *et al.* 2013) (catalogue of gene symbols for wheat: 2013−2014 Supplement; http://wheat.pw.usda.gov/GG2/Triticum/wgc/2013/2013-2014_Supplement.pdf) and 169 previously mapped QTL were projected onto a common integrated map including different marker types (File S4).

The integrated map was produced extending the iterative map compilation process described in the previous section (“iterative maps compilation” tool in Biomercator v4.2 (Sosnowski *et al.* 2012)). The process started with the 9K SNP consensus map (Cavanagh *et al.* 2013), followed by the sequential projection of the 90K SNP consensus map (Wang *et al.* 2014), the tetraploid consensus map (Maccaferri *et al.* 2014), the Synthetic × Opata DH GBS map (Saintenac *et al.* 2013), the Diversity Array Technology integrated map (http://www.diversityarrays.com/search/node/Wheat%20DArT%20map), the 2004 SSR consensus map (Somers *et al.* 2004), and the Synthetic × Opata ITMI BARC SSR map (Song *et al.* 2005). The software option for automatic resolution of common markers inversions was used.

For the named *Pst* resistance genes the closest flanking markers were used to generate confidence intervals that are reported in File S5. Distances in cM were converted into relative % length distances by dividing them by total chromosome length, and were then projected onto schematic chromosomes. Confidence intervals for published QTL were calculated using Darvasi and Soller prediction formulas (Darvasi and Soller 1997). QTL identified in this GWAS were projected on the same compiled map, using confidence intervals of ± 1.6 cM (where LD was predicted to fall below the critical levels of *r*^2^ = 0.3).

## Results

### Spring wheat panel composition and population genetic structure

**Figure 1:**
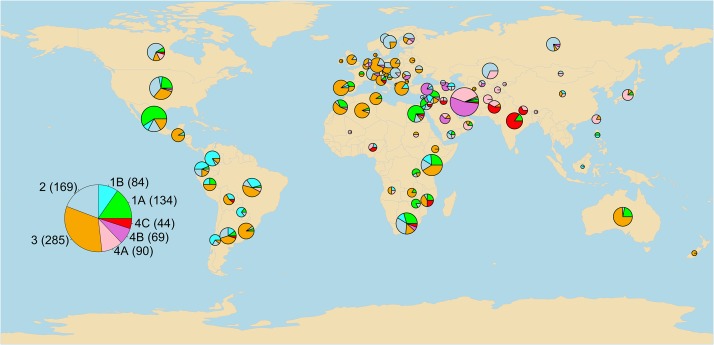
Geographic distribution of seven subpopulations identified in the analysis of the population structure of 875 accessions from the National Small Grains Collection spring wheat core collection. The large pie chart indicates the relative number of accessions in the seven subpopulations. The smaller pie charts indicate their relative distribution in specific countries (the size of these pie charts is proportional to the number of accessions from that country).

**Figure 2:**
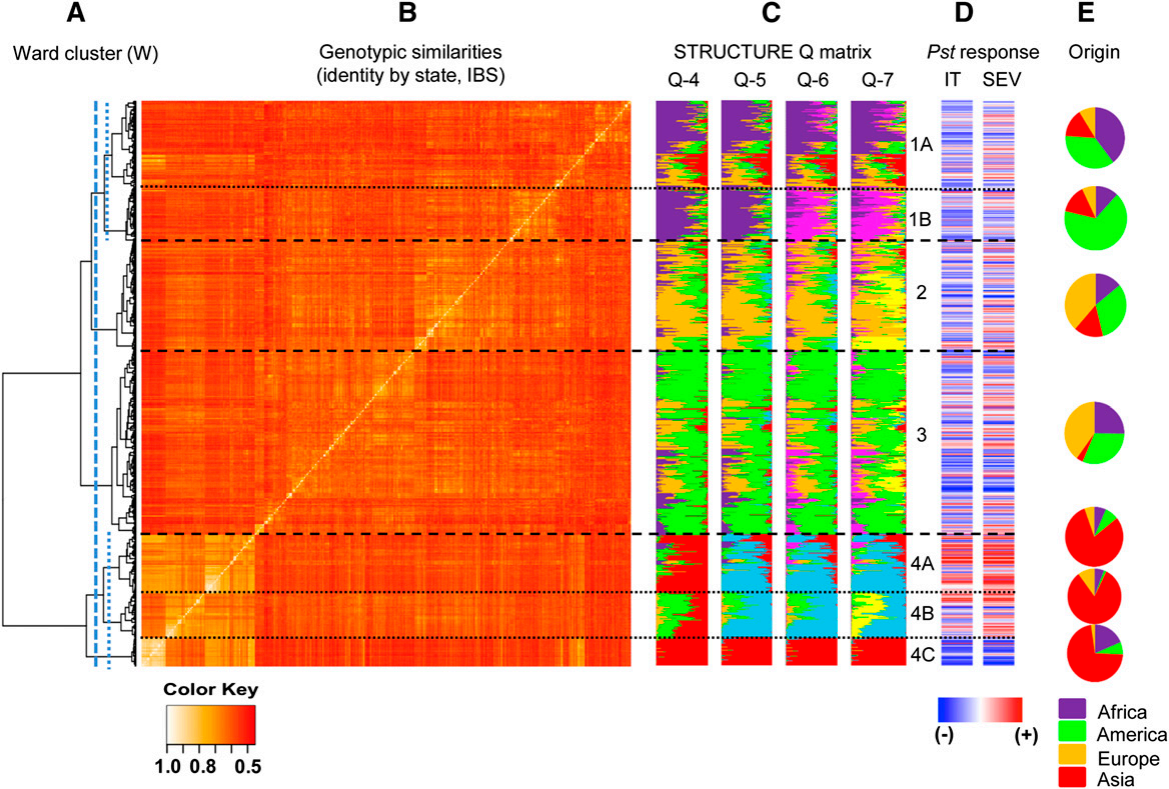
Population structure and its relationship to *Puccinia striiformis* f. sp. *tritici* (*Pst*) resistance. (A) Ward’s clustering of 875 accessions from the NSGC spring wheat core collection. Vertical lines indicate genetic similarity thresholds used to classify accessions into 4 main groups (dashed lines) and 7 subgroups (dotted lines). (B) 875 × 875 kinship matrix based on simple matching genetic similarities (IBS, identity by state). Separations among Ward’s based groups are shown as horizontal dashed lines for main groups and as dotted-dashed lines for subgroups. (C) Matrices of membership coefficients of accessions corresponding to 4−7 hypothetical subpopulations derived from the STRUCTURE analysis (D) Response of accessions to *Pst* (IT, infection types, SEV, disease severity). Blue lines indicate *Pst* resistance and red lines *Pst* susceptibility (based on the best linear unbiased estimates over six environments). (E) Percentage memberships of accessions from the seven subpopulations to the four main continents.

Based on genetic profiles of the Illumina iSelect 9K SNP array, 875 nonredundant genotypes with less than 10% missing data were identified among the 1000 spring *T. aestivum* ssp. *aestivum* accessions initially selected from the NSGC core wheat collection. These 875 accessions (File S1) included 172 landraces, 255 registered cultivars, 299 breeding lines, 6 genetic stocks, and 143 unclassified accessions. The accessions originated from 87 countries on six continents and are representative of the diverse wheat growing areas in the world (Figure 1). The characterization of these 875 accessions using a set of nonredundant genome-wide tagSNPs and a combination of hierarchical Ward clustering and quantitative population structure model-based Bayesian clustering revealed the presence of four main groups. Groups 1 and 4 were further subdivided into two and three subgroups, respectively, giving a total of seven subgroups that reflect the population structure of these accessions (Figure 2).

This population structure showed some association with the geographic distribution of the accessions present in this panel (Figure 1). Group 1A was well represented in Mexico, in the Middle East (Egypt, Israel, and Syria), and in the eastern and southern regions of Africa (Kenya, Zimbabwe, and South Africa). Group 2 was predominant in accessions from Russia, Kazakhstan, Northern, and Eastern Europe as well as Canada and United States. Group 3 was frequent in Mediterranean countries in Europe and Northern Africa, Australia, and South America, where spring wheats are predominantly planted in the fall to better exploit winter precipitations. Group 4 was predominant in countries from Asia and is further subdivided into three distinct subgroups. Subgroup 4A was predominant among landraces and cultivated materials from Iran, Kazakhstan, Afghanistan, Tajikistan, and Pakistan on one side, and Taiwan and Japan on the other side. Subgroup 4B was found mainly in Western Asia (Iran, Armenia, and Turkey) and Saudi Arabia, whereas subgroup 4C was predominant in countries from Southern Asia (India, Nepal, and Pakistan).

The results from Ward’s clustering, the genotypic similarities, and the subgroup membership coefficients based on STRUCTURE are shown in Figure 2. The STRUCTURE membership coefficients revealed a high degree of admixture in a large number of accessions, particularly among cultivars and modern breeding materials (Figure 2). This result is consistent with the complex network of germplasm exchange that characterizes modern wheat breeding worldwide. It is important to point out that the proportions of subgroups presented in Figure 1 and in the clusters in Figure 2, represent the proportions of accessions in the NSGC spring wheat core collection, but may not necessarily represent the current composition of cultivars in a specific country or group.

### Spring wheat panel response to *Pst*

**Figure 3:**
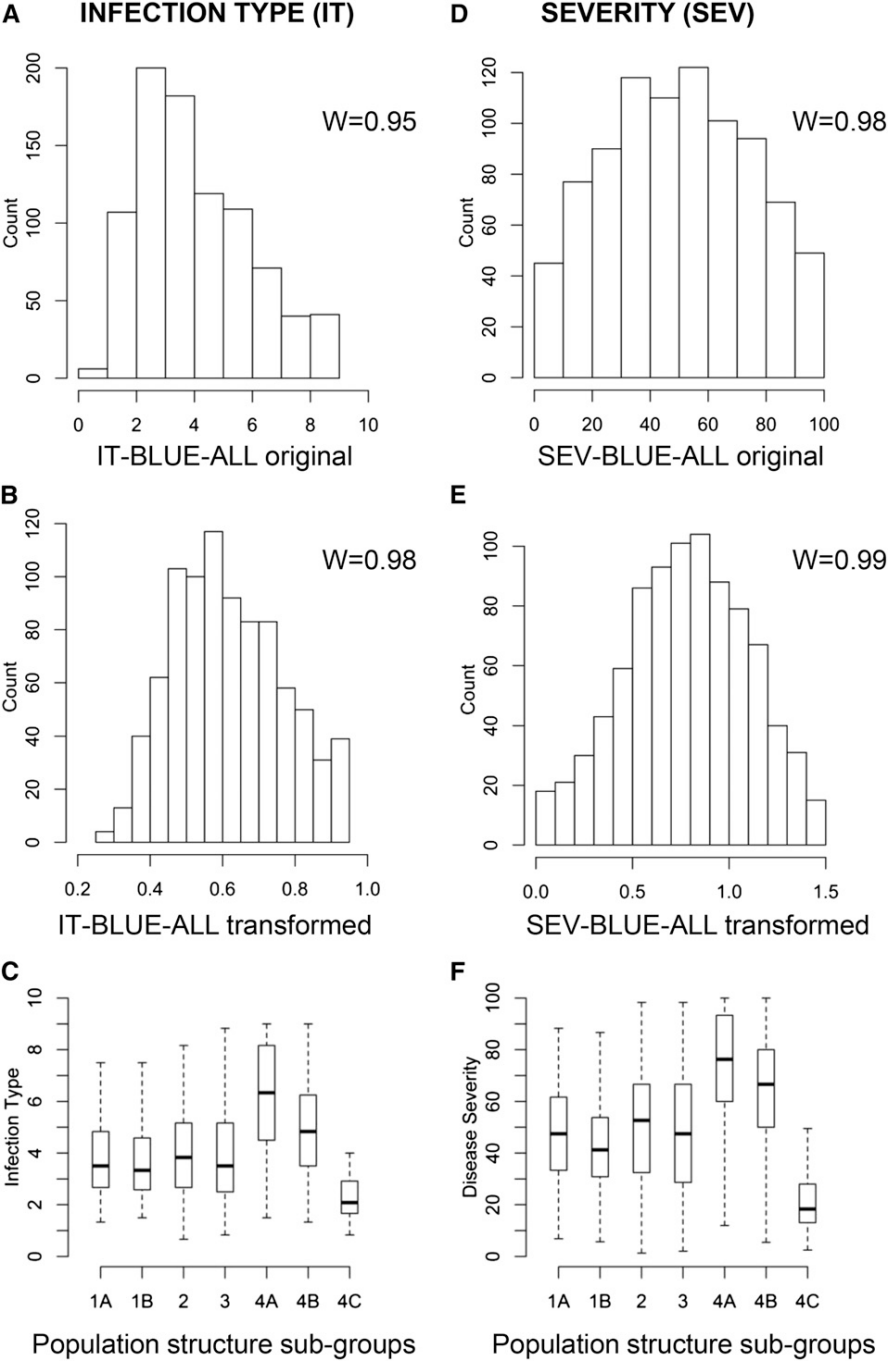
Distributions of infection type (IT) and disease severity (SEV). Distributions of best linear unbiased estimates (BLUEs) across all six environments for IT (A–B) and SEV (D–E). “W” indicates the correlation between observed values and normal scores for the original (A and D) and transformed (B and E) values. (C and F) Boxplot showing differences among subpopulations for IT (C) and SEV (F).

The response of the 875 selected accessions to *Pst* was evaluated in six environments (three locations × two years per location) characterized by very high infection levels of *Pst*. Phenotypic variation was observed in all environments, with infection types ranging from highly resistant (13% of accessions with IT 0−2) to highly susceptible (17% of accessions with IT 7−9). The frequency distributions of BLUE-all values for IT and SEV were approximately normal, with a slight shift in the IT response toward resistance (Figure 3, A and D). The square root and arcsine transformations used in this study improved the normality of the data (Figure 3, B and E). The analysis of variance (Table 1) showed that the variance components for genotype were highly significant (*P* ≤ 0.0001) for both the individual locations and the combined analysis across the six environments. By contrast, the variance components for environment were not significant across all analyses (Table 1). The variance component for the genotype by environment interaction was significant only in Davis (*P* ≤ 0.01 for IT and *P* ≤ 0.05 for SEV). Consistent with the previous results, heritability values for IT and SEV were high for the individual locations (0.72 to 0.87, Table 1) and even greater for BLUE values across all locations (*H^2^* for IT = 0.89 and for SEV = 0.91, Table 1).

The high IT and SEV heritability values indicate limited environmental variation relative to genotypic variation, an observation supported also by high and significant correlations among environments. Pearson correlation coefficients for IT averaged 0.73 ± 0.03 within locations and 0.65 ± 0.01 across locations (Table 2). Very similar correlations among environments were observed for SEV (*R* = 0.74 ± 0.05 within locations and 0.66 ± 0.01 across locations, Table 2). As expected, average correlations between SEV and IT values were the greatest within the same environment (*R* = 0.83 ± 0.02), intermediate within the same location in different years (*R* = 0.70 ± 0.03), and the lowest among locations (*R* = 0.63 ± 0.01, Table 2). The correlation between IT and SEV BLUE-all values was very high (*R* = 0.93, Table S3).

**Table 2:** Pearson’s correlation coefficients for IT and SEV response to *Puccinia striiformis* f. sp. *tritici* of 875 NSGC spring wheat accessions in six environments

	MTV-12	MTV-13	PLM-11	PLM-12	DVS-11	DVS-12
IT *vs.* IT*^a^*						
MTV-12		**0.78**	0.68	0.74	0.64	0.67
MTV-13			0.59	0.67	0.58	0.62
PLM-11				**0.73**	0.59	0.65
PLM-12					0.63	0.70
DVS-11						**0.67**
DVS-12						
SEV *vs.* SEV	MTV-12	MTV-13	PLM-11	PLM-12	DVS-11	DVS-12
MTV-12		**0.83**	0.68	0.72	0.65	0.72
MTV-13			0.68	0.66	0.62	0.67
PLM-11				**0.72**	0.55	0.65
PLM-12					0.58	0.68
DVS-11						**0.66**
DVS-12						
IT *vs.* SEV	MTV-12	MTV-13	PLM-11	PLM-12	DVS-11	DVS-12
MTV-12	0.86	**0.76**	0.68	0.66	0.60	0.71
MTV-13	**0.78**	0.76	0.68	0.64	0.58	0.64
PLM-11	0.58	0.55	0.80	**0.59**	0.49	0.62
PLM-12	0.70	0.67	**0.76**	0.82	0.56	0.69
DVS-11	0.62	0.59	0.58	0.58	0.85	**0.65**
DVS-12	0.66	0.62	0.65	0.68	**0.63**	0.87

Gray highlight indicates comparisons between the same location and year. Bold letters indicate comparisons between different years in the same location. All correlation coefficients are highly significant (*P* < 0.0001). IT, infection type; SEV, disease severity.

a Locations: MTV, Mount Vernon, WA; PLM, Pullman, WA; DVS, Davis, CA. Followed by year.

The extent to which the *Pst* response of the 875 different accessions was influenced by population structure is presented in Figure 2D and Figure 3, C and F. In Figure 2D the IT and SEV values of each accession are presented as heat-maps with blue indicating resistance and red susceptibility. A greater proportion of resistant accessions was evident in group 4C, whereas a greater proportion of susceptible accessions was observed in group 4A. A more balanced distribution of resistant and susceptible accessions was observed for the other subpopulations (Figure 2D). A similar trend was observed in the box plots for IT (Figure 3C) and SEV (Figure 3F). An ANOVA among subpopulations showed significantly greater IT and SEV averages in subpopulation 4C and significantly lower averages in subpopulation 4A (Tukey’s test *P* < 0.05, except for one nonsignificant SEV difference between subpopulations 4A and 4B). The association between population structure and *Pst* resistance also was reflected in significant multiple regressions (*P* ≤ 0.01) between BLUE-all values and population structure (Q7) for both IT (*R^2^=*0.141) and SEV (*R^2^=*0.166). These significant associations indicated that corrections for population structure were required for the GWAS. Comparison of different population structure correction methods (Figure S1 and the section *Material and Methods*) indicated that the use of the kinship matrix (K) was the most informative option for this dataset.

Corrections for plant height and heading time were evaluated to determine whether they influenced the GWAS results for *Pst* resistance. Both traits are critical for wheat performance in the field and are known to have multiple pleiotropic effects. However, in this study negligible correlations were observed between *Pst* resistance (IT and SEV BLUE values across all six environments) and both heading time (*R*^2^ < 0.005) and plant height (*R*^2^ < 0.05). Therefore, neither of these traits was included as covariate in the GWAS for *Pst* resistance. None of the high-confidence QTL selected in this study (Table 3) showed significant association with plant height or heading time. However, among the lower-confidence QTL, significant associations were detected for one heading time QTL (*IWA8513*) and five plant height QTL (*IWA692*, *IWA1923*, *IWA4347*, *IWA6630*, and *IWA3796*), which were excluded from Table S4.

**Table 3:** Loci significantly associated with IT or SEV in at least three environments (one at *P* < 0.01) and with experiment-wise Bonferroni *P* < 0.10

Marker IWA	***3892**^a^***	***980***	***422***	*424*^b^	***5202***	***1034***	*5375*	*6988*	***7257***	***167***
QTL name	*QYr.ucw-1B*	*QYr.ucw-1D*	*QYr.ucw-2A.2*	*QYr.ucw-2A.3*	*QYr.ucw-3B.2*	*QYr.ucw-4A*	*QYr.ucw-4D*	*QYr.ucw-5A.2*	*QYr.ucw-6B*	*QYr.ucw-6D*
Chromosome	1B	1D	2A	2A	3B	4A*^c^*	4D	5A*^d^*	6B	6D
Position*^e^*	123.4	49.3	9.9	78.3	3.9	181.7	26.9	189.2	112.3	73.2
Alleles (R underscored)	A/G	A/C	T/C	T/C	A/G	T/C	T/G	T/C	T/G	A/C
GWAS for IT										
MTV_2012	****^f^	ns	***	ns	**	*	****	*	**	****
MTV_2013	*	**	*	*	ns	**	*	ns	*	***
PLM_2011	*	*	****	ns	***	****	**	****	****	***
PLM_2012	ns	***	***	ns	****	*	ns	ns	****	**
DVS_2011	**	*	ns	ns	*	**	**	****	*	****
DVS_2012	*	ns	**	*	ns	*	**	**	***	****
BLUE_MTV	*	***	**	*	*	****	***	ns	**	****
BLUE_PLM	**	ns	****	ns	****	**	**	**	****	***
BLUE_DVS	****	**	*	ns	ns	**	***	***	***	****
BLUE_ALL	***	*	***	*	***	****	***	***	****	****
GWAS for SEV										
MTV_2012	***	ns	***	*	ns	*	***	ns	*	***
MTV_2013	**	*	**	*	ns	*	***	ns	*	***
PLM_2011	***	ns	**	***	***	**	*	ns	**	*
PLM_2012	***	****	****	***	*	ns	****	**	**	**
DVS_2011	*	ns	ns	ns	ns	**	***	***	*	****
DVS_2012	*	*	**	ns	ns	*	**	**	****	****
BLUE_MTV	***	*	**	*	ns	*	***	ns	*	***
BLUE_PLM	**	*	***	****	**	*	**	*	**	*
BLUE_DVS	****	**	*	*	ns	*	***	***	***	****
BLUE_ALL	****	*	***	**	ns	**	***	**	***	****
GWAS seedling										
Significant races	PSTv40	None	Pstv14	Pstv14 and 40	None	None	Pstv4	Pstv14	None	None
Frequency R allele										
Overall	0.70	0.44	0.31	0.70	0.37	0.17	0.10	0.16	0.23	0.10
Subpop. 1A	0.77	0.19	0.35	0.48	0.41	0.08	0.38	0.17	0.61	0.36
Subpop. 1B	0.87	0.17	0.10	0.83	0.52	**0.04**	0.12	0.10	0.32	0.10
Subpop. 2	0.70	0.70	0.19	0.80	0.47	**0.03**	**0.05**	0.12	0.51	**0.03**
Subpop.3	0.76	0.60	0.25	0.88	0.40	0.17	**0.02**	0.15	0.11	**0.06**
Subpop. 4A	0.53	0.34	0.56	0.26	**0.09**	0.39	0.10	0.38	**0.06**	**0.05**
Subpop. 4B	0.77	0.43	0.77	**0.97**	0.32	0.28	**0.01**	0.23	**0.01**	**0.01**
Subpop. 4C	0.70	0.20	**0.93**	0.35	0.12	**0.00**	0.88	0.88	**0.00**	**0.95**
% explained variation										
* R^2^* IT BLUE-all	0.7%	1.2%	0.4%	1.0%	2.2%	0.9%	0.9%	1.6%	1.8%	0.7%
* R^2^* SEV-BLUE-all	0.7%	0.9%	0.8%	0.3%	1.3%	1.2%	0.6%	1.0%	1.4%	0.7%

IT, infection type; SEV, disease severity.

a SNP indexes from the Illumina iSelect 9K wheat assay (Cavanagh *et al.* 2013). **Bold** IWA names were identified in the GWAS including only the 593 accession with IT scores ≥ 3, and **bold and underscored** were significant in both analyses. SNP loci in LD and significantly associated to the *Pst* response (IWA): 3892/846, 980/642, 422/423/3468/3469, 424/none, 5202/4796, 1034/none, 5375/5766, 6988/none, 7257/none, 167/none.

b *IWA424* was borderline significant in the Bonferroni test only for SEV and only in one location. Therefore, it should be considered with caution.

c *IWA1034* is in the region of 4AL translocated from 7BS (homology 7AS/4AL/7DS) (Devos *et al.* 1995).

d *IWA6988* is in the region of 5AL translocated from 4AL (homology 5AL/4BL/4DL) (Devos *et al.* 1995).

e Scaled position from hexaploid wheat consensus map (Cavanagh *et al.* 2013).

f Significance. Marker-wise: *P < 0.05, **P < 0.01, ***P < 0.001. Experiment-wise: ****Bonferroni *P* < 0.10.

### Spring wheat panel LD estimation

**Figure 4:**
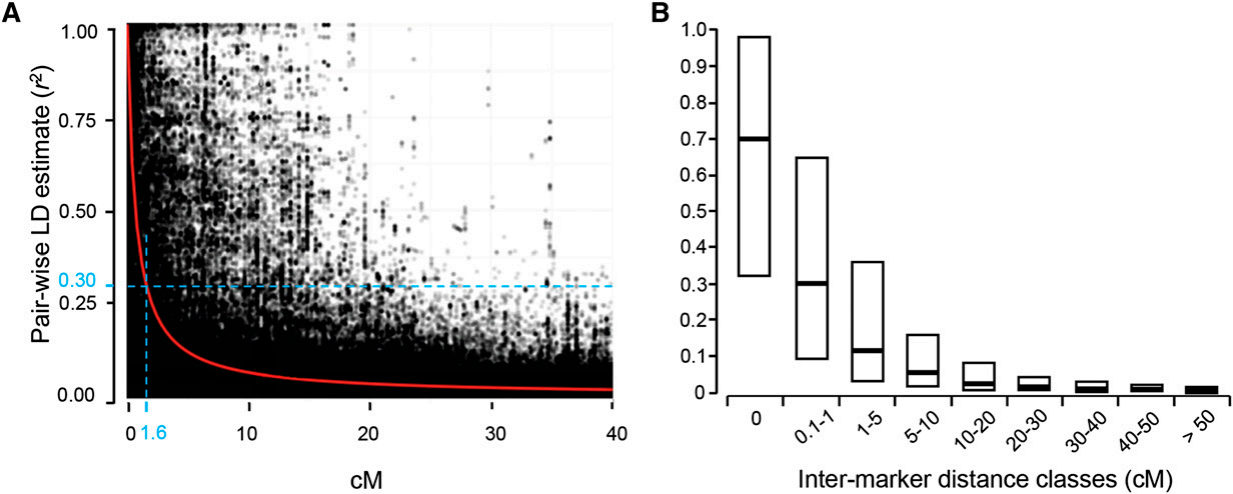
Genome-wide average linkage disequilibrium (LD) decay over genetic distances. (A) Plot of pair-wise single-nucleotide polymorphism LD *r*^2^ values as a function of intermarker map distance (cM) based on a reference consensus map (Cavanagh *et al.* 2013). The red curve represents the model fit to LD decay. The light-blue dashed line represents the ±1.6 cM confidence interval for the quantitative trait loci regions in which LD *r*^2^ = 0.3. (B) Boxplot showing LD *r*^2^ values for incremental classes of single-nucleotide polymorphism pairwise map distances.

The extent of LD and the average trend of LD decay rate in this association panel were estimated based on pairwise LD squared correlation coefficients (*r*^2^) for all intrachromosomal SNP loci. The trend of LD decay was described by a nonlinear regression of the pair-wise *r*^2^ values on the corresponding map distances based on the Illumina 9K SNP consensus map (Cavanagh *et al.* 2013) and by a box-plot diagram of LD *r*^2^ distribution (Figure 4). Among the pairs of markers that were completely linked in the consensus map the median LD *r*^2^ was 0.69 (inter-quartiles ranging from 0.32 to 0.98). In the next class, including noncompletely linked markers separated by less than 1 cM, the median LD *r*^2^ decreased to 0.3. Thus, on average, a 50% LD decay rate was observed within 1 cM genetic distance. For the pairs of markers linked at 1−5 cM the median *r*^2^ decreased to 0.12.

Similar results were obtained by fitting a nonlinear regression equation (Sved 1971). Based on the fitted model, LD was predicted to fall below the nominal critical levels of *r*^2^ = 0.3 at an inter-marker genetic distance of 1.6 cM (Figure 4). This 1.6 cM distance at each side of the peak of the significant associations was used to establish confidence intervals for the QTL-harboring regions.

### Association analyses for *Pst* resistance across environments

Association analysis was performed separately for each of the six field environments to identify chromosome regions including *Pst* resistance genes effective against different combinations of *Pst* races. A total of 73 chromosome regions showed marker-wise significant associations with either IT or SEV in at least three environments (Table S4 and Figure S2). Among these QTL only seven were significant at the experiment-wise Bonferroni threshold of *P* < 0.10 and are described in Table 3.

To test whether additional partial resistance QTL were masked by the major resistance genes segregating in this panel, we performed a second set of GWAS in which 282 accessions with highly resistant infection types (ITs of 0−3) were not included in the analyses. GWAS using the remaining 593 accessions with IT > 3 revealed 35 QTL showing marker-wise significant associations with either IT or SEV in at least three environments (Table S5 and Figure S3). Eleven of these 35 QTL overlapped with the previous set of 73 and 24 were new (Table S5). Seven of the 35 QTL were significant at the experiment-wise Bonferroni threshold (*P* < 0.10). Among these seven, four overlapped with the ones identified in the previous GWAS (Table S4), and three (*IWA3892*, *IWA980*, and *IWA1034*) were new and were added to Table 3.

**Figure 5:**
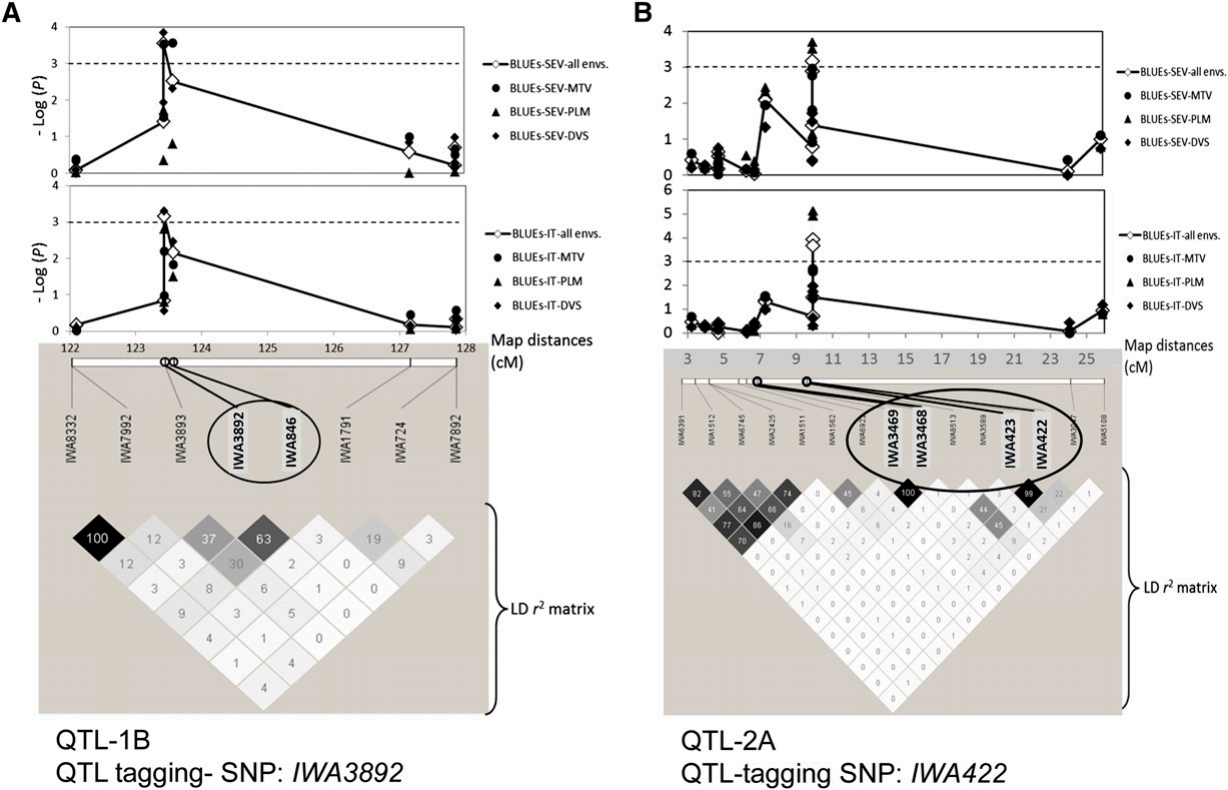
*P* value association plots and corresponding linkage disequilibrium (LD) *r*^2^ patterns for two significant *Pst* resistance quantitative trait loci (QTL). The upper part of the graph shows *P* value plots of marker-trait associations for best linear unbiased estimates (BLUEs) of infection types (IT) and disease severity (SEV) over six environments and for the three specific locations (MTV, Mount Vernon; PLM, Pullman; DVS, Davis). Map distances (X-axis) are from the 9K SNP consensus map (Cavanagh *et al.* 2013). –Log (*P*) significance thresholds are reported using dashed lines. Single-nucleotide polymorphism (SNP) codes and corresponding local LD *r*^2^ value patterns are in the lower part of the graph. Numbers within the diamonds of the triangular LD matrix are the *r^2^* values multiplied by 100.

Detailed information for the 10 significant loci is presented in Table 3, which includes the representative SNP, the suggested name for the QTL, and the probabilities of association with IT and SEV values from the six individual environments and BLUE values. Table 3 also includes the favorable allele and its frequency in the complete association panel and in each of the seven subpopulations. Five of the 10 significant loci had closely linked SNPs (< 1.6 cM and in LD to each other) that also showed significant associations with *Pst* resistance (Table S4 and Table S5), providing additional support for the association between that chromosome region and resistance to *Pst*. Two of these QTL are described in more detail in Figure 5. In this figure, the significance of the phenotype-SNP association (as –log *P*) is plotted along the consensus map (Cavanagh *et al.* 2013) and compared with local *r*^2^ LD patterns below. SNP-phenotype associations rapidly decayed within 1-2 cM, in accordance with the observed trend of LD decay rate estimated from SNP pairs (Figure 4).

Table 3 also indicates which of the 10 significant QTL identified in the field studies were also significantly associated with resistance to the four individual *Pst* races tested at the seedling stage in controlled environments. Five of the 10 loci were significant for both the adult plant (field studies) and seedling tests (controlled environment). A similar proportion was observed among the lower-confidence QTL (47%; Table S4). QTL identified in the second GWAS for partial resistance showed a lower proportion of loci that were significant in the seedling tests (30%; Table S5). The levels of significance of the adult plant and seedling QTL were generally similar, except for *IWA4280* that showed exceptionally strong associations in the seedling tests (*P* < 10^−24^) but only moderate associations in the field experiments (Table S4).

The frequencies of favorable alleles in the different subpopulations for the 10 significant QTL-tagging SNPs identified from the multienvironmental trials are also summarized in Table 3. Similar frequency information is also available for lower-confidence QTL that were significant in at least three environments (Table S6 and Table S7). Favorable alleles for QTL associated with markers *IWA167*, *IWA5375*, and *IWA6988* were present at high frequency in subpopulation 4C (Pakistan-India-Nepal) and at relatively low frequencies in most of the other subpopulations. The favorable allele for *IWA422* was present at relatively high frequency among the three Asian subpopulations (4A, 4B, and 4C) and at lower frequencies in other subpopulations. The opposite profile was observed for *IWA7257* that was present at very low frequency in the three Asian subpopulations and higher frequencies in the other subpopulations. Subpopulation 4C showed a relatively higher proportion of favorable alleles with very high or very low frequencies, a trend associated with the lowest genetic diversity of subpopulation 4C among the seven subpopulations (Table S6).

The proportion of phenotypic variance explained by the individual QTL for IT and SEV BLUE values across all environments ranged from 0.4 to 2.2% (Table 3). When these 10 QTL were analyzed together in an ANOVA including population structure as covariates (Q7), they explained 15% and 12% of the observed variance for IT and SEV BLUEs across all environments, respectively (excluding variation explained by the population structure; Table S8). When the six significant interactions among loci were added to the model, the proportion of explained phenotypic variation (excluding population structure) increased to 19% for IT and to 16% for SEV BLUE values across all environments (Table S8). Interestingly, when the interactions were added to the model, the highly significant effects of *IWA422* and *IWA5202* became non-significant. These two markers were involved in four of the six significant interactions that are presented in Figure S5. In general, the presence of the resistance allele from one locus resulted in a reduced effect for the interacting locus (Figure S5, A−E). However, in the *IWA6988* × *IWA167* interaction, the presence of the susceptible alleles from one of these two loci was associated with a reduction or elimination of the effect of the interacting locus (Figure S5F).

Two markers tightly linked to known *Pst* resistance genes on chromosome arms 7DS (*Lr34/Yr18/Pm38*) and 4DL (*Lr67/Yr46*) were used as controls. The scoring of these two markers, their linkage with other SNPs, and their association with *Pst* resistance traits are described in supplemental File S3. Diagnostic marker *KaspLr34* showed a MAF of 17.8% (resistant allele), which is higher than the 10% threshold selected in this study. The *KaspLr34* marker was significantly associated to IT and SEV responses for three of the six environments (DVS_2012, MTV_2012, MTV_2013), and adjusted averages across all environments were significant experiment-wise (Bonferroni < 0.10). *KaspLr34* did not show appreciable LD (maximum *r*^2^ = 0.04, File S3) with any of the 22 polymorphic SNPs mapped on chromosome 7D.

Marker *Kasp856*, which is tightly linked to *Lr67/Yr46* (chromosome 4DL), showed a MAF of 8.6%, and therefore, it would have been excluded from our GWAS. This marker was strongly associated to *Pst* severity values in DVS-2011, DVS-2012, and MTV-2013, and also showed experiment-wise significant associations to the adjusted averages across all environments. *Kasp856* was in LD to *IWA5375* (*r*^2^ value = 0.41, MAF = 10%; File S3), which was found in our GWAS to be significantly associated to *Pst* resistance. Markers *IWA6277*, *IWA5381*, *IWA5766*, *IWA5375*, and *Kasp856* defined three main haplotypes within a 6.3 cM region. The haplotype G-A-G-T-A at these five markers was associated to *Pst* resistance, supporting the results obtained from the single SNP markers (File S3).

### Relationship between number of favorable alleles and *Pst* resistance

Considering all QTL-tagging SNPs with marker-wise significant effects in at least three environments (97 in total, including the 24 for partial resistance), the number of favorable alleles present in a specific accession ranged from 23 to 65. The genetic profiles of the accessions for the 97 QTL-tagging SNPs are reported in File S1, where accessions were ranked based on the number of favorable alleles. The 87 accessions (10%) with the highest number of favorable alleles (favorable alleles = 56.6 ± 0.3) showed significantly lower (*P* < 0.0001) IT (2.3 ± 0.1) and SEV (22.5% ± 1.8%) values than the 87 accessions with the lowest number of favorable alleles (favorable alleles = 34.7 ± 0.3, IT = 6.8 ± 0.2, SEV = 79.0 ± 2.0). These significant associations were also reflected in highly significant correlations (*P* < 0.0001) between the number of favorable alleles and both IT (*R =* 0.68) and SEV (*R =* 0.67) values. This result indicates that the variation in the number of favorable alleles in these 97 QTL explains 45–46% of the variation in *Pst* resistance in this germplasm collection, excluding variation explained by population structure (Figure S4). Thus, this dataset may be a powerful tool for genomic predictions of stripe rust resistance.

### Comparison of significant QTL with rice and *Brachypodium* genomes

To facilitate the identification of additional markers and to accelerate the discovery of potential candidate genes, we established the colinearity between confidence intervals for the 10 QTL identified in this study and the sequenced *Brachypodium* and rice genomes (File S2). As a first step, we increased the number of markers in the QTL-confidence intervals by projecting the wheat SNP consensus map generated from the Illumina 90K assay (including 40,269 SNPs) onto the reference 9K Illumina consensus map using the program BIOMERCATOR v4.2 (Sosnowski *et al.* 2012). On average, 12 SNP markers from the 9K assay and 80 projected from the 90K assay (total 92) were detected per QTL confidence interval (Table S10). Sequences of the original and projected wheat SNP markers were then used to find the best hits in *Brachypodium* and rice.

The *Brachypodium* and rice synteny relationships for the 10 QTL confidence intervals are described in detail in File S2. On average, 60 *Brachypodium* and rice colinear annotated genes were identified per confidence interval (Table S10). Based on the Phytozome v9.1 database (http://www.phytozome.net/) 12.7% of the annotated colinear genes were classified as *R* genes (CC-NB-LRR, RLP and RLK), with higher proportions found in the confidence intervals for QTL *IWA3892*, *IWA422*, *IWA5202*, and *IWA1034* (Table S10).

### Comparison of significant QTL with known *Pst* resistance genes

**Figure 6:**
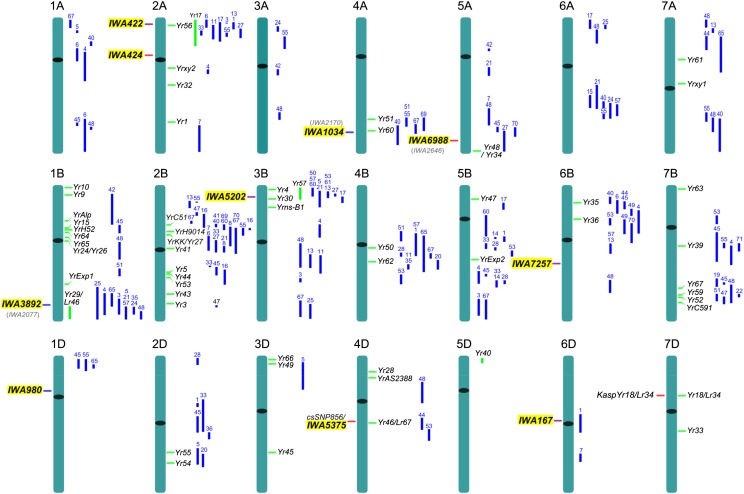
Chromosome positions of *Pst*-associated quantitative trait loci (QTL) identified in this study (experiment-wise Bonferroni *P* < 0.1) relative to previously mapped *Pst* resistance genes and QTL. Chromosome lengths were all standardized to the same relative length. QTL-tagging single-nucleotide polymorphisms identified in this genome-wide association (GWAS) are on the left side of the chromosomes. Those with a red line were significant only in the overall GWAS, those with a blue line only in the GWAS for partial resistance, and those with a purple line were significant in both (Table 3). Previously mapped *Pst* resistance genes (green) and QTL (blue) are on the right side of the chromosomes. The ID numbers on top of the QTL indicate references and confidence intervals provided in File S5.

To identify which of the 10 QTL-tagging SNPs described in Table 3 mapped on regions similar to 227 previously identified *Pst* resistance genes (*Yr*) and QTL, we projected both sets of markers on an integrated map including different types of markers (File S4). Figure 6 shows the projection of these resistance loci onto standardized chromosomes of similar length. The 10 highly-significant QTL identified in this study are presented to the left of the chromosomes and previously mapped *Pst* resistance genes and QTL to the right. The numbers on top of the QTL (Figure 6, blue bars) indicate the reference used to determine the confidence interval for the QTL. These references, together with the confidence intervals (in % length) are summarized in File S5.

Three of the 10 QTL-tagging markers reported in Table 3 (*IWA980*, *IWA424*, and *IWA7257*) were mapped far from any currently known *Pst* resistance gene or QTL, and likely represent novel resistance loci (Figure 6 and File S5). *IWA167* overlapped with *QYr.ufs-6D* (Figure 6), but the peaks of these two QTL were mapped on different chromosome arms and showed effects of different magnitude (File S5), suggesting that they likely represent different genes.

The other six QTL-tagging markers were found in the proximity (less than one tenth of the chromosome length) of named *Yr* resistance genes or previously mapped *Pst* resistance QTL (Figure 6). *IWA422*, *IWA5202*, and *IWA5375* were mapped less than 3 cM from previously mapped *Pst* resistance genes (Figure 6 and File S5), suggesting that they may represent alleles of the same genes. *IWA422* and *IWA5202* were mapped to the distal regions of chromosome arms 2AS and 3BS respectively, which include several previously mapped *Pst* resistance genes and QTL that can correspond to these 2 QTL (Figure 6 and File S5). *IWA5375* was mapped in the proximity of the *Lr67/Yr46* region, and likely represents the same locus since no other *Pst* resistance genes or QTL were mapped in their proximity.

The other three QTL-tagging markers (*IWA3892*, *IWA1034*, and *IWA6988*) were mapped within the confidence intervals of previously mapped *Pst* resistance genes or QTL (Figure 6 and File S5). However, for each of these three QTL we found a second closely linked SNP that showed marker-wise significant associations with *Pst* resistance in at least three of the locations evaluated in this study (Table S4, gray letters in parenthesis in Figure 6). The relationship between each of these three pairs of linked SNP with previously mapped *Pst* resistance genes or QTL is discussed in detail in supplemental File S5.

## Discussion

### Population genetic structure

The population structure of 1000 accessions from the spring wheat core collection was used as covariate in the GWAS mainly to reduce the number of false associations (Yu *et al.* 2006), but it also provided additional valuable information. First, the genetic characterization of the 1,000 spring lines revealed the existence of ~100 near-identical lines (>99% identical) in this panel, which were eliminated from our analyses (together with another 25 with >10% missing data). The elimination of the near-identical lines from the NSGC spring wheat core collection can reduce the size of the collection without a significant loss of genetic diversity.

In addition, the genetic characterization of these accessions organized the spring wheat core collection into seven subpopulations of genetically related accessions (Figures 1 and 2). The Ward clustering analysis (Figure 2) revealed a major division between the subpopulations enriched in accessions from Asia (4A, 4B, and 4C, henceforth “Asian” subpopulations) and subpopulations that include a large proportion of accessions from other parts of the world (1A, 1B, 2, and 3, henceforth “Western” subpopulations). This division was also observed in a study of SNP diversity in the D-genome (Wang *et al.* 2013), and may reflect the ancestral eastward and westward expansion of agriculture (and of domesticated *T. aestivum*) after its initial domestication in the Fertile Crescent roughly 10,000 years ago (Dubcovsky and Dvorak 2007).

This major division was further supported by pairwise comparisons among subpopulations using *Fst* (Table S9), which provides a measure of population differentiation due to genetic structure (Holsinger and Weir 2009). Pairwise comparisons among the four “Western” subpopulations showed smaller *Fst* values than comparisons between the “Western” and “Asian” subpopulations, or among the “Asian” subpopulations (Table S9). Among the “Asian” subpopulations, group 4C was the most divergent (Table S9). This subpopulation is the smallest (44 accessions) and the less diverse one, with an average polymorphism information content that is roughly half of the averages observed in the other six subpopulations (Table S6).

The “Western” subpopulations include a large proportion of materials released after 1960 (55%), which were likely influenced by the Green Revolution. CIMMYT (Centro Internacional de Mejoramiento de Maíz y Trigo) played a central role in the development and distribution of these Green Revolution varieties, and accounts for 90% of the accessions from Mexico presented in Figure 1. CIMMYT lines in this collection belong mainly to the four “Western” subpopulations, which may explain the abundance of these subpopulations in regions that frequently receive or exchange germplasm with CIMMYT. Many accessions included in the “Western” subpopulations show evidence of high levels of admixture (Figure 2), which likely reflects the frequent germplasm exchanges among wheat breeding programs from these regions. A greater level of admixture among spring wheat varieties than among winter wheat varieties has been reported also in a different germplasm panel (Cavanagh *et al.* 2013).

### Association between population genetic structure and *Pst* resistance

Subpopulations1A, 1B, 2, and 3 show a uniform distribution of *Pst* IT and SEV values (Figure 3, C and F), possibly an additional reflection of the extensive admixture observed in these populations. By contrast, a significant association between population structure and *Pst* response was observed for the three subpopulations from Asia (Figure 3). Accessions from India, Nepal, and Pakistan in subpopulation 4C displayed a greater proportion of moderately resistant to resistant phenotypes than any of the other six subpopulations (Figure 3). Interestingly, a recent study of the ancestral relationships among worldwide populations of *Pst* has pointed to the same Himalayan and neighboring region as the putative center of origin for *Pst*. This hypothesis was supported by the existence of high levels of diversity, presence of private alleles, clear signatures of recombination, and ability to produce sex-related structures in the *Pst* races from this region (Ali *et al.* 2014). Archeological remains indicate that hexaploid wheat was already cultivated in India-Pakistan between 4000 and 2000 BC (Tengberg 1999), which suggests that wheat populations from this region may have the longest history of interactions with *Pst*.

The ancient *T. aestivum* L. ssp. *sphaerococcum* (Percival) Mac Key (synonym: *T. sphaerococcum* Percival) endemic to southern Pakistan and northwestern India was described in the early 1920s as an early flowering semidwarf wheat with semispherical grains and with resistance to yellow rust (Percival 1921). This description indicates that sources of resistance to *Pst* had already evolved in this region before the introduction of modern wheat varieties. Several of the *Pst* resistance QTL identified in this study (*IWA167*, *IWA6988*, *IWA5375*, and *IWA422*) are present at higher frequencies in subpopulation 4C than in any of the other subpopulations (Table 3) and may represent valuable alleles to enrich the “Western” subpopulation with novel or infrequent resistance alleles.

By contrast, subpopulation 4A showed the lowest levels of resistance (Figure 3), suggesting that regions in which varieties from this subpopulation are grown may be at a greater risk of *Pst* epidemics. Regions where subpopulation 4A is at high frequency (Figure 1) may benefit from the incorporation of resistance alleles identified in this study that are absent or at very low frequency in the 4A subpopulation (Table 3, Figure S6, and Figure S7). The observed heterogeneity of *Pst* resistance levels among subpopulations (Figure 3, C and F) poses additional challenges for the discovery of real associations by GWAS. Adjustment of the GWAS analysis for population structure can reduce this problem, as demonstrated in previous studies in rice (Zhao *et al.* 2011) and maize (Van Inghelandt *et al.* 2012), where similar levels of associations between phenotypes and population structure were reported.

The benefits of correcting for population structure are partially offset by an increase in false negatives. Some real associations that are highly correlated with the population structure can be lost in GWAS analyses corrected for population structure. Similarly, the increased protection for the identification of false positives achieved in this study by the elimination of SNPs with MAFs lower than 0.1 is offset by the inability to detect real resistance genes with low allele frequencies. In this initial study, we favored the more stringent criteria. However, alternative analyses can be performed using this dataset, which is publicly available through the T3 database (http://triticeaetoolbox.org/) and the ARS-GRIN system (http://www.ars-grin.gov/).

### Significant associations in the GWAS

To identify new sources of resistance to *Pst* that were effective in different environments of the western United States, we performed field evaluations in three locations with very different ecological conditions. Despite these differences, we observed high correlations among IT and SEV values obtained from the different environments (Table 2). These high correlations suggest that there might be similar *Pst* populations across the western United States. This hypothesis is supported by the known paths of spore dispersal by wind (Chen 2005) and by periodic spore surveys across this region. In the last published *Pst* race survey from 2010 (Wan and Chen 2014), 20% of the races detected in California and Washington were shared between the two states (PSTv-8, PSTv-14, PSTv-36, PSTv-37, PSTv-40, and PSTv-41), providing further support to the previous hypothesis. The high correlations among environments were also reflected in high heritability for IT and SEV values (Table 1), which were favorable for the identification of significant associations in the GWAS analyses.

Even with the high heritability values observed in this study, only 10 QTL were significant at the experiment-wise Bonferroni threshold of *P* < 0.10 selected for this study (Table 3). Among these 10 significant QTL, seven were detected in the GWAS based on all 875 accessions and three in the second GWAS that excluded accessions with high levels of resistance. This suggests that the GWAS for partial resistance provided additional power to detect associations that were somehow masked by the effect of the major resistance genes. A similar proportion of additional loci were detected among the lower confidence QTL for partial resistance (24 of 97, Table S4 and Table S5). Among the SNPs associated with QTL for partial resistance a smaller proportion of loci were also effective at the seedling stage (30%) compared with the proportion observed in the complete GWAS analysis (47%). This is not surprising because many partial resistance genes are effective only at the adult plant stage. In summary, elimination of highly resistant accessions from a GWAS for disease resistance was a useful strategy to identify additional partial resistance genes. The incorporation and deployment of partial resistance genes is an important objective for wheat breeding programs because this type of resistance genes have historically provided more durable resistance than race specific resistance genes (Johnson 1984; Kolmer 1996; Santra *et al.* 2008; Krattinger *et al.* 2009; Lowe *et al.* 2011a; Chen 2013).

The two markers from known *Pst* resistance genes *Yr18* (*Lr34/Yr18/Pm38*) and *Yr46* (*Lr67/Yr46*) included as controls in a separate GWAS, both showed experiment-wise significant associations with *Pst* resistance (*P* < 1 e^-4^, File S4). No linked markers were identified in our study for *Yr18* suggesting that the 9K iSelect chip did not provide enough coverage in the D genome to detect all *Pst* resistance loci present in our panel. The *Yr46* marker was closely linked with markers identified in our study that were significantly associated to *Pst* resistance. However, the *Kasp856* marker for *Yr46* would have been excluded in our original GWAS because its MAF was below our selected 10% MAF threshold. Fortunately, some of the SNPs linked to *Kasp856* have MAF greater than 10% and the QTL was detected. This result exemplifies the potential loss in sensitivity associated with increased stringency.

The proportion of variation in IT values explained by each of the 10 selected QTL was relatively small (0.4–2.2%, Table 3) and similar to values previously reported in GWAS for other quantitative traits in maize and rice (Zhao *et al.* 2011; Peiffer *et al.* 2013; Peiffer *et al.* 2014). When the 10 QTL were combined in a single ANOVA, 15% of the variation (Table S8) in *Pst* resistance was explained by the model (excluding the contribution of the population structure). This percentage increased to 19% when six significant two-way interactions were added to the ANOVA model (Table S8 and Figure S5). We have initiated the introgression of these loci into the common susceptible background “Avocet S” to test these interactions experimentally.

In this study we also identified 87 QTL that showed significant GWAS associations in at least three environments but that did not pass the stringent experiment-wise Bonferroni threshold. These loci may have a greater proportion of false positives and were, therefore, excluded from the results reported in Table 3. However, the inclusion of all 97 QTL in a combined ANOVA model increased the percent of explained variation in IT BLUE-all values from 15 to 45% (excluding variation explained by population structure). These results suggest that some of the lower-confidence QTL likely represent real associations. As a compromise, we included the 87 lower-confidence QTL in Table S4 and Table S5, as candidates for future validation studies.

The presence of real resistance genes among the lower-confidence QTL is also supported by a high and significant correlation (*R* = 0.68) between the number of favorable *Pst* alleles (among the 97 loci) and the level of *Pst* resistance (File S1 and Figure S4). This high correlation suggests that accessions carrying a high number of favorable alleles for the different QTL identified in this study may be useful parental lines for breeding programs interested in diversifying their sources of *Pst* resistance genes. This high correlation also suggests that a genomic selection approach aimed at increasing the number of favorable *Pst* alleles for the QTL identified in this study is likely to increase the levels of *Pst* resistance in the breeding populations.

The results discussed above suggest that *Pst* resistance in the spring wheat core collection was governed by several resistance genes with relatively larger effects (Table 3) modulated by a larger number of genes with smaller effects (Table S4 and Table S5). This trait architecture is different from the architecture found for resistance to downy mildew in a previous GWAS in Arabidopsis. The Arabidopsis study, which tested resistance at the seedling level using specific races and in controlled environmental facilities, detected few dominant resistance genes that were concentrated in only four genomic regions (Nemri *et al.* 2010). The larger association panel used in our study and the increased statistical power may have contributed to the higher number of QTL identified in this study. This greater number of detected QTL also may be the result of the more complex environmental conditions found in the field relative to the controlled environment. In the field, the frequency of infection and the rate of development of the pathogen can be modulated by different morphological and physiological characteristics of the plant and also by the simultaneous presence of multiple *Pst* races and other pathogens. In addition, the use of adult plants in our field studies can result in the detection of additional adult plant resistance genes that are not effective at the seedling stage.

### Comparison of significant QTL with colinear genes in rice and *Brachypodium*

The LD decay rate between the 9K Illumina SNP (50% LD decay rate at 1 cM) in this association panel was faster than that observed in elite US spring wheat (50% LD decay rate at 6.3 cM; Chao *et al.* 2010). This is not surprising because this elite US spring wheat panel includes a less diverse germplasm than the NSGC spring wheat core collection analyzed in this study and therefore has a lower level of historical recombination. The intermarker distance at which LD fall below the critical levels of *r^2^ =* 0.3 (1.6 cM) was selected to determine confidence intervals for the 10 significant QTL identified in Table 3.

The projection of the 90K Illumina data (Wang *et al.* 2014) onto these 10 QTL confidence intervals resulted in a 7.7-fold increase in the number of SNP per confidence interval relative to the initial results from the 9K chip map. The larger number of markers available per confidence interval (on average 92 markers) facilitated the identification of colinear regions in the sequenced genomes of the grass species *Brachypodium* and rice (File S2). The annotation of the proteins encoded by the *Brachypodium* and rice colinear genes was used to infer the putative function of the wheat genes from where the SNPs were derived. Among the wheat SNPs with a corresponding annotated rice or *Brachypodium* protein (Table S10), 12.7% were classified as *R* genes with two large groups in the confidence intervals for QTL *IWA422* and *IWA5202* (Table S10). Interestingly, the colinear regions for *IWA422* (*Brachypodium* Chr. 5, 1.43−4.20 Mb and rice Chr. 4, 0.07−6.96 Mb) and for *IWA5202* (*Brachypodium* Chr. 2, 0.74−0.82 Mb and rice Chr. 4, 0.72−0.92 Mb) include several NB-LRR and LRR-receptor-like kinases. These results suggest that this region may include an ancestral *R* gene cluster that predates the divergence of the grass subfamilies. It would be interesting to investigate if the multiple *Pst* resistance genes and QTL mapped to the colinear distal regions of wheat chromosome arms 2AS and 3BS (Figure 6 and File S5) are associated with the presence of similar clusters of *R* genes in wheat.

In summary, these comparative analyses provided a large number of molecular markers for each of the 10 targeted regions, which can be used both to identify haplotypes associated with resistance alleles, and to accelerate the construction of high-density maps for these QTL. In addition, pathogen-response related genes identified in the colinear regions in *Brachypodium* and rice may provide a starting point for the search of candidate genes in the colinear regions in wheat.

### Comparison of significant QTL with previously mapped genes

The results presented in Figure 6, provide an overview of the relationships between the loci identified in this GWAS and 227 previously mapped *Pst* resistance genes and QTL. However, these results should be considered with caution because of the inherent limitations of consensus maps. The limited number of common markers between previous SSR-based and new SNP-based maps can result in distorted distances in some region of the integrated map. The comparison also is complicated by the low resolution of some of the original maps of *Pst* resistance genes and by the extended LD in wheat. Therefore, the relationships described in the two paragraphs below and in File S5 should be considered as tentative. For closely linked loci, allelism tests will be required to determine which of the QTL identified here are new resistance loci and which are alleles of previously identified genes.

Among the 10 significant QTL reported in Table 3, *IWA980*, *IWA424*, and *IWA7257* were mapped on chromosome regions where, to our knowledge, no *Pst* resistance genes or QTL were reported before. These results suggest that they are likely novel *Pst* resistance loci (Figure 6). *IWA167*, one of the most significant QTL detected in this study, was mapped within the flanking markers of a weak QTL from Cappelle Desprez (*QYr.ufs-6D*; Agenbag *et al.* 2012). However, the peak of *QYr.ufs-6D* was mapped on the long arm of chromosome 6D, whereas *IWA167* is in the short arm. The different arm locations and the different strength of these two QTL suggest that they are likely the effect of different resistance genes (File S5). The *IWA167* resistance allele was almost fixed in the subpopulation from South Asia (frequency = 0.95) and was found at very low frequency in some of the other subpopulations, suggesting that it might be a useful gene to diversify sources of resistance in wheat breeding programs outside South Asia.

QTL associated with *IWA422*, *IWA5202*, and *IWA5375* were very close to previously mapped *Pst* resistance genes (< 3 cM) and likely represent alleles of these genes (File S5). The results from the last three significant loci (*IWA3892*, *IWA1034*, and *IWA6988*) are more difficult to interpret because each of them has a linked SNP marker that was also significantly associated (marker-wise) with *Pst* resistance in most of the locations tested in this study and will require additional allelism tests. In spite of these uncertainties, Figure 6 provides a good overview of the distribution of *Pst* resistance genes on the wheat chromosomes.

Since 2000, new aggressive strains of *Pst* were identified in three continents: North America, Australia, and Europe (Hovmoller *et al.* 2008). These races have increased aggressiveness and the ability to produce more spores at greater temperatures than before (Milus *et al.* 2009). These new races with broader spectra of virulences have generated serious stripe rust epidemics in areas previously considered unsuitable for the disease, and have turned stripe rust into one of the most economically damaging wheat pathogens (Hodson 2011). Therefore, the identification of novel *Pst* resistance genes in this GWAS represents a valuable addition to the set of tools available to wheat breeding programs to fight this devastating pathogen.

This GWAS also provides a good overview of the distribution of resistance genes and the frequencies of the resistance alleles in different wheat subpopulations around the world. The frequency information is particularly useful for wheat breeders interested in diversifying the sources of *Pst* resistance in their regional programs. In particular, wheat germplasm from the Himalayan and neighboring regions emerged from this study as a valuable source of resistance genes that are absent or present at low frequencies in other regions of the world.

As in other association studies, additional experimental validation will be required to identify which of the accessions carrying favorable SNP alleles actually carry the associated resistance gene. Allelism tests also will be required to determine which of the identified QTL represent novel resistance genes and which ones are alleles of previously mapped genes. To initiate this validation process we have crossed several accessions carrying favorable alleles for the selected QTL with the susceptible variety “Avocet S”, which is the common genetic background in the current single gene differential lines (Chen *et al.* 2014). These single QTL introgressions in a common genetic background will greatly simplify the planned allelism tests. In summary, this GWAS study has identified new sources of *Pst* resistance and provided closely linked markers to accelerate their validation and deployment in wheat breeding programs.

## Supplementary Material

Supporting Information
