# Supplementary material for: A Genome-Wide Association Study of Resistance to Stripe Rust (Puccinia striiformis f. sp. tritici) in a Worldwide Collection of Hexaploid Spring Wheat (Triticum aestivum L.)
Source: G3 (Bethesda). 2015 Jan 20;5(3):449–65. doi: 10.1534/g3.114.014563 (PMC4349098; doi:10.1534/g3.114.014563)
Supplement: Supporting Information [file supp_5_3_449__index.html]

A Genome-Wide Association Study of Resistance to Stripe Rust (Puccinia striiformis f. sp. tritici) in a Worldwide Collection of Hexaploid Spring Wheat (Triticum aestivum L.) — Supporting Information 

# A Genome-Wide Association Study of Resistance to Stripe Rust (*Puccinia striiformis* f. sp. *tritici*) in a Worldwide Collection of Hexaploid Spring Wheat (*Triticum aestivum* L.)

## Supporting Information for Maccaferri *et al.*, 2015

**Files in this Data Supplement:**

- Supporting Information - Tables S1-S10, Figures S1-S5, and Files S1-S5 (PDF, 526 KB)
- Table S1 - Virulence / avirulence formulas for the four *Pst* races used in the GWAS for seedling resistance. (PDF, 154 KB)
- Table S2 - Evaluation of different association models using Bayesian information criterion (BIC) implemented in GAPIT. (PDF, 126 KB)
- Table S3 - Pearson's correlation coefficients among the best linear unbiased estimates (BLUEs) of infection type (IT) and disease severity (SEV) response to *Pst* in single locations (MTV, PLM, and DVS) and combined locations (ALL) based on 875 spring wheat accessions from the NSGC. (PDF, 157 KB)
- Table S4 - Loci associated with significant differences in *Pst* resistance in at least three environments (one at P <0.01) in a collection of 875 spring wheat accessions. (PDF, 165 KB)
- Table S5 - Loci associated with significant differences in partial resistance to *Pst* in at least three environments (one at *P* <0.01) in a collection of 593 spring hexaploid wheat with infection type ≥ 3. (PDF, 163 KB)
- Table S6 - Frequencies across the seven subpopulations of favorable alleles for the QTL-tagging SNPs detected in the complete GWAS (875 accessions). (PDF, 132 KB)
- Table S7 - Frequencies across the seven subpopulations of the favorable alleles for SNPs detected in the GWAS for partial resistance (593 accessions). (PDF, 127 KB)
- Table S8 - ANOVA for *Pst* infection type (IT) and severity (SEV) based on 10 significant QTL and population structure (Q7) as covariable. (PDF, 158 KB)
- Table S9 - Pairwise fixation indexes (*Fst*) among populations based on 4,585 SNPs. (PDF, 138 KB)
- Table S10 - Number of 9K SNP mapped and 90K SNP projected into the confidence intervals of the 10 significant QTL described in Table 3. (PDF, 122 KB)
- Figure S1 - Plot of observed *vs.* expected cumulative *P* values using different GWAS models. (PDF, 225 KB)
- Figure S2 - Selected QTL for *Pst* infection type (IT) and disease severity (SEV) in a collection of 875 spring hexaploid wheat. (PDF, 142 KB)
- Figure S3 - Selected QTL for partial resistance to *Pst* in a collection of 593 accessions of spring wheat with IT≥3. (PDF, 171 KB)
- Figure S4 - Regression between (A) Infection type (IT) and (B) disease severity (SEV) and the number of favorable alleles in each of the 875 lines. (PDF, 202 KB)
- Figure S5 - Significant interactions among 10 selected QTLs (Table S8). (PDF, 217 KB)
- File S5 - Supplemental information for Figure 6. (PDF, 229 KB)
- File S1 - Genetic profiles of the 875 spring wheat accessions for the 97 QTL-tagging SNPs. Accessions are ordered by the number of favorable alleles. (.xlsx, 339 KB)
- File S2 - Results of BLASTX to *Brachypodium* and rice proteins using wheat transcripts corresponding to SNPs mapped to the confidence intervals of the 10 significant QTL as query (Table 3). Annotations were retrieved from the Phytozome database (http://www.phytozome.net/). (.xlsx, 9 MB)
- File S3 - Control resistance genes *Yr18/Lr34* and *Yr46/Lr47*. (.xlsx, 198 KB)
- File S4 - Integrated genetic map used to compare the relative map positions of *Pst* resistance genes and QTL detected in previous bi-parental population studies with the positions of the 10 GWAS QTL-tagging SNPs. (.xlsx, 5 MB)
