# Supplementary material for: A Genome-Wide Association Study of Resistance to Stripe Rust (Puccinia striiformis f. sp. tritici) in a Worldwide Collection of Hexaploid Spring Wheat (Triticum aestivum L.)
Source: G3 (Bethesda). 2015 Jan 20;5(3):449–65. doi: 10.1534/g3.114.014563 (PMC4349098; doi:10.1534/g3.114.014563)
Supplement: Supporting Information [file supp_g3.114.014563_014563SI.pdf]

G3 · Genes | Genomes | Genetics

A Genome-Wide Association Study of Resistance to Stripe Rust (*Puccinia striiformis* f. sp. *tritici*) in a Worldwide Collection of Hexaploid Spring Wheat (*Triticum aestivum* L.)

Marco Maccaferri[*,§1], Junli Zhang[*1], Peter Bulli[†1], Zewdie Abate[*], Shiaoman Chao[**], Dario Cantu[***], Eligio Bossolini[*], Xianming Chen[††], Michael Pumphrey[†], Jorge Dubcovsky[*,†††, 2]

[*] Department of Plant Sciences, University of California, Davis, CA 95616, USA

[§] Department of Agricultural Sciences (DipSA), University of Bologna, Bologna 40127, Italy

[†] Department of Crop and Soil Sciences, Washington State University, Pullman, WA 99164-6420, USA

[††] USDA-ARS, Wheat Genetics, Quality Physiology, and Disease Research Unit, and Department of Plant Pathology, Washington State University, Pullman, WA 99164, USA

[**] USDA-ARS, 1605 Albrecht Blvd, Fargo, ND 58105, USA

[***] Department of Viticulture and Enology, University of California, Davis, CA 95616, USA

[†††] Howard Hughes Medical Institute, Chevy Chase, MD 20815, USA

[1] The first three authors contributed equally to this study.

[2] Corresponding author: J. Dubcovsky. Email: jdubcovsky@ucdavis.edu Phone: (530) 752-5159

Supplemental Tables: 10

Supplemental Figures: 5

DOI: 10.1534/g3.114.014563

Table S1 Virulence / avirulence formulas for the four *Pst* races used in the GWAS for seedling resistance.

***Pst* race code**	**Virulence / avirulence formula**
PSTv-4	***Yr1, Yr6, Yr9, Yr17, Yr27, YrSP, YrTye***<br>/ *Yr5, Yr7, Yr8, Yr10, Yr15, Yr24, Yr32, Yr43, Yr44, YrTr1, YrExp2*
PSTv-14	***Yr1, Yr6, Yr7, Yr8, Yr9, Yr17, Yr27, Yr43, Yr44, YrTr1, YrExp2, YrTye***<br>/ *Yr5, Yr10, Yr15, Yr24, Yr32, YrSP*
PSTv-37	***Yr6, Yr7, Yr8, Yr9, Yr17, Yr27, Yr43, Yr44, YrTr1, YrExp2***<br>/ *Yr1, Yr5, Yr10, Yr15, Yr24, Yr32, YrTye, YrSP*
PSTv-40	***Yr6, Yr7, Yr8, Yr9, Yr10, Yr24, Yr27, Yr32, Yr43, Yr44, YrTr1, YrExp2***<br>/ *Yr1, Yr5, Yr10, Yr15, YrTye, YrSP*

Table S2 Evaluation of different association models using Bayesian information criterion (BIC) implemented in GAPIT.

General Linear Model (GLM)

Trait[a]	Naïve	Ward4	Ward7	Q4	Q7	PC10
IT_DVS_2011	-421	-414	-476	-414	-453	-442
IT_DVS_2012	-378	-367	-468	-384	-460	-429
IT_MTV_2012	-546	-548	-601	-549	-602	-593
IT_MTV_2013	-438	-443	-500	-436	-516	-503
IT_PLM_2011	-670	-664	-759	-657	-702	-679
IT_PLM_2012	-550	-537	-594	-543	-575	-571
SEV_DVS_2011	962	959	861	968	883	900
SEV_DVS_2012	1009	1015	913	1008	920	948
SEV_MTV_2012	769	768	694	770	689	698
SEV_MTV_2013	791	787	700	795	685	708
SEV_PLM_2011	643	651	556	664	610	633
SEV_PLM_2012	341	354	296	352	319	335

Mixed Linear Model (MLM)

Trait	K	K+Ward4	K+Ward7	K+Q4	K+Q7	K+PC10
IT_DVS_2011	**-575**	-559	-547	-558	-543	-519
IT_DVS_2012	**-555**	-536	-527	-535	-528	-502
IT_MTV_2012	**-748**	-729	-714	-730	-717	-691
IT_MTV_2013	**-645**	-625	-609	-626	-613	-590
IT_PLM_2011	**-865**	-846	-847	-849	-834	-804
IT_PLM_2012	**-714**	-695	-686	-694	-677	-657
SEV_DVS_2011	**787**	798	805	806	814	838
SEV_DVS_2012	**826**	844	850	845	852	880
SEV_MTV_2012	**540**	557	573	559	571	593
SEV_MTV_2013	**557**	576	589	577	586	609
SEV_PLM_2011	**451**	469	467	468	479	510
SEV_PLM_2012	**139**	155	167	159	175	198

Naïve: no population structure correction; **Q4** and **Q7**: general linear model (GLM) with STRUCTURE membership coefficients from the four main groups or the 7 subgroups as covariates; **Ward4** and **Ward7**: GLM with Ward cluster coefficients from the four main groups or the 7 subgroups as covariates; **PC10**: GLM with first 10 principal components as covariates; **K**: mixed linear model (MLM) with the 875 × 875 kinship matrix of identity-by-state (IBS). Genetic similarities were calculated using all 4585 SNPs. Then K was combined with the previous 5 models (**K+Q4, K+Q7, K+Ward4, K+Ward7, K+PC10**). The lowest BIC values (the best models) for each trait are indicated in bold. The BIC values for IT and SEV have opposite signs due to the transformation.

[a] IT = infection type; SEV = Disease severity; DVS = Davis, CA; MTV = Mount Vernon, WA; PLM = Pullman, WA.

Table S3 Pearson's correlation coefficients among the best linear unbiased estimates (BLUEs) of infection type (IT) and disease severity (SEV) response to *Pst* in single locations (MTV, PLM, and DVS)[a] and combined locations (ALL) [a] based on 875 spring wheat accessions from the NSGC. All correlation coefficients are highly significant (*P*< 0.0001).

BLUE [a]	IT_PLM	IT_DVS	IT_ALL	SEV_MTV	SEV_PLM	SEV_DVS	SEV_ALL
IT_MTV	0.77	0.73	0.92	0.92	0.76	0.74	0.88
IT_PLM		0.75	0.92	0.71	0.86	0.70	0.82
IT_DVS			0.90	0.71	0.73	0.90	0.86
IT_ALL				0.85	0.86	0.85	0.93
SEV_MTV					0.77	0.76	0.93
SEV_PLM						0.73	0.90
SEV_DVS							0.92
SEV_ALL							

[a] Locations: MTV = Mount Vernon, WA; PLM = Pullman, WA; DVS = Davis, CA; ALL = all environments.

Table S4 Loci associated with significant differences in *Pst* resistance in at least three environments (one at *P*<0.01) in a collection of 875 spring wheat accessions.

Chr.	Pos.[a]	QTL-representative SNP			Associated SNP[e]	PSTv races IT (-log *P*)[g]				*Pst* response (-log *P*)[g]	
	(cM)	Index[b]	Alleles[c]	Freq.[d]	Index IWA	4	14	37	40	IT-ALL[f]	SEV-ALL[f]
1A	39.0	IWA6441	T/C	0.56	-	-	-	-	-	1.5	**2.1**
1A	58.0	IWA5194	A/G	0.32	-	-	-	-	-	1.4	1.3
1A	59.6	IWA4061	A/G	0.63	4646, 4071	-	-	-	1.4	1.6	1.4
1A	84.2	IWA3475	T/C	0.58	5277, 7018, 7898	-	-	-	-	1.6	1.6
1A	88.1	IWA5174	A/C	0.39	6707, 6708	-	-	-	-	1.5	1.5
1A	120.3	IWA1225	T/G	0.69	3145	-	1.3	-	-	**2.0**	**2.8**
1A	148.1	IWA672	A/G	0.36	-	-	-	-	-	**2.0**	1.6
1A	158.6	IWA7893	T/C	0.83	1368	-	-	-	**2.0**	1.7	**3.2**
1A	173.7	IWA2035	A/G	0.63	1560, 693	-	-	-	-	1.6	**2.2**
1B	35.5	IWA962	A/G	0.14	4389	-	-	-	-	1.6	1.9
1B	51.9	IWA6758	A/G	0.31	189, 890, 6073	1.4	-	-	-	1.9	**2.0**
1B	57.6	IWA3307	T/C	0.49	-	-	-	-	-	1.8	1.6
1B	94.7	IWA3017	A/G	0.11	-	-	-	**4.0**	-	1.3	1.9
1B	109.3	IWA1825	T/C	0.35	5847, 1069, 3043	-	-	-	-	**2.0**	**2.4**
1B	123.4	IWA3892	A/G	0.70	846	-	-	-	1.3	**3.2**	**3.5**
1B	141.2	IWA2077	A/G	0.18	6647	1.9	-	-	-	**2.1**	**2.0**
1D	21.4	IWA2547	T/C	0.11	-	**3.0**	-	-	-	1.8	**2.2**
2A	9.9	**IWA422**	T/C	0.31	423, 3468, 3469	-	1.6	-	-	**3.9**	**3.2**
2A	62.2	IWA3520	A/C	0.52	-	-	-	-	-	1.4	1.8
2A	78.3	**IWA424**	T/C	0.70	-	-	1.9	-	**2.0**	1.5	**2.7**
2A	96.2	IWA5272	A/G	0.52	5273	-	-	-	-	**2.1**	**2.1**
2A	110.6	IWA7947	A/C	0.71	-	-	-	-	1.4	**2.2**	**2.2**
2A	160.2	IWA200	A/G	0.84	-	-	-	-	-	**2.1**	**2.0**
2B	112.3	IWA905	A/G	0.82	-	-	-	-	-	1.5	1.4
2B	147.3	IWA586	T/C	0.62	587, 4464	-	-	-	-	1.5	1.3
2B	163.4	IWA226	T/C	0.54	2294	1.4	1.9	-	**2.1**	1.7	**2.3**
2B	266.4	IWA3206	T/C	0.12	1668, 3206, 4619	**2.7**	-	-	**2.0**	1.9	1.6
3A	13.2	IWA5969	T/C	0.14	-	-	-	1.4	-	1.9	1.9
3A	27.5	IWA2049	T/C	0.14	2048, 7085, 2047, 7086	-	-	-	-	1.7	**2.1**
3A	35.0	IWA1996	A/G	0.87	885	1.3	1.5	-	-	1.9	1.6
3A	59.4	IWA5039	T/C	0.64	6877, 1308	-	-	-	**2.7**	1.9	1.8
3A	70.4	IWA8215	T/G	0.27	-	1.3	-	-	-	1.8	-
3A	75.2	IWA7011	A/G	0.24	-	-	1.5	-	-	1.8	1.3
3A	102.9	IWA2332	A/G	0.35	7440	-	-	-	-	1.8	**2.2**
3B	3.9	**IWA5202**	A/G	0.37	4796	-	-	-	-	**3.4**	1.3
3B	57.4	IWA6632	A/C	0.75	-	-	-	-	-	1.9	1.3
3B	73.8	IWA377	T/C	0.76	2622	-	-	-	-	1.4	-
3B	77.5	IWA8480	A/G	0.14	-	-	-	-	-	1.4	1.8
3B	84.5	IWA5890	T/C	0.53	-	-	-	-	-	1.7	**2.1**
3B	95.5	IWA6221	T/C	0.75	-	-	-	-	-	**2.4**	1.8
3B	101.4	IWA321	T/C	0.11	-	-	-	-	-	**2.0**	-
4A	35.2	IWA6100	A/G	0.33	4251	-	-	-	-	**2.4**	1.9

Table S4 Continuation

4A	44.0	IWA1992	A/<u>G</u>	0.58	-	-	-	1.4	-	1.3	**2.2**
4A	54.9	IWA7216	T/<u>C</u>	0.53	-	-	-	-	-	1.6	**2.0**
4A	68.1	IWA1570	T/<u>G</u>	0.17	5687, 3489, 3490, 5036,8, 7203	**2.4**	-	-	-	**3.2**	**3.0**
4A	167.3	IWA2170	A/<u>G</u>	0.37	7765, 1066, 6690	-	-	-	-	**2.4**	**2.3**
4A	181.7	IWA1034	T/<u>C</u>	0.17	-	-	-	-	-	**3.4**	**2.6**
4B	85.2	IWA6461	T/<u>C</u>	0.54	-	-	-	-	-	1.7	1.6
4D	26.9	**IWA5375**	<u>T</u>/G	0.10	5766	1.8	-	-	-	**3.0**	**3.9**
5A	4.9	IWA2144	<u>T</u>/C	0.19	2143, 2146	-	-	-	-	**2.2**	1.6
5A	119.3	IWA1486	<u>T</u>/C	0.24	4648	-	-	**2.0**	-	1.7	-
5A	189.2	**IWA6988**	<u>T</u>/C	0.16	-	-	1.7	-	-	**3.0**	**2.2**
5A	194.9	IWA2646	A/<u>G</u>	0.35	-	1.4	-	1.3	-	**2.6**	**2.3**
5B	0.0	IWA868	<u>T</u>/C	0.19	757	-	-	-	-	1.9	1.6
5B	68.3	IWA7227	T/<u>C</u>	0.31	-	-	**3.0**	1.5	-	**2.1**	1.7
5B	85.9	IWA3633	T/<u>C</u>	0.54	-	-	-	-	-	**2.8**	**2.8**
5B	119.9	IWA4280	<u>A</u>/G	0.10	8069, 7815	-	**<u>24.0</u>**	**<u>8.0</u>**	-	1.7	1.6
5B	205.8	IWA22	<u>A</u>/G	0.26	-	-	-	-	-	-	1.6
6A	75.5	IWA4824	<u>A</u>/G	0.44	-	1.4	-	**3.1**	1.4	1.8	1.8
6A	114.5	IWA6596	<u>T</u>/C	0.51	1671, 5074, 6508	-	-	-	-	1.5	**2.0**
6A	217.7	IWA3066	A/<u>G</u>	0.72	3203, 3204	1.4	-	-	-	**2.4**	**2.4**
6B	37.9	IWA8134	T/<u>C</u>	0.10	-	-	-	**2.3**	-	1.7	**2.5**
6B	38.5	IWA2888	T/<u>C</u>	0.65	1905, 1657	-	1.6	-	-	1.6	**2.4**
6B	50.8	IWA7625	<u>A</u>/G	0.44	2419, 1655, 2417, 2420, 4823, 4825, 4827	1.9	-	**3.1**	1.6	**2.3**	**2.3**
6B	84.5	IWA6770	A/<u>G</u>	0.52	3289, 6660	-	-	-	1.4	**2.5**	**2.6**
6B	112.3	**IWA7257**	<u>T</u>/G	0.23	-	-	-	-	-	**<u>5.0</u>**	**3.2**
6D	68.6	IWA7816	A/<u>G</u>	0.63	-	**2.3**	-	-	-	**2.2**	**2.1**
6D	73.2	**IWA167**	A/<u>C</u>	0.10	-	-	-	-	-	**<u>5.5</u>**	**4.5**
7A	6.2	IWA7306	<u>A</u>/G	0.53	-	1.4	-	-	-	1.7	**2.2**
7A	49.9	IWA7121	<u>T</u>/G	0.70	-	-	-	-	-	1.6	1.6
7A	105.5	IWA7549	<u>A</u>/C	0.83	-	-	-	-	-	**2.0**	**2.0**
7B	40.6	IWA1108	<u>T</u>/C	0.27	6143, 8233	-	-	-	-	1.9	1.7
7B	107.4	IWA615	T/<u>C</u>	0.18	-	-	1.8	-	1.3	**2.0**	1.7

[a] Scaled position from hexaploid wheat consensus map (Cavanagh et al. 2013).

[b] SNP indexes from Illumina iSelect 9K wheat assay (Cavanagh et al. 2013). **Bold**: QTL with experiment –wise Bonferroni *P*< 0.1

[c] SNP variant associated to the resistant response is underlined.

[d] Frequency of the favorable SNP variant.

[e] SNP loci in LD with the representative SNP and significantly associated to the *Pst* response (IWA).

[f] IT= infection type , SEV= disease severity. IT-ALL and SEV-ALL, best linear unbiased estimates (BLUEs) over all environments.

[g] Significances are reported as -log (*P* value): '-' = not-significant, 1.3 = *P*=0.05, values > 2 are in **bold**, and value ≥ 4 (experiment-wise significant at α = 0.10) are in **<u>bold underlined</u>**.

Table S5 Loci associated with significant differences in partial resistance to *Pst* in at least three environments (one at *P*<0.01) in a collection of 593 spring hexaploid wheat with infection type ≥ 3.

Chr.	Pos.[a]	QTL-representative SNP			Associated SNP[e]	PSTv races IT (-log *P*) [g]				*Pst* response [f] (-log *P*) [g]	
	(cM)	Index[b]	Alleles[c]	Freq.[d]	IWA index	4	14	37	40	IT-ALL	SEV-ALL
1A	28.2	IWA414	T/G	0.62	4644, 4506	-	-	-	-	**2.4**	**2.8**
1A	111.2	IWA577	A/C	0.46	4538, 1081, 5832, 578	-	-	-	-	**3.1**	**2.9**
1A	148.1	IWA672 *	A/G	0.36	-	-	-	-	-	-	-
1B	93.5	IWA5769	A/G	0.20	5862, 8246, 3017	-	-	**4.0**	-	**2.0**	1.7
1B	123.4	**IWA3892 ***	A/G	0.70	846 [h]	-	-	-	1.3	**3.1**	**4.2**
1D	49.3	**IWA980**	A/C	0.44	642	-	-	-	-	1.7	1.7
1D	91.5	IWA2341	T/C	0.37	2340	**2.6**	-	1.8	1.5	**2.0**	**2.2**
2A	9.9	**IWA422 ***	T/C	0.31	423, 3468, 3469	-	1.6	-	-	**3.1**	1.9
2A	177.7	IWA3576	T/C	0.18	-	-	-	-	-	1.5	-
2B	126.3	IWA4606	T/C	0.39	1102, 1127, 1128, 1129, 1130, 1131, 1177, 1188 [i]	-	-	-	-	**2.3**	1.3
2B	149.4	IWA7312	A/G	0.81	-	-	-	-	-	**2.1**	1.9
2B	197.2	IWA5177	T/C	0.81	7615, 2379, 4900	-	-	-	-	**2.9**	1.9
3A	59.4	IWA6877	T/C	0.11	5039, 1308	-	-	-	**2.7**	1.5	-
3B	3.9	**IWA5202 ***	A/G	0.37	4796, 4801	-	-	-	-	**4.1**	**-**
3B	57.4	IWA6632 *	A/C	0.75	-	-	-	-	-	**2.0**	1.4
3B	88.1	IWA4085	T/C	0.68	7519, 8196	-	-	-	-	**2.6**	**2.2**
4A	167.3	IWA2170 *	A/G	0.37	7765, 1066, 6690	-	-	-	-	1.4	1.8
4A	181.7	**IWA1034 ***	T/C	0.17	1034, 6696	-	-	-	-	**4.1**	**2.7**
4B	26.4	IWA8109	A/G	0.72	7311	1.3	-	-	-	**2.8**	**2.4**
4B	116.4	IWA27	A/G	0.62	3781	-	-	-	-	**2.2**	**2.1**
5B	0.0	IWA868 *	T/C	0.19	757	-	-	-	-	**2.6**	1.7
5B	85.9	IWA3633 *	T/C	0.54	-	-	-	-	-	**2.3**	**3.0**
5B	115.3	IWA8069	A/G	0.28	2335, 2336, 8187	-	**4.1**	1.6	-	**2.7**	1.5
6A	138.6	IWA3491	T/C	0.84	4950	-	-	-	-	**2.3**	1.4
6B	0.6	IWA1493	A/C	0.14	1492	-	-	-	-	1.6	-
6B	60.1	IWA2090	A/G	0.20	3869, 3167, 3634, 2244	-	-	-	-	1.6	1.4
6B	112.3	**IWA7257 ***	T/G	0.23	-	-	-	-	-	**2.5**	1.4
6B	118.5	IWA404	T/C	0.69	405	-	-	-	-	**2.0**	**2.2**
6D2	73.2	**IWA167 ***	A/C	0.10	7816					**3.8**	**3.9**
7A	56.4	IWA3715	T/C	0.62	7161, 1156	-	-	1.5	-	1.4	1.5
7A	106.2	IWA2011	A/G	0.69	2009, 4288, 6004	-	-	-	-	1.9	**2.0**
7A	145.3	IWA4483	A/G	0.57	-	-	-	-	-	1.5	1.4
7B	48.0	IWA8525	A/G	0.26	-	-	-	-	-	-	-

7B	65.6	IWA3437	A/G	0.59	306, 3438, 3691, 3812, 4250, 507, 6712	-	-	-	-	1.8	1.9
7B	99.0	IWA4701	T/C	0.65	1345, 1346, 4160, 4701	-	1.4	-	-	**2.5**	**2.2**

[a] Scaled position from the hexaploid wheat consensus map (Cavanagh et al. 2013)

[b] SNP indexes from the Illumina iSelect 9K wheat assay (Cavanagh et al. 2013). **Bold**: QTL with Bonferroni P<0.1.

* Eleven SNPs that are also identified using all 875 accessions.

[c] SNP variant associated to the resistant response is underlined.

[d] Frequency of the favorable SNP variant.

[e] SNP loci in LD with the representative SNP and significantly associated to the *Pst* response.

[f] IT= infection type, SEV= disease severity.IT-ALL and SEV-ALL, best linear unbiased estimates (BLUEs) over all environments.

[g] Significances are reported as -log (*P* value) as follows: '-' = not-significant, 1.3 = *P*=0.05, values > 2 are in **bold**, and value ≥ 4 (experiment-wise significant at α = 0.10) are in **bold underlined**.

[h] Additional associated SNPs: IWA1092, 919, 920, 7992, 8322, 3893, 846, 1791, 724, 7892, 6831

[i] Additional associated SNPs: IWA1102, 1127, 1128, 1129, 1130, 1131, 1177, 1188, 1229, 1237, 4604, 4606, 771, 776, 777, 829, 869, 874

Table S6 Frequencies across the seven subpopulations of favorable alleles for the QTL-tagging SNPs detected in the complete GWAS (875 accessions).

Chr.	Pos.[a] (cM)	QTL-representative SNP Index[b]	Alleles[c]	Freq.[d]	Pop. genetic structure (sub-groups by Ward clustering) 1A (134)	1B (84)	2 (169)	3 (285)	4A (90)	4B (69)	4C (44)
1A	39.0	IWA6441	T/C	0.56	0.62	0.73	0.56	0.59	0.58	0.49	0.37
1A	58.0	IWA5194	A/G	0.32	0.34	0.05	0.17	0.63	0.09	0.10	0.56
1A	59.6	IWA4061	A/G	0.63	0.83	0.96	0.84	0.34	0.51	0.84	0.71
1A	84.2	IWA3475	T/C	0.58	0.71	0.48	0.6	0.32	0.87	0.99	0.86
1A	88.1	IWA5174	A/C	0.39	0.59	0.64	0.33	0.51	0.11	0.03	1.00
1A	120.3	IWA1225	T/G	0.69	0.74	0.64	0.75	0.51	0.93	0.97	1.00
1A	148.1	IWA672	A/G	0.36	0.25	0.38	0.53	0.56	0.12	0.10	0.00
1A	158.6	IWA7893	T/C	0.83	0.68	0.77	0.95	0.94	0.83	0.59	0.93
1A	173.7	IWA2035	A/G	0.63	0.69	0.63	0.65	0.86	0.38	0.36	0.05
1B	35.5	IWA962	A/G	0.14	0.19	0.67	0.07	0.06	0.19	0.00	0.86
1B	51.9	IWA6758	A/G	0.31	0.36	0.55	0.35	0.26	0.15	0.46	0.95
1B	57.6	IWA3307	T/C	0.49	0.64	0.12	0.36	0.62	0.49	0.60	0.19
1B	94.7	IWA3017	A/G	0.11	0.23	0.06	0.04	0.14	0.12	0.01	0.23
1B	109.3	IWA1825	T/C	0.35	0.37	0.23	0.58	0.47	0.07	0.29	0.02
1B	123.4	**IWA3892**	A/G	0.70	0.77	0.87	0.70	0.76	0.53	0.77	0.70
1B	141.2	IWA2077	A/G	0.18	0.15	0.05	0.05	0.15	0.61	0.41	0.05
1D	21.4	IWA2547	T/C	0.11	0.19	0.05	0.10	0.16	0.02	0.10	0.12
2A	9.9	**IWA422**	T/C	0.31	0.35	0.10	0.19	0.25	0.56	0.77	0.93
2A	62.2	IWA3520	A/C	0.52	0.34	0.25	0.54	0.72	0.36	0.62	0.21
2A	78.3	**IWA424**	T/C	0.70	0.48	0.83	0.80	0.88	0.26	0.97	0.35
2A	96.2	IWA5272	A/G	0.52	0.51	0.68	0.33	0.78	0.07	0.86	0.00
2A	110.6	IWA7947	A/C	0.71	0.73	0.88	0.60	0.82	0.64	0.75	0.49
2A	160.2	IWA200	A/G	0.84	0.87	0.92	0.80	0.83	0.93	1.00	0.93
2B	112.3	IWA905	A/G	0.82	0.65	0.95	0.86	0.81	0.98	0.84	1.00
2B	147.3	IWA586	T/C	0.62	0.52	0.79	0.78	0.70	0.34	0.68	0.91
2B	163.4	IWA226	T/C	0.54	0.54	0.44	0.34	0.64	0.88	0.52	0.98
2B	266.4	IWA3206	T/C	0.12	0.26	0.31	0.18	0.06	0.01	0.04	0.00
3A	13.2	IWA5969	T/C	0.14	0.09	0.42	0.24	0.22	0.02	0.01	0.07
3A	27.5	IWA2049	T/C	0.14	0.35	0.20	0.14	0.07	0.15	0.01	0.88
3A	35.0	IWA1996	A/G	0.87	0.88	0.93	0.96	0.78	0.98	0.93	1.00
3A	59.4	IWA5039	T/C	0.64	0.52	0.81	0.85	0.79	0.24	0.83	0.19
3A	70.4	IWA8215	T/G	0.27	0.36	0.05	0.11	0.41	0.13	0.48	0.00
3A	75.2	IWA7011	A/G	0.24	0.15	0.04	0.18	0.31	0.13	0.79	0.02
3A	102.9	IWA2332	A/G	0.35	0.42	0.26	0.54	0.24	0.22	0.54	0.93
3B	3.9	**IWA5202**	A/G	0.37	0.41	0.52	0.47	0.40	0.09	0.32	0.12
3B	57.4	IWA6632	A/C	0.75	0.89	0.85	0.83	0.69	0.54	0.83	0.44
3B	73.8	IWA377	T/C	0.76	0.96	0.75	0.96	0.87	0.23	0.61	0.98
3B	77.5	IWA8480	A/G	0.14	0.13	0.12	0.09	0.18	0.15	0.10	0.98
3B	84.5	IWA5890	T/C	0.53	0.61	0.81	0.40	0.62	0.34	0.41	1.00
3B	95.5	IWA6221	T/C	0.75	0.91	0.67	0.85	0.81	0.49	0.64	1.00

3B	101.4	IWA321	T/C	0.11	0.29	0.27	0.16	0.02	0.00	0.00	0.00
4A	35.2	IWA6100	A/G	0.33	0.09	0.16	0.34	0.39	0.49	0.75	0.09
4A	44.0	IWA1992	A/G	0.58	0.56	0.25	0.42	0.68	0.60	0.78	0.93
4A	54.9	IWA7216	T/C	0.53	0.59	0.45	0.23	0.48	0.81	0.91	1.00
4A	68.1	IWA1570	T/G	0.17	0.55	0.06	0.10	0.10	0.16	0.01	1.00
4A	167.3	IWA2170	A/G	0.37	0.53	0.49	0.36	0.31	0.50	0.23	0.86
4A	181.7	**IWA1034**	T/C	0.17	0.08	0.04	0.03	0.17	0.39	0.28	0.00
4B	85.2	IWA6461	T/C	0.54	0.66	0.81	0.83	0.59	0.15	0.09	0.09
4D	26.9	**IWA5375**	T/G	0.10	0.38	0.12	0.05	0.02	0.10	0.01	0.88
5A	4.9	IWA2144	T/C	0.19	0.37	0.45	0.21	0.18	0.02	0.01	0.6
5A	119.3	IWA1486	T/C	0.24	0.28	0.19	0.28	0.27	0.08	0.67	0.28
5A	189.2	**IWA6988**	T/C	0.16	0.17	0.10	0.12	0.15	0.38	0.23	0.88
5A	194.9	IWA2646	A/G	0.35	0.35	0.31	0.62	0.39	0.27	0.33	0.14
5B	0.0	IWA868	T/C	0.19	0.05	0.00	0.11	0.23	0.43	0.35	0.02
5B	68.3	IWA7227	T/C	0.31	0.16	0.58	0.18	0.4	0.45	0.47	0.00
5B	85.9	IWA3633	T/C	0.54	0.69	0.83	0.57	0.61	0.07	0.03	0.00
5B	119.9	IWA4280	A/G	0.10	0.16	0.49	0.09	0.08	0.01	0.00	0.00
5B	205.8	IWA22	A/G	0.26	0.19	0.22	0.24	0.28	0.29	0.52	0.00
6A	75.5	IWA4824	A/G	0.44	0.21	0.1	0.71	0.38	0.78	0.42	0.98
6A	114.5	IWA6596	T/C	0.51	0.51	0.4	0.60	0.73	0.22	0.38	0.02
6A	217.7	IWA3066	A/G	0.72	0.84	0.63	0.91	0.70	0.73	0.48	0.91
6B	37.9	IWA8134	T/C	0.10	0.05	0.01	0.25	0.17	0.1	0.04	0.02
6B	38.5	IWA2888	T/C	0.65	0.87	0.45	0.78	0.61	0.82	0.58	0.98
6B	50.8	IWA7625	A/G	0.44	0.20	0.10	0.71	0.38	0.78	0.42	0.98
6B	84.5	IWA6770	A/G	0.52	0.18	0.54	0.38	0.70	0.76	0.28	0.79
6B	112.3	**IWA7257**	T/G	0.23	0.61	0.32	0.51	0.11	0.06	0.01	0.00
6D2	68.6	IWA7816	A/G	0.63	0.67	0.52	0.70	0.49	0.91	0.86	1.00
6D2	73.2	**IWA167**	A/C	0.10	0.36	0.10	0.03	0.06	0.05	0.01	0.95
7A	6.2	IWA7306	A/G	0.53	0.45	0.74	0.50	0.56	0.56	0.87	0.29
7A	49.9	IWA7121	T/G	0.70	0.68	0.83	0.51	0.76	0.64	0.99	0.88
7A	105.5	IWA7549	A/C	0.83	0.84	1.00	0.83	0.77	0.94	1.00	1.00
7B	40.6	IWA1108	T/C	0.27	0.22	0.42	0.25	0.33	0.17	0.35	0.16
7B	107.4	IWA615	T/C	0.18	0.18	0.18	0.10	0.27	0.20	0.03	1.00
N. of favorable alleles with freq. >0.90					2	5	4	1	6	8	26
N. of favorable alleles with freq. <0.10					5	10	9	8	15	17	21
Percent >0.9 + <0.10 by subpopulation					9.6	20.5	17.8	12.3	28.8	34.2	**64.4**
Average Genetic diversity (based on all SNPs)					0.39	0.34	0.34	0.38	0.27	0.26	**0.13**

[a] Scaled position from hexaploid wheat consensus map (Cavanagh et al. 2013).

[b] SNP indexes from Illumina iSelect 9K wheat assay (Cavanagh et al. 2013).

[c] SNP variant associated to the resistant response is underlined.

[d] Frequency of the favorable SNP variant.

Bold name: SNPs significant for Bonferroni P<0.10 (experiment-wise error rate), yellow: allele frequency < 0.1, green: allele frequency > 0.9.

Table S7 Frequencies across the seven subpopulations of the favorable alleles for SNPs detected in the GWAS for partial resistance (593 accessions). Only the 24 QTL that were not redundant with Table S6 are listed below.

Chr.	Pos.[a]	QTL-representative SNP			Pop. genetic structure (sub-groups by Ward clustering)						
	(cM)	Index[b]	Alleles[c]	Freq.[d]	**1A**	**1B**	**2**	**3**	**4A**	**4B**	**4C**
					134	84	169	285	90	69	44
1A	28.2	IWA414	T/G	0.62	0.72	0.94	0.73	0.50	0.48	0.63	0.95
1A	111.2	IWA577	A/C	0.46	0.47	0.29	0.50	0.28	0.67	0.78	1.00
1B	93.5	IWA5769	A/G	0.2	0.26	0.08	0.07	0.24	0.44	0.01	0.36
1D	49.3	**IWA980**	A/C	0.44	0.19	0.17	0.70	0.60	0.34	0.43	0.20
1D	91.5	IWA2341	T/C	0.37	0.69	0.22	0.63	0.28	0.11	0.29	0.00
2A	177.7	IWA3576	T/C	0.18	0.12	0.02	0.19	0.02	0.41	0.62	0.11
2B	126.3	IWA4606	T/C	0.39	0.49	0.66	0.43	0.43	0.07	0.49	0.02
2B	149.4	IWA7312	A/G	0.81	0.55	0.86	0.82	0.86	0.94	1.00	0.91
2B	197.2	IWA5177	T/C	0.81	0.92	0.98	0.82	0.90	0.60	0.71	0.09
3A	59.4	IWA6877	T/C	0.11	0.15	0.60	0.07	0.14	0.00	0.00	0.00
3B	88.1	IWA4085	T/C	0.68	0.54	0.80	0.72	0.69	0.79	0.43	1.00
4B	26.4	IWA8109	A/G	0.72	0.75	0.88	0.73	0.75	0.53	0.62	0.98
4B	116.4	IWA27	A/G	0.62	0.79	0.82	0.81	0.56	0.15	0.59	1.00
5B	115.3	IWA8069	A/G	0.28	0.25	0.51	0.33	0.30	0.06	0.46	0.02
6A	138.6	IWA3491	T/C	0.84	0.81	0.60	0.95	0.87	0.67	0.97	0.77
6B	0.6	IWA1493	A/C	0.14	0.35	0.15	0.18	0.11	0.00	0.00	0.00
6B	60.1	IWA2090	A/G	0.2	0.60	0.40	0.07	0.09	0.00	0.00	0.00
6B	118.5	IWA404	T/C	0.69	0.92	0.81	0.79	0.54	0.70	0.71	1.00
7A	56.4	IWA3715	T/C	0.62	0.59	0.63	0.44	0.78	0.69	0.58	0.89
7A	106.2	IWA2011	A/G	0.69	0.78	0.79	0.55	0.65	0.92	0.54	0.98
7A	145.3	IWA4483	A/G	0.57	0.82	0.69	0.69	0.46	0.56	0.19	0.86
7B	48.0	IWA8525	A/G	0.26	0.23	0.14	0.14	0.29	0.50	0.17	0.86
7B	65.6	IWA3437	A/G	0.59	0.80	0.75	0.40	0.70	0.44	0.19	0.98
7B	99.0	IWA4701	T/C	0.65	0.70	0.79	0.69	0.53	0.68	0.68	0.23
	N. of favorable alleles with freq. >0.90				2	2	1	0	2	2	8
	N. of favorable alleles with freq. <0.10				0	2	3	1	5	4	7
	Percent >0.9 + <0.10 by subpopulation				8.3	16.7	16.7	4.2	29.2	25.0	**62.5**

[a] Scaled position from hexaploid wheat consensus map (Cavanagh et al. 2013).

[b] SNP indexes from Illumina iSelect 9K wheat assay (Cavanagh et al. 2013).

[c] SNP variant associated to the resistant response is underlined.

[d] Frequency of the favorable SNP variant.

Bold name: SNPs significant for Bonferroni P<0.10 (experiment-wise error rate), yellow: allele frequency < 0.1, green: allele frequency > 0.9.

Table S8 ANOVA for *Pst* infection type (IT) and severity (SEV) based on 10 significant QTL and population structure (Q7) as covariable. P values are presented for the structure alone, the structure and the 10 QTL, and the structure plus the 10 QTL plus the significant interactions.

	Structure alone		Structure & 10 QTL		Structure & 10 QTL & 6 int.	
Source	**IT**	**SEV**	**IT**	**SEV**	**IT**	**SEV**
Q1	0.0086	0.0011	0.3547	0.3673	0.7763	0.7429
Q2	0.0544	0.0386	0.0019	0.0034	0.0004	0.0008
Q3	<.0001	<.0001	0.7441	0.6287	0.234	0.6761
Q4	<.0001	<.0001	0.6426	0.8195	0.2053	0.2891
Q5	0.0898	0.0395	0.3596	0.4114	0.1244	0.1339
Q6	<.0001	<.0001	0.0001	<.0001	0.0014	<.0001
IWA980			0.0001	<.0001	0.0096	0.0092
IWA3892			0.0001	0.0001	0.0001	<.0001
IWA1034			0.0020	0.0083	0.0003	0.0003
IWA422			<.0001	<.0001	0.1813	0.2471
IWA424			0.1756	0.0429	0.6596	0.4986
IWA5202			<.0001	0.0526	0.3835	0.6191
IWA5375			0.0005	<.0001	0.0024	0.0005
IWA6988			0.0008	0.0072	0.0002	0.0017
IWA7257			<.0001	0.0004	0.0102	0.1154
IWA167			<.000	<.0001	<.0001	<.0001
IWA1034*IWA424					0.0419	0.0023
IWA980*IWA5202					0.0447	0.0877
IWA422*IWA6988					<.0001	<.0001
IWA422*IWA7257					0.0011	0.0034
IWA5202*IWA167					0.0259	0.0191
IWA6988*IWA167					0.0047	0.0087
R^2	0.141	0.167	0.290	0.285	0.330	0.324

Table S9 Pairwise fixation indexes (*Fst*) among populations based on 4,585 SNPs. All comparisons are significant at $P < 0.001$ with 1000 permutations.

Subgroup	1A	1B	2	3	4A	4B	4C
1A	0.000						
1B	0.105	0.000					
2	0.109	0.127	0.000				
3	0.106	0.117	0.087	0.000			
4A	0.213	0.276	0.255	0.225	0.000		
4B	0.212	0.263	0.202	0.184	0.200	0.000	
4C	0.304	0.427	0.402	0.360	0.296	0.447	0.000

Table S10 Number of 9K SNP mapped and 90K SNP projected into the confidence intervals of the 10 significant QTL described in Table 3. A tentative annotation for the wheat contigs including these SNPs was inferred from annotations in the Phytozome database based on the best hits in rice and *Brachypodium* protein databases. Detailed information for each SNP is provided in supplemental **File S2**. Plant resistance proteins (*R* genes) include CC-NB-LRR (coiled-coil domain, nucleotide binding site, leucine-rich repeat), RLP (receptor like proteins coupled with extracellular LRR) and RLK (kinase domain coupled with LRR).

Marker	Chr.	Position	No. SNP in QTL CI			Functional annotation	
		cM	9k-anchor SNP	Additional 90k-projected SNP	Total	No. of annotated orthologs	No. of *R* genes
IWA3892	1B	123.4	8	41	49	26	5
IWA980	1D	49.3	14	110	124	68	5
IWA422	2A	9.9	24	194	218	140	35
IWA424	2A	78.3	27	201	218	146	3
IWA5202	3B	3.9	18	88	106	74	16
IWA1034	4A[a]	181.7	12	11	23	19	10
IWA5375	4D	26.9	7	49	56	47	1
IWA6988	5A[b]	189.2	3	21	24	15	0
IWA7257	6B	112.3	4	24	28	19	0
IWA167	6D	73.2	8	64	76	54	2
**Total**			119	803	922	608	77

[a] *IWA1034* is in the region of 4AL translocated from 7BS (homology 7AS/4AL/7DS).
[b] *IWA6988* is in the region of 5AL translocated from 4AL (homology 5AL/4BL/4DL).

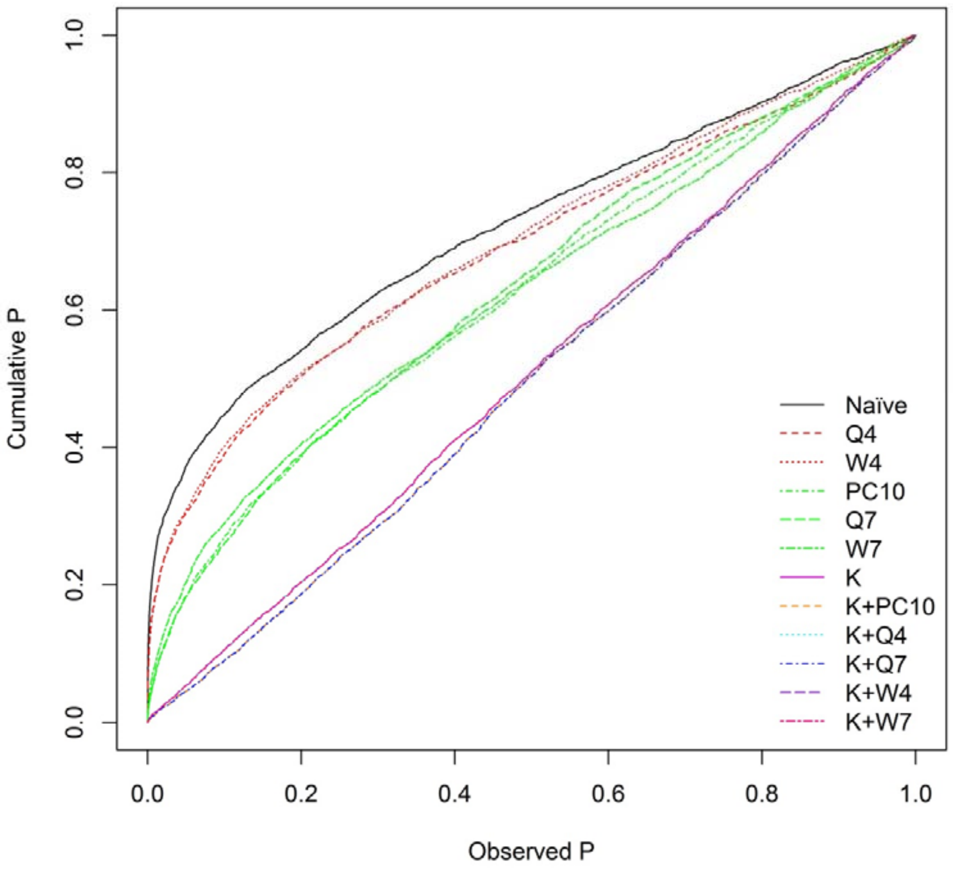

Figure S1 Plot of observed *vs.* expected cumulative *P* values using different GWAS models. The compared models are based on the IT data from UC Davis, 2011. **Naïve**: no population structure correction; **Q4 and Q7**: general linear model (GLM) with STRUCTURE membership coefficients from the four main groups or the 7 subgroups as covariates; **Ward 4 and Ward 7**: GLM with Ward cluster coefficients from the four main groups or the 7 subgroups as covariates; **PC10**: GLM with first 10 principal components as covariates; **K**: mixed linear model (MLM) with the 875 × 875 kinship matrix of identity-by-state (IBS). **K** was then combined with the previous 5 models (**K+Q4, K+Q7, K+W4, K+W7, K+PC10**). Genetic similarities were calculated using all 4,585 SNPs.

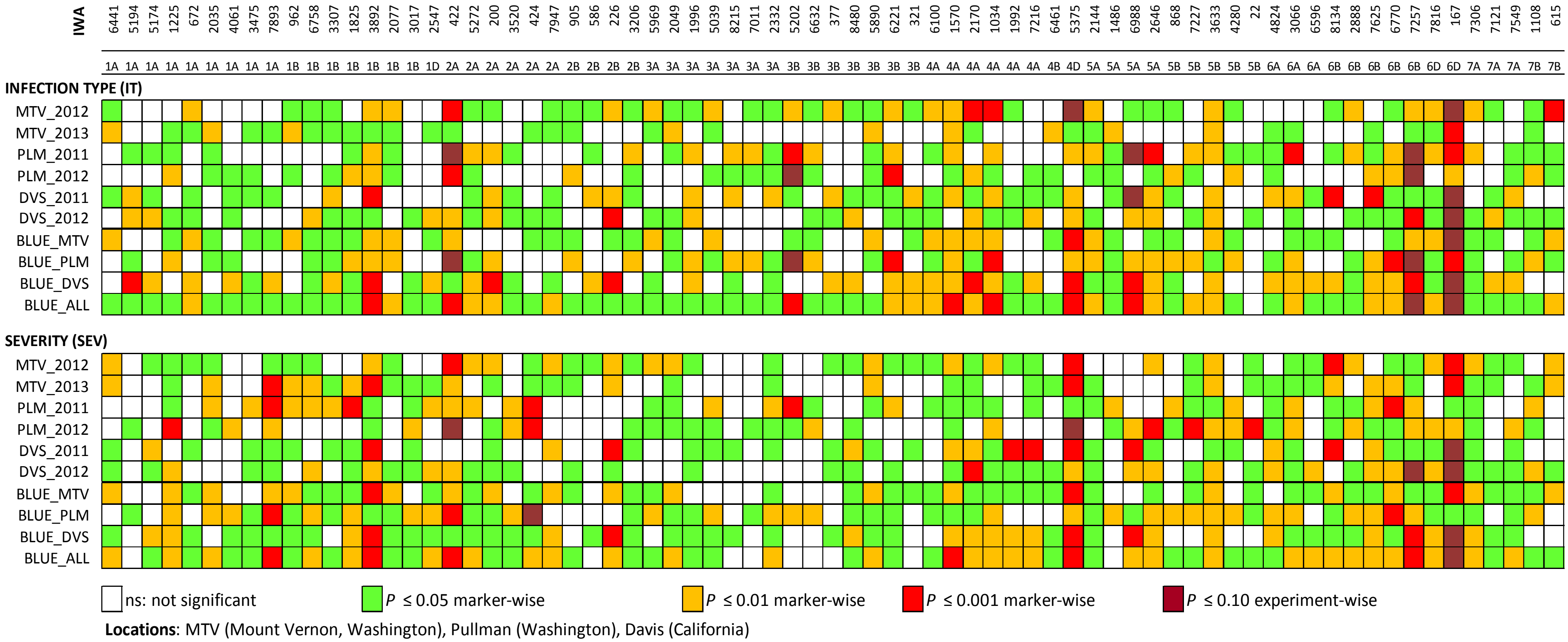

Figure S2 Selected QTL for *Pst* infection type (IT) and disease severity (SEV) in a collection of 875 spring hexaploid wheat. Only QTL significant in at least 3 environments (with at least one with *P*<0.01) are reported. Best Linear Unbiased Estimates (BLUEs) are calculated for each location separately and for the 6 combined environments (BLUE_ALL).

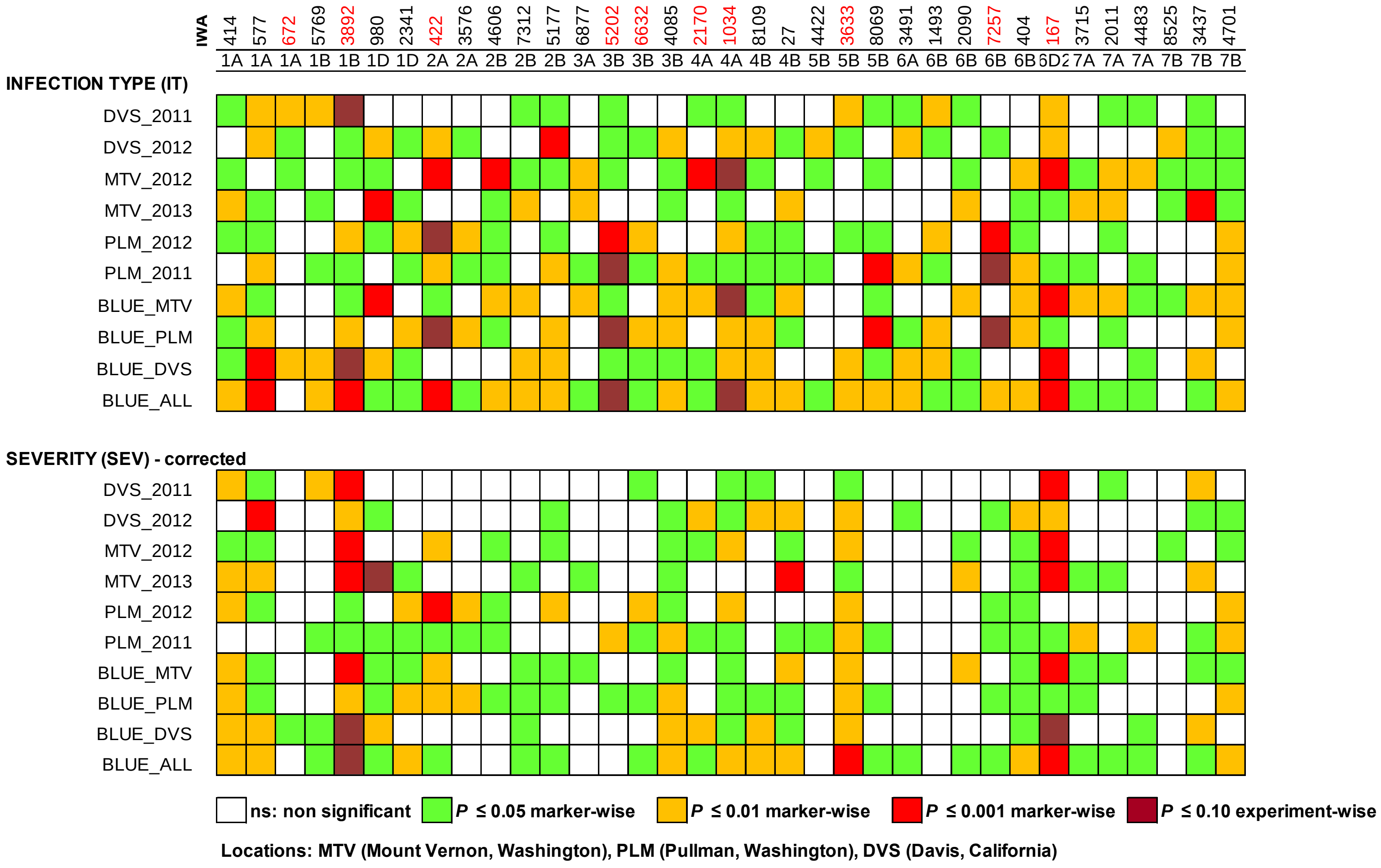

Figure S3 Selected QTL for partial resistance to *Pst* in a collection of 593 accessions of spring wheat with IT≥3. Only QTL significant in at least 3 environments (with at least one of them with *P*<0.01) are reported. SNP names in red are also significant in the complete analysis in **Figure S2**. Best linear unbiased estimates (BLUEs) are calculated for each location and for the 6 combined environments (BLUE_ALL).

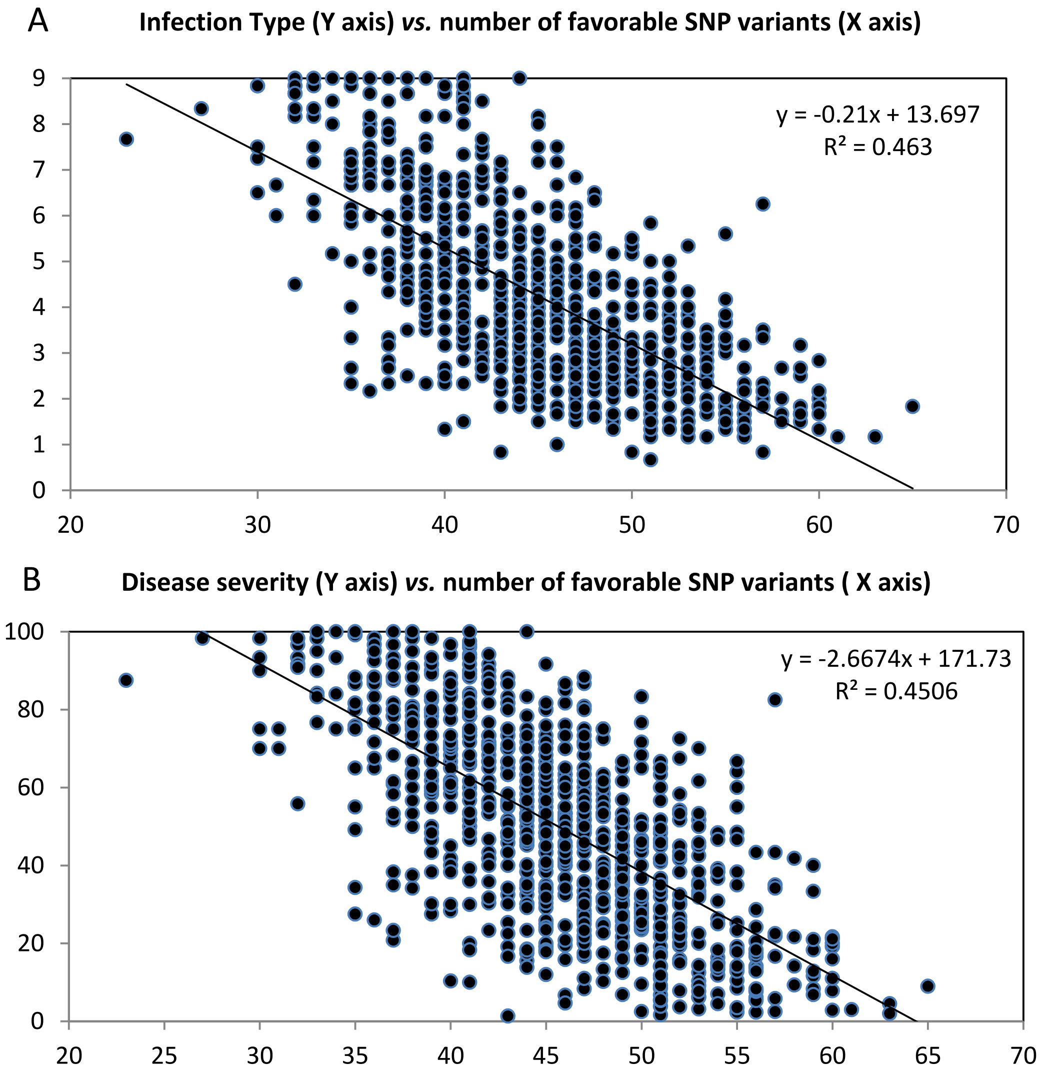

Figure S4 Regression between **(A)** Infection type (IT) and **(B)** disease severity (SEV) and the number of favorable alleles in each of the 875 lines. Both regressions were highly significant $P<0.0001$. Original data is available in **File S1**.

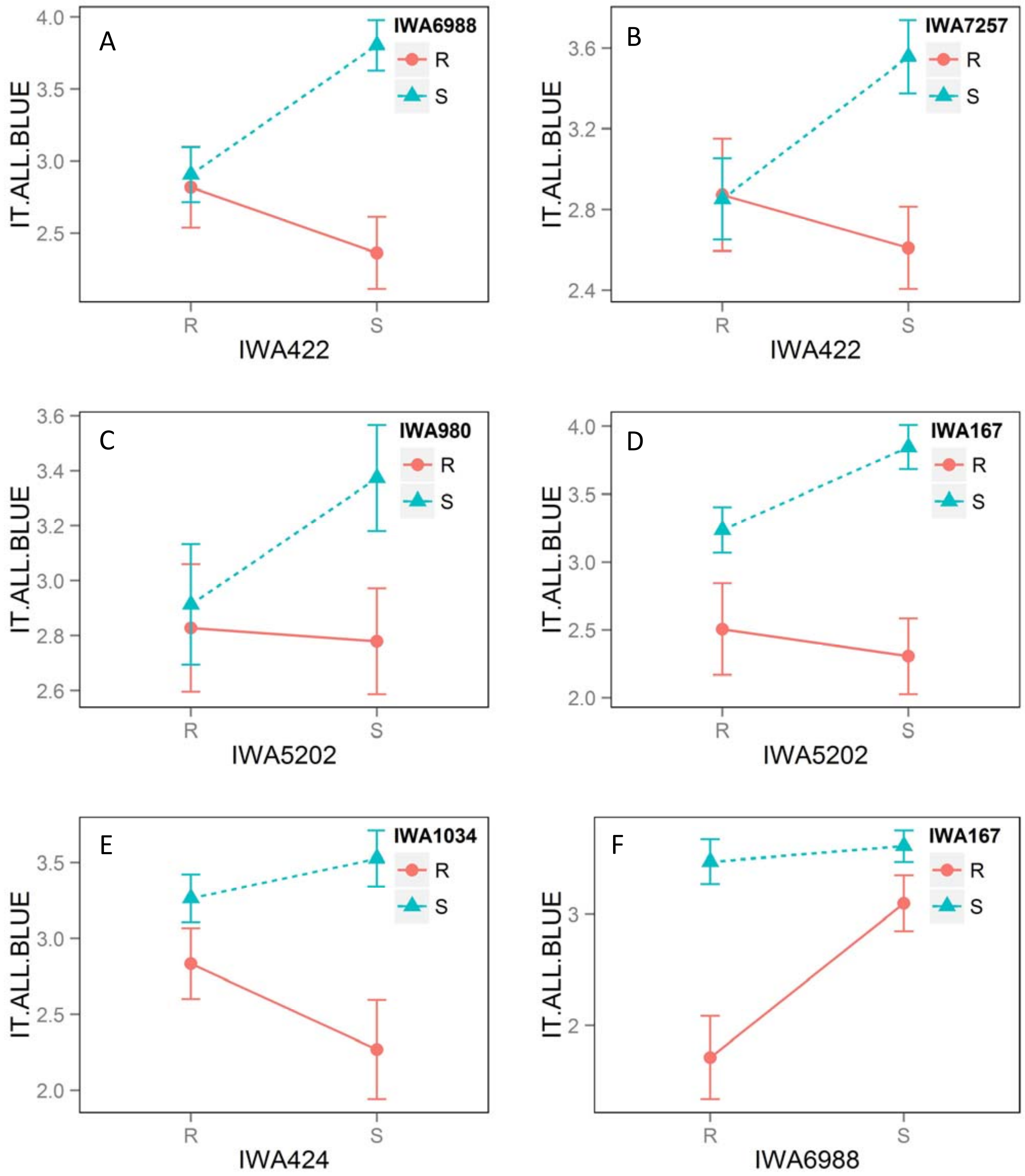

Figure S5 Significant interactions among 10 selected QTLs (**Table S8**). IT.ALL.BLUE values are the least square means from the full model ANOVA for BLUE values across all locations (**Table S8**) ± the standard error of the least square means.

Files S1-S5

Available for download at http://www.g3journal.org/lookup/suppl/doi:10.1534/g3.114.014563/-/DC1

File S1 Genetic profiles of the 875 spring wheat accessions for the 97 QTL-tagging SNPs. Accessions are ordered by the number of favorable alleles.

File S2 Results of BLASTX to *Brachypodium* and rice proteins using wheat transcripts corresponding to SNPs mapped to the confidence intervals of the 10 significant QTL as query (Table 3). Annotations were retrieved from the Phytozome database (http://www.phytozome.net/).

File S3 Control resistance genes *Yr18/Lr34* and *Yr46/Lr47*.

File S4 Integrated genetic map used to compare the relative map positions of *Pst* resistance genes and QTL detected in previous bi-parental population studies with the positions of the 10 GWAS QTL-tagging SNPs.

File S5 Supplemental information for **Figure 6**.
