# Supplementary material for: A Genome-Wide Association Study of Resistance to Stripe Rust (Puccinia striiformis f. sp. tritici) in a Worldwide Collection of Hexaploid Spring Wheat (Triticum aestivum L.)
Source: G3 (Bethesda). 2015 Jan 20;5(3):449–65. doi: 10.1534/g3.114.014563 (PMC4349098; doi:10.1534/g3.114.014563)
Supplement: Supporting Information [file supp_g3.114.014563_TableS1.pdf]

Table S1 Virulence / avirulence formulas for the four *Pst* races used in the GWAS for seedling resistance.

***Pst* race code**	**Virulence / avirulence formula**
PSTv-4	***Yr1, Yr6, Yr9, Yr17, Yr27, YrSP, YrTye*** / *Yr5, Yr7, Yr8, Yr10, Yr15, Yr24, Yr32, Yr43, Yr44, YrTr1, YrExp2*
PSTv-14	***Yr1, Yr6, Yr7, Yr8, Yr9, Yr17, Yr27, Yr43, Yr44, YrTr1, YrExp2, YrTye*** / *Yr5, Yr10, Yr15, Yr24, Yr32, YrSP*
PSTv-37	***Yr6, Yr7, Yr8, Yr9, Yr17, Yr27, Yr43, Yr44, YrTr1, YrExp2*** / *Yr1, Yr5, Yr10, Yr15, Yr24, Yr32, YrTye, YrSP*
PSTv-40	***Yr6, Yr7, Yr8, Yr9, Yr10, Yr24, Yr27, Yr32, Yr43, Yr44, YrTr1, YrExp2*** / *Yr1, Yr5, Yr10, Yr15, YrTye, YrSP*
