# Supplementary material for: A Genome-Wide Association Study of Resistance to Stripe Rust (Puccinia striiformis f. sp. tritici) in a Worldwide Collection of Hexaploid Spring Wheat (Triticum aestivum L.)
Source: G3 (Bethesda). 2015 Jan 20;5(3):449–65. doi: 10.1534/g3.114.014563 (PMC4349098; doi:10.1534/g3.114.014563)
Supplement: Supporting Information [file supp_g3.114.014563_TableS2.pdf]

Table S2 Evaluation of different association models using Bayesian information criterion (BIC) implemented in GAPIT.

General Linear Model (GLM)

Trait[a]	Naïve	Ward4	Ward7	Q4	Q7	PC10
IT_DVS_2011	-421	-414	-476	-414	-453	-442
IT_DVS_2012	-378	-367	-468	-384	-460	-429
IT_MTV_2012	-546	-548	-601	-549	-602	-593
IT_MTV_2013	-438	-443	-500	-436	-516	-503
IT_PLM_2011	-670	-664	-759	-657	-702	-679
IT_PLM_2012	-550	-537	-594	-543	-575	-571
SEV_DVS_2011	962	959	861	968	883	900
SEV_DVS_2012	1009	1015	913	1008	920	948
SEV_MTV_2012	769	768	694	770	689	698
SEV_MTV_2013	791	787	700	795	685	708
SEV_PLM_2011	643	651	556	664	610	633
SEV_PLM_2012	341	354	296	352	319	335

Mixed Linear Model (MLM)

Trait	K	K+Ward4	K+Ward7	K+Q4	K+Q7	K+PC10
IT_DVS_2011	**-575**	-559	-547	-558	-543	-519
IT_DVS_2012	**-555**	-536	-527	-535	-528	-502
IT_MTV_2012	**-748**	-729	-714	-730	-717	-691
IT_MTV_2013	**-645**	-625	-609	-626	-613	-590
IT_PLM_2011	**-865**	-846	-847	-849	-834	-804
IT_PLM_2012	**-714**	-695	-686	-694	-677	-657
SEV_DVS_2011	**787**	798	805	806	814	838
SEV_DVS_2012	**826**	844	850	845	852	880
SEV_MTV_2012	**540**	557	573	559	571	593
SEV_MTV_2013	**557**	576	589	577	586	609
SEV_PLM_2011	**451**	469	467	468	479	510
SEV_PLM_2012	**139**	155	167	159	175	198

Naïve: no population structure correction; **Q4** and **Q7**: general linear model (GLM) with STRUCTURE membership coefficients from the four main groups or the 7 subgroups as covariates; **Ward4** and **Ward7**: GLM with Ward cluster coefficients from the four main groups or the 7 subgroups as covariates; **PC10**: GLM with first 10 principal components as covariates; **K**: mixed linear model (MLM) with the 875 × 875 kinship matrix of identity-by-state (IBS). Genetic similarities were calculated using all 4585 SNPs. Then K was combined with the previous 5 models (**K+Q4, K+Q7, K+Ward4, K+Ward7, K+PC10**). The lowest BIC values (the best models) for each trait are indicated in bold. The BIC values for IT and SEV have opposite signs due to the transformation.

[a] IT = infection type; SEV = Disease severity; DVS = Davis, CA; MTV = Mount Vernon, WA; PLM = Pullman, WA.
