# Supplementary material for: A Genome-Wide Association Study of Resistance to Stripe Rust (Puccinia striiformis f. sp. tritici) in a Worldwide Collection of Hexaploid Spring Wheat (Triticum aestivum L.)
Source: G3 (Bethesda). 2015 Jan 20;5(3):449–65. doi: 10.1534/g3.114.014563 (PMC4349098; doi:10.1534/g3.114.014563)
Supplement: Supporting Information [file supp_g3.114.014563_TableS3.pdf]

Table S3 Pearson's correlation coefficients among the best linear unbiased estimates (BLUEs) of infection type (IT) and disease severity (SEV) response to *Pst* in single locations (MTV, PLM, and DVS)[a] and combined locations (ALL) [a] based on 875 spring wheat accessions from the NSGC. All correlation coefficients are highly significant (*P*< 0.0001).

BLUE [a]	IT_PLM	IT_DVS	IT_ALL	SEV_MTV	SEV_PLM	SEV_DVS	SEV_ALL
IT_MTV	0.77	0.73	0.92	0.92	0.76	0.74	0.88
IT_PLM		0.75	0.92	0.71	0.86	0.70	0.82
IT_DVS			0.90	0.71	0.73	0.90	0.86
IT_ALL				0.85	0.86	0.85	0.93
SEV_MTV					0.77	0.76	0.93
SEV_PLM						0.73	0.90
SEV_DVS							0.92
SEV_ALL							

[a] Locations: MTV = Mount Vernon, WA; PLM = Pullman, WA; DVS = Davis, CA; ALL = all environments.
