# Supplementary material for: A Genome-Wide Association Study of Resistance to Stripe Rust (Puccinia striiformis f. sp. tritici) in a Worldwide Collection of Hexaploid Spring Wheat (Triticum aestivum L.)
Source: G3 (Bethesda). 2015 Jan 20;5(3):449–65. doi: 10.1534/g3.114.014563 (PMC4349098; doi:10.1534/g3.114.014563)
Supplement: Supporting Information [file supp_g3.114.014563_TableS4.pdf]

**Table S4 Loci associated with significant differences in *Pst* resistance in at least three environments (one at $P<0.01$) in a collection of 875 spring wheat accessions.**

| Chr. | Pos.[a] | QTL-representative SNP | | | Associated SNP[e] | PSTv races IT (-log *P*)[g] | | | | *Pst* response (-log *P*)[g] | |
|---|---|---|---|---|---|---|---|---|---|---|---|
| | (cM) | Index[b] | Alleles[c] | Freq.[d] | Index IWA | 4 | 14 | 37 | 40 | IT-ALL[f] | SEV-ALL[f] |
| 1A | 39.0 | IWA6441 | T/C | 0.56 | - | - | - | - | - | 1.5 | **2.1** |
| 1A | 58.0 | IWA5194 | A/G | 0.32 | - | - | - | - | - | 1.4 | 1.3 |
| 1A | 59.6 | IWA4061 | A/G | 0.63 | 4646, 4071 | - | - | - | 1.4 | 1.6 | 1.4 |
| 1A | 84.2 | IWA3475 | T/C | 0.58 | 5277, 7018, 7898 | - | - | - | - | 1.6 | 1.6 |
| 1A | 88.1 | IWA5174 | A/C | 0.39 | 6707, 6708 | - | - | - | - | 1.5 | 1.5 |
| 1A | 120.3 | IWA1225 | T/G | 0.69 | 3145 | - | 1.3 | - | - | **2.0** | **2.8** |
| 1A | 148.1 | IWA672 | A/G | 0.36 | - | - | - | - | - | **2.0** | 1.6 |
| 1A | 158.6 | IWA7893 | T/C | 0.83 | 1368 | - | - | - | **2.0** | 1.7 | **3.2** |
| 1A | 173.7 | IWA2035 | A/G | 0.63 | 1560, 693 | - | - | - | - | 1.6 | **2.2** |
| 1B | 35.5 | IWA962 | A/G | 0.14 | 4389 | - | - | - | - | 1.6 | 1.9 |
| 1B | 51.9 | IWA6758 | A/G | 0.31 | 189, 890, 6073 | 1.4 | - | - | - | 1.9 | **2.0** |
| 1B | 57.6 | IWA3307 | T/C | 0.49 | - | - | - | - | - | 1.8 | 1.6 |
| 1B | 94.7 | IWA3017 | A/G | 0.11 | - | - | - | **4.0** | - | 1.3 | 1.9 |
| 1B | 109.3 | IWA1825 | T/C | 0.35 | 5847, 1069, 3043 | - | - | - | - | **2.0** | **2.4** |
| 1B | 123.4 | IWA3892 | A/G | 0.70 | 846 | - | - | - | 1.3 | **3.2** | **3.5** |
| 1B | 141.2 | IWA2077 | A/G | 0.18 | 6647 | 1.9 | - | - | - | **2.1** | **2.0** |
| 1D | 21.4 | IWA2547 | T/C | 0.11 | - | **3.0** | - | - | - | 1.8 | **2.2** |
| 2A | 9.9 | **IWA422** | T/C | 0.31 | 423, 3468, 3469 | - | 1.6 | - | - | **3.9** | **3.2** |
| 2A | 62.2 | IWA3520 | A/C | 0.52 | - | - | - | - | - | 1.4 | 1.8 |
| 2A | 78.3 | **IWA424** | T/C | 0.70 | - | - | 1.9 | - | **2.0** | 1.5 | **2.7** |
| 2A | 96.2 | IWA5272 | A/G | 0.52 | 5273 | - | - | - | - | **2.1** | **2.1** |
| 2A | 110.6 | IWA7947 | A/C | 0.71 | - | - | - | - | 1.4 | **2.2** | **2.2** |
| 2A | 160.2 | IWA200 | A/G | 0.84 | - | - | - | - | - | **2.1** | **2.0** |
| 2B | 112.3 | IWA905 | A/G | 0.82 | - | - | - | - | - | 1.5 | 1.4 |
| 2B | 147.3 | IWA586 | T/C | 0.62 | 587, 4464 | - | - | - | - | 1.5 | 1.3 |
| 2B | 163.4 | IWA226 | T/C | 0.54 | 2294 | 1.4 | 1.9 | - | **2.1** | 1.7 | **2.3** |
| 2B | 266.4 | IWA3206 | T/C | 0.12 | 1668, 3206, 4619 | **2.7** | - | - | **2.0** | 1.9 | 1.6 |
| 3A | 13.2 | IWA5969 | T/C | 0.14 | - | - | - | 1.4 | - | 1.9 | 1.9 |
| 3A | 27.5 | IWA2049 | T/C | 0.14 | 2048, 7085, 2047, 7086 | - | - | - | - | 1.7 | **2.1** |
| 3A | 35.0 | IWA1996 | A/G | 0.87 | 885 | 1.3 | 1.5 | - | - | 1.9 | 1.6 |
| 3A | 59.4 | IWA5039 | T/C | 0.64 | 6877, 1308 | - | - | - | **2.7** | 1.9 | 1.8 |
| 3A | 70.4 | IWA8215 | T/G | 0.27 | - | 1.3 | - | - | - | 1.8 | - |
| 3A | 75.2 | IWA7011 | A/G | 0.24 | - | - | 1.5 | - | - | 1.8 | 1.3 |
| 3A | 102.9 | IWA2332 | A/G | 0.35 | 7440 | - | - | - | - | 1.8 | **2.2** |
| 3B | 3.9 | **IWA5202** | A/G | 0.37 | 4796 | - | - | - | - | **3.4** | 1.3 |
| 3B | 57.4 | IWA6632 | A/C | 0.75 | - | - | - | - | - | 1.9 | 1.3 |
| 3B | 73.8 | IWA377 | T/C | 0.76 | 2622 | - | - | - | - | 1.4 | - |
| 3B | 77.5 | IWA8480 | A/G | 0.14 | - | - | - | - | - | 1.4 | 1.8 |
| 3B | 84.5 | IWA5890 | T/C | 0.53 | - | - | - | - | - | 1.7 | **2.1** |
| 3B | 95.5 | IWA6221 | T/C | 0.75 | - | - | - | - | - | **2.4** | 1.8 |
| 3B | 101.4 | IWA321 | T/C | 0.11 | - | - | - | - | - | **2.0** | - |
| 4A | 35.2 | IWA6100 | A/G | 0.33 | 4251 | - | - | - | - | **2.4** | 1.9 |

**Table S4** Continuation

| | | | | | | | | | | | |
|---|---|---|---|---|---|---|---|---|---|---|---|
| 4A | 44.0 | IWA1992 | A/G | 0.58 | - | - | - | 1.4 | - | 1.3 | **2.2** |
| 4A | 54.9 | IWA7216 | T/C | 0.53 | - | - | - | - | - | 1.6 | **2.0** |
| 4A | 68.1 | IWA1570 | T/G | 0.17 | 5687, 3489, 3490, 5036,8, 7203 | **2.4** | - | - | - | **3.2** | **3.0** |
| 4A | 167.3 | IWA2170 | A/G | 0.37 | 7765, 1066, 6690 | - | - | - | - | **2.4** | **2.3** |
| 4A | 181.7 | IWA1034 | T/C | 0.17 | - | - | - | - | - | **3.4** | **2.6** |
| 4B | 85.2 | IWA6461 | T/C | 0.54 | - | - | - | - | - | 1.7 | 1.6 |
| 4D | 26.9 | **IWA5375** | T/G | 0.10 | 5766 | 1.8 | - | - | - | **3.0** | **3.9** |
| 5A | 4.9 | IWA2144 | T/C | 0.19 | 2143, 2146 | - | - | - | - | **2.2** | 1.6 |
| 5A | 119.3 | IWA1486 | T/C | 0.24 | 4648 | - | - | **2.0** | - | 1.7 | - |
| 5A | 189.2 | **IWA6988** | T/C | 0.16 | - | - | 1.7 | - | - | **3.0** | **2.2** |
| 5A | 194.9 | IWA2646 | A/G | 0.35 | - | 1.4 | - | 1.3 | - | **2.6** | **2.3** |
| 5B | 0.0 | IWA868 | T/C | 0.19 | 757 | - | - | - | - | 1.9 | 1.6 |
| 5B | 68.3 | IWA7227 | T/C | 0.31 | - | - | **3.0** | 1.5 | - | **2.1** | 1.7 |
| 5B | 85.9 | IWA3633 | T/C | 0.54 | - | - | - | - | - | **2.8** | **2.8** |
| 5B | 119.9 | IWA4280 | A/G | 0.10 | 8069, 7815 | - | **24.0** | **8.0** | - | 1.7 | 1.6 |
| 5B | 205.8 | IWA22 | A/G | 0.26 | - | - | - | - | - | - | 1.6 |
| 6A | 75.5 | IWA4824 | A/G | 0.44 | - | 1.4 | - | **3.1** | 1.4 | 1.8 | 1.8 |
| 6A | 114.5 | IWA6596 | T/C | 0.51 | 1671, 5074, 6508 | - | - | - | - | 1.5 | **2.0** |
| 6A | 217.7 | IWA3066 | A/G | 0.72 | 3203, 3204 | 1.4 | - | - | - | **2.4** | **2.4** |
| 6B | 37.9 | IWA8134 | T/C | 0.10 | - | - | - | **2.3** | - | 1.7 | **2.5** |
| 6B | 38.5 | IWA2888 | T/C | 0.65 | 1905, 1657 | - | 1.6 | - | - | 1.6 | **2.4** |
| 6B | 50.8 | IWA7625 | A/G | 0.44 | 2419, 1655, 2417, 2420, 4823, 4825, 4827 | 1.9 | - | **3.1** | 1.6 | **2.3** | **2.3** |
| 6B | 84.5 | IWA6770 | A/G | 0.52 | 3289, 6660 | - | - | - | 1.4 | **2.5** | **2.6** |
| 6B | 112.3 | **IWA7257** | T/G | 0.23 | - | - | - | - | - | **5.0** | **3.2** |
| 6D | 68.6 | IWA7816 | A/G | 0.63 | - | **2.3** | - | - | - | **2.2** | **2.1** |
| 6D | 73.2 | **IWA167** | A/C | 0.10 | - | - | - | - | - | **5.5** | **4.5** |
| 7A | 6.2 | IWA7306 | A/G | 0.53 | - | 1.4 | - | - | - | 1.7 | **2.2** |
| 7A | 49.9 | IWA7121 | T/G | 0.70 | - | - | - | - | - | 1.6 | 1.6 |
| 7A | 105.5 | IWA7549 | A/C | 0.83 | - | - | - | - | - | **2.0** | **2.0** |
| 7B | 40.6 | IWA1108 | T/C | 0.27 | 6143, 8233 | - | - | - | - | 1.9 | 1.7 |
| 7B | 107.4 | IWA615 | T/C | 0.18 | - | - | 1.8 | - | 1.3 | **2.0** | 1.7 |

[a] Scaled position from hexaploid wheat consensus map (Cavanagh et al. 2013).

[b] SNP indexes from Illumina iSelect 9K wheat assay (Cavanagh et al. 2013). **Bold**: QTL with experiment –wise Bonferroni *P*< 0.1

[c] SNP variant associated to the resistant response is underlined.

[d] Frequency of the favorable SNP variant.

[e] SNP loci in LD with the representative SNP and significantly associated to the *Pst* response (IWA).

[f] IT= infection type , SEV= disease severity. IT-ALL and SEV-ALL, best linear unbiased estimates (BLUEs) over all environments.

[g] Significances are reported as -log (*P* value): '-' = not-significant, 1.3 = *P*=0.05, values > 2 are in **bold**, and value ≥ 4 (experiment-wise significant at α = 0.10) are in **bold underlined**.
