# Supplementary material for: A Genome-Wide Association Study of Resistance to Stripe Rust (Puccinia striiformis f. sp. tritici) in a Worldwide Collection of Hexaploid Spring Wheat (Triticum aestivum L.)
Source: G3 (Bethesda). 2015 Jan 20;5(3):449–65. doi: 10.1534/g3.114.014563 (PMC4349098; doi:10.1534/g3.114.014563)
Supplement: Supporting Information [file supp_g3.114.014563_TableS5.pdf]

Table S5 Loci associated with significant differences in partial resistance to *Pst* in at least three environments (one at *P*<0.01) in a collection of 593 spring hexaploid wheat with infection type ≥ 3.

Chr.	Pos.[a]	QTL-representative SNP			Associated SNP[e]	PSTv races IT (-log *P*)[g]				*Pst* response[f] (-log *P*)[g]	
	(cM)	Index[b]	Alleles[c]	Freq.[d]	IWA index	4	14	37	40	IT-ALL	SEV-ALL
1A	28.2	IWA414	T/G	0.62	4644, 4506	-	-	-	-	**2.4**	**2.8**
1A	111.2	IWA577	A/C	0.46	4538, 1081, 5832, 578	-	-	-	-	**3.1**	**2.9**
1A	148.1	IWA672 *	A/G	0.36	-	-	-	-	-	**-**	-
1B	93.5	IWA5769	A/G	0.20	5862, 8246, 3017	-	-	**4.0**	-	**2.0**	1.7
1B	123.4	**IWA3892 ***	A/G	0.70	846 [h]	-	-	-	1.3	**3.1**	**4.2**
1D	49.3	**IWA980**	A/C	0.44	642	-	-	-	-	1.7	1.7
1D	91.5	IWA2341	T/C	0.37	2340	**2.6**	-	1.8	1.5	**2.0**	**2.2**
2A	9.9	**IWA422 ***	T/C	0.31	423, 3468, 3469	-	1.6	-	-	**3.1**	1.9
2A	177.7	IWA3576	T/C	0.18	-	-	-	-	-	1.5	-
2B	126.3	IWA4606	T/C	0.39	1102, 1127, 1128, 1129, 1130, 1131, 1177, 1188 [i]	-	-	-	-	**2.3**	1.3
2B	149.4	IWA7312	A/G	0.81	-	-	-	-	-	**2.1**	1.9
2B	197.2	IWA5177	T/C	0.81	7615, 2379, 4900	-	-	-	-	**2.9**	1.9
3A	59.4	IWA6877	T/C	0.11	5039, 1308	-	-	-	**2.7**	1.5	-
3B	3.9	**IWA5202 ***	A/G	0.37	4796, 4801	-	-	-	-	**4.1**	**-**
3B	57.4	IWA6632 *	A/C	0.75	-	-	-	-	-	**2.0**	1.4
3B	88.1	IWA4085	T/C	0.68	7519, 8196	-	-	-	-	**2.6**	**2.2**
4A	167.3	IWA2170 *	A/G	0.37	7765, 1066, 6690	-	-	-	-	1.4	1.8
4A	181.7	**IWA1034 ***	T/C	0.17	1034, 6696	-	-	-	-	**4.1**	**2.7**
4B	26.4	IWA8109	A/G	0.72	7311	1.3	-	-	-	**2.8**	**2.4**
4B	116.4	IWA27	A/G	0.62	3781	-	-	-	-	**2.2**	**2.1**
5B	0.0	IWA868 *	T/C	0.19	757	-	-	-	-	**2.6**	1.7
5B	85.9	IWA3633 *	T/C	0.54	-	-	-	-	-	**2.3**	**3.0**
5B	115.3	IWA8069	A/G	0.28	2335, 2336, 8187	-	**4.1**	1.6	-	**2.7**	1.5
6A	138.6	IWA3491	T/C	0.84	4950	-	-	-	-	**2.3**	1.4
6B	0.6	IWA1493	A/C	0.14	1492	-	-	-	-	1.6	-
6B	60.1	IWA2090	A/G	0.20	3869, 3167, 3634, 2244	-	-	-	-	1.6	1.4
6B	112.3	**IWA7257 ***	T/G	0.23	-	-	-	-	-	**2.5**	1.4
6B	118.5	IWA404	T/C	0.69	405	-	-	-	-	**2.0**	**2.2**
6D2	73.2	**IWA167 ***	A/C	0.10	7816					**3.8**	**3.9**
7A	56.4	IWA3715	T/C	0.62	7161, 1156	-	-	1.5	-	1.4	1.5
7A	106.2	IWA2011	A/G	0.69	2009, 4288, 6004	-	-	-	-	1.9	**2.0**
7A	145.3	IWA4483	A/G	0.57	-	-	-	-	-	1.5	1.4
7B	48.0	IWA8525	A/G	0.26	-	-	-	-	-	-	-

7B	65.6	IWA3437	A/G	0.59	306, 3438, 3691, 3812, 4250, 507, 6712	-	-	-	-	1.8	1.9
7B	99.0	IWA4701	T/C	0.65	1345, 1346, 4160, 4701	-	1.4	-	-	**2.5**	**2.2**

[a] Scaled position from the hexaploid wheat consensus map (Cavanagh et al. 2013)

[b] SNP indexes from the Illumina iSelect 9K wheat assay (Cavanagh et al. 2013). **Bold**: QTL with Bonferroni P<0.1.

* Eleven SNPs that are also identified using all 875 accessions.

[c] SNP variant associated to the resistant response is underlined.

[d] Frequency of the favorable SNP variant.

[e] SNP loci in LD with the representative SNP and significantly associated to the *Pst* response.

[f] IT= infection type, SEV= disease severity.IT-ALL and SEV-ALL, best linear unbiased estimates (BLUEs) over all environments.

[g] Significances are reported as -log (*P* value) as follows: '-' = not-significant, 1.3 = *P*=0.05, values > 2 are in **bold**, and value ≥ 4 (experiment-wise significant at α = 0.10) are in **bold underlined**.

[h] Additional associated SNPs: IWA1092, 919, 920, 7992, 8322, 3893, 846, 1791, 724, 7892, 6831

[i] Additional associated SNPs: IWA1102, 1127, 1128, 1129, 1130, 1131, 1177, 1188, 1229, 1237, 4604, 4606, 771, 776, 777, 829, 869, 874
