# Supplementary material for: A Genome-Wide Association Study of Resistance to Stripe Rust (Puccinia striiformis f. sp. tritici) in a Worldwide Collection of Hexaploid Spring Wheat (Triticum aestivum L.)
Source: G3 (Bethesda). 2015 Jan 20;5(3):449–65. doi: 10.1534/g3.114.014563 (PMC4349098; doi:10.1534/g3.114.014563)
Supplement: Supporting Information [file supp_g3.114.014563_TableS6.pdf]

Table S6 Frequencies across the seven subpopulations of favorable alleles for the QTL-tagging SNPs detected in the complete GWAS (875 accessions).

Chr.	Pos.[a]	QTL-representative SNP			Pop. genetic structure (sub-groups by Ward clustering)						
					**1A**	**1B**	**2**	**3**	**4A**	**4B**	**4C**
	(cM)	Index[b]	Alleles[c]	Freq.[d]	(134)	(84)	(169)	(285)	(90)	(69)	(44)
1A	39.0	IWA6441	T/C	0.56	0.62	0.73	0.56	0.59	0.58	0.49	0.37
1A	58.0	IWA5194	A/G	0.32	0.34	0.05	0.17	0.63	0.09	0.10	0.56
1A	59.6	IWA4061	A/G	0.63	0.83	0.96	0.84	0.34	0.51	0.84	0.71
1A	84.2	IWA3475	T/C	0.58	0.71	0.48	0.6	0.32	0.87	0.99	0.86
1A	88.1	IWA5174	A/C	0.39	0.59	0.64	0.33	0.51	0.11	0.03	1.00
1A	120.3	IWA1225	T/G	0.69	0.74	0.64	0.75	0.51	0.93	0.97	1.00
1A	148.1	IWA672	A/G	0.36	0.25	0.38	0.53	0.56	0.12	0.10	0.00
1A	158.6	IWA7893	T/C	0.83	0.68	0.77	0.95	0.94	0.83	0.59	0.93
1A	173.7	IWA2035	A/G	0.63	0.69	0.63	0.65	0.86	0.38	0.36	0.05
1B	35.5	IWA962	A/G	0.14	0.19	0.67	0.07	0.06	0.19	0.00	0.86
1B	51.9	IWA6758	A/G	0.31	0.36	0.55	0.35	0.26	0.15	0.46	0.95
1B	57.6	IWA3307	T/C	0.49	0.64	0.12	0.36	0.62	0.49	0.60	0.19
1B	94.7	IWA3017	A/G	0.11	0.23	0.06	0.04	0.14	0.12	0.01	0.23
1B	109.3	IWA1825	T/C	0.35	0.37	0.23	0.58	0.47	0.07	0.29	0.02
1B	123.4	**IWA3892**	A/G	0.70	0.77	0.87	0.70	0.76	0.53	0.77	0.70
1B	141.2	IWA2077	A/G	0.18	0.15	0.05	0.05	0.15	0.61	0.41	0.05
1D	21.4	IWA2547	T/C	0.11	0.19	0.05	0.10	0.16	0.02	0.10	0.12
2A	9.9	**IWA422**	T/C	0.31	0.35	0.10	0.19	0.25	0.56	0.77	0.93
2A	62.2	IWA3520	A/C	0.52	0.34	0.25	0.54	0.72	0.36	0.62	0.21
2A	78.3	**IWA424**	T/C	0.70	0.48	0.83	0.80	0.88	0.26	0.97	0.35
2A	96.2	IWA5272	A/G	0.52	0.51	0.68	0.33	0.78	0.07	0.86	0.00
2A	110.6	IWA7947	A/C	0.71	0.73	0.88	0.60	0.82	0.64	0.75	0.49
2A	160.2	IWA200	A/G	0.84	0.87	0.92	0.80	0.83	0.93	1.00	0.93
2B	112.3	IWA905	A/G	0.82	0.65	0.95	0.86	0.81	0.98	0.84	1.00
2B	147.3	IWA586	T/C	0.62	0.52	0.79	0.78	0.70	0.34	0.68	0.91
2B	163.4	IWA226	T/C	0.54	0.54	0.44	0.34	0.64	0.88	0.52	0.98
2B	266.4	IWA3206	T/C	0.12	0.26	0.31	0.18	0.06	0.01	0.04	0.00
3A	13.2	IWA5969	T/C	0.14	0.09	0.42	0.24	0.22	0.02	0.01	0.07
3A	27.5	IWA2049	T/C	0.14	0.35	0.20	0.14	0.07	0.15	0.01	0.88
3A	35.0	IWA1996	A/G	0.87	0.88	0.93	0.96	0.78	0.98	0.93	1.00
3A	59.4	IWA5039	T/C	0.64	0.52	0.81	0.85	0.79	0.24	0.83	0.19
3A	70.4	IWA8215	T/G	0.27	0.36	0.05	0.11	0.41	0.13	0.48	0.00
3A	75.2	IWA7011	A/G	0.24	0.15	0.04	0.18	0.31	0.13	0.79	0.02
3A	102.9	IWA2332	A/G	0.35	0.42	0.26	0.54	0.24	0.22	0.54	0.93
3B	3.9	**IWA5202**	A/G	0.37	0.41	0.52	0.47	0.40	0.09	0.32	0.12
3B	57.4	IWA6632	A/C	0.75	0.89	0.85	0.83	0.69	0.54	0.83	0.44
3B	73.8	IWA377	T/C	0.76	0.96	0.75	0.96	0.87	0.23	0.61	0.98
3B	77.5	IWA8480	A/G	0.14	0.13	0.12	0.09	0.18	0.15	0.10	0.98
3B	84.5	IWA5890	T/C	0.53	0.61	0.81	0.40	0.62	0.34	0.41	1.00
3B	95.5	IWA6221	T/C	0.75	0.91	0.67	0.85	0.81	0.49	0.64	1.00

3B	101.4	IWA321	T/C	0.11	0.29	0.27	0.16	0.02	0.00	0.00	0.00
4A	35.2	IWA6100	A/G	0.33	0.09	0.16	0.34	0.39	0.49	0.75	0.09
4A	44.0	IWA1992	A/G	0.58	0.56	0.25	0.42	0.68	0.60	0.78	0.93
4A	54.9	IWA7216	T/C	0.53	0.59	0.45	0.23	0.48	0.81	0.91	1.00
4A	68.1	IWA1570	T/G	0.17	0.55	0.06	0.10	0.10	0.16	0.01	1.00
4A	167.3	IWA2170	A/G	0.37	0.53	0.49	0.36	0.31	0.50	0.23	0.86
4A	181.7	**IWA1034**	T/C	0.17	0.08	0.04	0.03	0.17	0.39	0.28	0.00
4B	85.2	IWA6461	T/C	0.54	0.66	0.81	0.83	0.59	0.15	0.09	0.09
4D	26.9	**IWA5375**	T/G	0.10	0.38	0.12	0.05	0.02	0.10	0.01	0.88
5A	4.9	IWA2144	T/C	0.19	0.37	0.45	0.21	0.18	0.02	0.01	0.6
5A	119.3	IWA1486	T/C	0.24	0.28	0.19	0.28	0.27	0.08	0.67	0.28
5A	189.2	**IWA6988**	T/C	0.16	0.17	0.10	0.12	0.15	0.38	0.23	0.88
5A	194.9	IWA2646	A/G	0.35	0.35	0.31	0.62	0.39	0.27	0.33	0.14
5B	0.0	IWA868	T/C	0.19	0.05	0.00	0.11	0.23	0.43	0.35	0.02
5B	68.3	IWA7227	T/C	0.31	0.16	0.58	0.18	0.4	0.45	0.47	0.00
5B	85.9	IWA3633	T/C	0.54	0.69	0.83	0.57	0.61	0.07	0.03	0.00
5B	119.9	IWA4280	A/G	0.10	0.16	0.49	0.09	0.08	0.01	0.00	0.00
5B	205.8	IWA22	A/G	0.26	0.19	0.22	0.24	0.28	0.29	0.52	0.00
6A	75.5	IWA4824	A/G	0.44	0.21	0.1	0.71	0.38	0.78	0.42	0.98
6A	114.5	IWA6596	T/C	0.51	0.51	0.4	0.60	0.73	0.22	0.38	0.02
6A	217.7	IWA3066	A/G	0.72	0.84	0.63	0.91	0.70	0.73	0.48	0.91
6B	37.9	IWA8134	T/C	0.10	0.05	0.01	0.25	0.17	0.1	0.04	0.02
6B	38.5	IWA2888	T/C	0.65	0.87	0.45	0.78	0.61	0.82	0.58	0.98
6B	50.8	IWA7625	A/G	0.44	0.20	0.10	0.71	0.38	0.78	0.42	0.98
6B	84.5	IWA6770	A/G	0.52	0.18	0.54	0.38	0.70	0.76	0.28	0.79
6B	112.3	**IWA7257**	T/G	0.23	0.61	0.32	0.51	0.11	0.06	0.01	0.00
6D2	68.6	IWA7816	A/G	0.63	0.67	0.52	0.70	0.49	0.91	0.86	1.00
6D2	73.2	**IWA167**	A/C	0.10	0.36	0.10	0.03	0.06	0.05	0.01	0.95
7A	6.2	IWA7306	A/G	0.53	0.45	0.74	0.50	0.56	0.56	0.87	0.29
7A	49.9	IWA7121	T/G	0.70	0.68	0.83	0.51	0.76	0.64	0.99	0.88
7A	105.5	IWA7549	A/C	0.83	0.84	1.00	0.83	0.77	0.94	1.00	1.00
7B	40.6	IWA1108	T/C	0.27	0.22	0.42	0.25	0.33	0.17	0.35	0.16
7B	107.4	IWA615	T/C	0.18	0.18	0.18	0.10	0.27	0.20	0.03	1.00
N. of favorable alleles with freq. >0.90					2	5	4	1	6	8	26
N. of favorable alleles with freq. <0.10					5	10	9	8	15	17	21
Percent >0.9 + <0.10 by subpopulation					9.6	20.5	17.8	12.3	28.8	34.2	**64.4**
Average Genetic diversity (based on all SNPs)					0.39	0.34	0.34	0.38	0.27	0.26	**0.13**

[a] Scaled position from hexaploid wheat consensus map (Cavanagh et al. 2013).

[b] SNP indexes from Illumina iSelect 9K wheat assay (Cavanagh et al. 2013).

[c] SNP variant associated to the resistant response is underlined.

[d] Frequency of the favorable SNP variant.

Bold name: SNPs significant for Bonferroni P<0.10 (experiment-wise error rate), yellow: allele frequency < 0.1, green: allele frequency > 0.9.
