# Supplementary material for: A Genome-Wide Association Study of Resistance to Stripe Rust (Puccinia striiformis f. sp. tritici) in a Worldwide Collection of Hexaploid Spring Wheat (Triticum aestivum L.)
Source: G3 (Bethesda). 2015 Jan 20;5(3):449–65. doi: 10.1534/g3.114.014563 (PMC4349098; doi:10.1534/g3.114.014563)
Supplement: Supporting Information [file supp_g3.114.014563_TableS7.pdf]

Table S7 Frequencies across the seven subpopulations of the favorable alleles for SNPs detected in the GWAS for partial resistance (593 accessions). Only the 24 QTL that were not redundant with Table S6 are listed below.

Chr.	Pos.[a]	QTL-representative SNP			Pop. genetic structure (sub-groups by Ward clustering)						
	(cM)	Index[b]	Alleles[c]	Freq.[d]	**1A**	**1B**	**2**	**3**	**4A**	**4B**	**4C**
					134	84	169	285	90	69	44
1A	28.2	IWA414	T/G	0.62	0.72	0.94	0.73	0.50	0.48	0.63	0.95
1A	111.2	IWA577	A/C	0.46	0.47	0.29	0.50	0.28	0.67	0.78	1.00
1B	93.5	IWA5769	A/G	0.2	0.26	0.08	0.07	0.24	0.44	0.01	0.36
1D	49.3	**IWA980**	A/C	0.44	0.19	0.17	0.70	0.60	0.34	0.43	0.20
1D	91.5	IWA2341	T/C	0.37	0.69	0.22	0.63	0.28	0.11	0.29	0.00
2A	177.7	IWA3576	T/C	0.18	0.12	0.02	0.19	0.02	0.41	0.62	0.11
2B	126.3	IWA4606	T/C	0.39	0.49	0.66	0.43	0.43	0.07	0.49	0.02
2B	149.4	IWA7312	A/G	0.81	0.55	0.86	0.82	0.86	0.94	1.00	0.91
2B	197.2	IWA5177	T/C	0.81	0.92	0.98	0.82	0.90	0.60	0.71	0.09
3A	59.4	IWA6877	T/C	0.11	0.15	0.60	0.07	0.14	0.00	0.00	0.00
3B	88.1	IWA4085	T/C	0.68	0.54	0.80	0.72	0.69	0.79	0.43	1.00
4B	26.4	IWA8109	A/G	0.72	0.75	0.88	0.73	0.75	0.53	0.62	0.98
4B	116.4	IWA27	A/G	0.62	0.79	0.82	0.81	0.56	0.15	0.59	1.00
5B	115.3	IWA8069	A/G	0.28	0.25	0.51	0.33	0.30	0.06	0.46	0.02
6A	138.6	IWA3491	T/C	0.84	0.81	0.60	0.95	0.87	0.67	0.97	0.77
6B	0.6	IWA1493	A/C	0.14	0.35	0.15	0.18	0.11	0.00	0.00	0.00
6B	60.1	IWA2090	A/G	0.2	0.60	0.40	0.07	0.09	0.00	0.00	0.00
6B	118.5	IWA404	T/C	0.69	0.92	0.81	0.79	0.54	0.70	0.71	1.00
7A	56.4	IWA3715	T/C	0.62	0.59	0.63	0.44	0.78	0.69	0.58	0.89
7A	106.2	IWA2011	A/G	0.69	0.78	0.79	0.55	0.65	0.92	0.54	0.98
7A	145.3	IWA4483	A/G	0.57	0.82	0.69	0.69	0.46	0.56	0.19	0.86
7B	48.0	IWA8525	A/G	0.26	0.23	0.14	0.14	0.29	0.50	0.17	0.86
7B	65.6	IWA3437	A/G	0.59	0.80	0.75	0.40	0.70	0.44	0.19	0.98
7B	99.0	IWA4701	T/C	0.65	0.70	0.79	0.69	0.53	0.68	0.68	0.23
N. of favorable alleles with freq. >0.90					2	2	1	0	2	2	8
N. of favorable alleles with freq. <0.10					0	2	3	1	5	4	7
Percent >0.9 + <0.10 by subpopulation					8.3	16.7	16.7	4.2	29.2	25.0	**62.5**

[a] Scaled position from hexaploid wheat consensus map (Cavanagh et al. 2013).

[b] SNP indexes from Illumina iSelect 9K wheat assay (Cavanagh et al. 2013).

[c] SNP variant associated to the resistant response is underlined.

[d] Frequency of the favorable SNP variant.

Bold name: SNPs significant for Bonferroni P<0.10 (experiment-wise error rate), yellow: allele frequency < 0.1, green: allele frequency > 0.9.
