# Supplementary material for: A Genome-Wide Association Study of Resistance to Stripe Rust (Puccinia striiformis f. sp. tritici) in a Worldwide Collection of Hexaploid Spring Wheat (Triticum aestivum L.)
Source: G3 (Bethesda). 2015 Jan 20;5(3):449–65. doi: 10.1534/g3.114.014563 (PMC4349098; doi:10.1534/g3.114.014563)
Supplement: Supporting Information [file supp_g3.114.014563_TableS8.pdf]

Table S8 ANOVA for *Pst* infection type (IT) and severity (SEV) based on 10 significant QTL and population structure (Q7) as covariable. P values are presented for the structure alone, the structure and the 10 QTL, and the structure plus the 10 QTL plus the significant interactions.

	Structure alone		Structure & 10 QTL		Structure & 10 QTL & 6 int.	
Source	**IT**	**SEV**	**IT**	**SEV**	**IT**	**SEV**
Q1	0.0086	0.0011	0.3547	0.3673	0.7763	0.7429
Q2	0.0544	0.0386	0.0019	0.0034	0.0004	0.0008
Q3	<.0001	<.0001	0.7441	0.6287	0.234	0.6761
Q4	<.0001	<.0001	0.6426	0.8195	0.2053	0.2891
Q5	0.0898	0.0395	0.3596	0.4114	0.1244	0.1339
Q6	<.0001	<.0001	0.0001	<.0001	0.0014	<.0001
IWA980			0.0001	<.0001	0.0096	0.0092
IWA3892			0.0001	0.0001	0.0001	<.0001
IWA1034			0.0020	0.0083	0.0003	0.0003
IWA422			<.0001	<.0001	0.1813	0.2471
IWA424			0.1756	0.0429	0.6596	0.4986
IWA5202			<.0001	0.0526	0.3835	0.6191
IWA5375			0.0005	<.0001	0.0024	0.0005
IWA6988			0.0008	0.0072	0.0002	0.0017
IWA7257			<.0001	0.0004	0.0102	0.1154
IWA167			<.000	<.0001	<.0001	<.0001
IWA1034*IWA424					0.0419	0.0023
IWA980*IWA5202					0.0447	0.0877
IWA422*IWA6988					<.0001	<.0001
IWA422*IWA7257					0.0011	0.0034
IWA5202*IWA167					0.0259	0.0191
IWA6988*IWA167					0.0047	0.0087
R^2	0.141	0.167	0.290	0.285	0.330	0.324
