# Supplementary material for: A Genome-Wide Association Study of Resistance to Stripe Rust (Puccinia striiformis f. sp. tritici) in a Worldwide Collection of Hexaploid Spring Wheat (Triticum aestivum L.)
Source: G3 (Bethesda). 2015 Jan 20;5(3):449–65. doi: 10.1534/g3.114.014563 (PMC4349098; doi:10.1534/g3.114.014563)
Supplement: Supporting Information [file supp_g3.114.014563_TableS9.pdf]

Table S9 Pairwise fixation indexes (*Fst*) among populations based on 4,585 SNPs. All comparisons are significant at $P < 0.001$ with 1000 permutations.

Subgroup	1A	1B	2	3	4A	4B	4C
1A	0.000						
1B	0.105	0.000					
2	0.109	0.127	0.000				
3	0.106	0.117	0.087	0.000			
4A	0.213	0.276	0.255	0.225	0.000		
4B	0.212	0.263	0.202	0.184	0.200	0.000	
4C	0.304	0.427	0.402	0.360	0.296	0.447	0.000
