# Supplementary material for: A Genome-Wide Association Study of Resistance to Stripe Rust (Puccinia striiformis f. sp. tritici) in a Worldwide Collection of Hexaploid Spring Wheat (Triticum aestivum L.)
Source: G3 (Bethesda). 2015 Jan 20;5(3):449–65. doi: 10.1534/g3.114.014563 (PMC4349098; doi:10.1534/g3.114.014563)
Supplement: Supporting Information [file supp_g3.114.014563_TableS10.pdf]

Table S10 Number of 9K SNP mapped and 90K SNP projected into the confidence intervals of the 10 significant QTL described in Table 3. A tentative annotation for the wheat contigs including these SNPs was inferred from annotations in the Phytozome database based on the best hits in rice and *Brachypodium* protein databases. Detailed information for each SNP is provided in supplemental **File S2**. Plant resistance proteins (*R* genes) include CC-NB-LRR (coiled-coil domain, nucleotide binding site, leucine-rich repeat), RLP (receptor like proteins coupled with extracellular LRR) and RLK (kinase domain coupled with LRR).

Marker	Chr.	Position	No. SNP in QTL CI			Functional annotation	
		cM	9k-anchor SNP	Additional 90k-projected SNP	Total	No. of annotated orthologs	No. of *R* genes
IWA3892	1B	123.4	8	41	49	26	5
IWA980	1D	49.3	14	110	124	68	5
IWA422	2A	9.9	24	194	218	140	35
IWA424	2A	78.3	27	201	218	146	3
IWA5202	3B	3.9	18	88	106	74	16
IWA1034	4A[a]	181.7	12	11	23	19	10
IWA5375	4D	26.9	7	49	56	47	1
IWA6988	5A[b]	189.2	3	21	24	15	0
IWA7257	6B	112.3	4	24	28	19	0
IWA167	6D	73.2	8	64	76	54	2
**Total**			119	803	922	608	77

[a] *IWA1034* is in the region of 4AL translocated from 7BS (homology 7AS/4AL/7DS).
[b] *IWA6988* is in the region of 5AL translocated from 4AL (homology 5AL/4BL/4DL).
