# Supplementary material for: A Genome-Wide Association Study of Resistance to Stripe Rust (Puccinia striiformis f. sp. tritici) in a Worldwide Collection of Hexaploid Spring Wheat (Triticum aestivum L.)
Source: G3 (Bethesda). 2015 Jan 20;5(3):449–65. doi: 10.1534/g3.114.014563 (PMC4349098; doi:10.1534/g3.114.014563)
Supplement: Supporting Information [file supp_g3.114.014563_FigureS1.pdf]

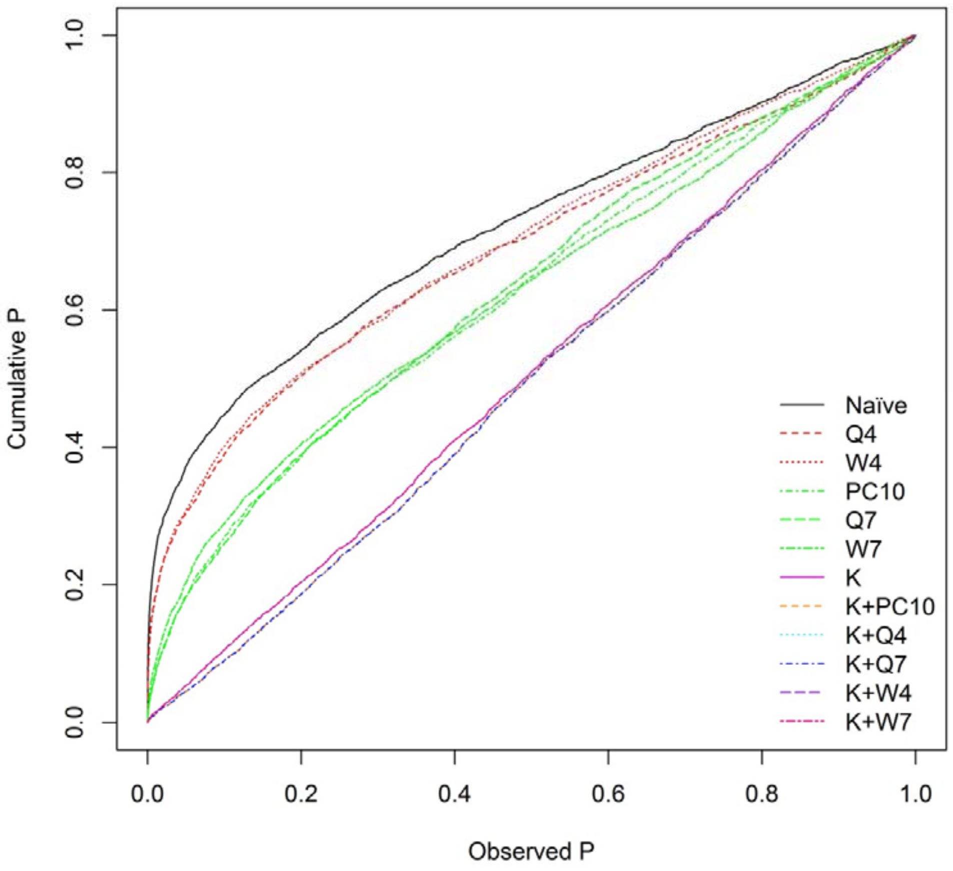

Figure S1 Plot of observed *vs.* expected cumulative *P* values using different GWAS models. The compared models are based on the IT data from UC Davis, 2011. **Naïve**: no population structure correction; **Q4 and Q7**: general linear model (GLM) with STRUCTURE membership coefficients from the four main groups or the 7 subgroups as covariates; **Ward 4 and Ward 7**: GLM with Ward cluster coefficients from the four main groups or the 7 subgroups as covariates; **PC10**: GLM with first 10 principal components as covariates; **K**: mixed linear model (MLM) with the 875 × 875 kinship matrix of identity-by-state (IBS). **K** was then combined with the previous 5 models (**K+Q4, K+Q7, K+W4, K+W7, K+PC10**). Genetic similarities were calculated using all 4,585 SNPs.
