# Supplementary material for: A Genome-Wide Association Study of Resistance to Stripe Rust (Puccinia striiformis f. sp. tritici) in a Worldwide Collection of Hexaploid Spring Wheat (Triticum aestivum L.)
Source: G3 (Bethesda). 2015 Jan 20;5(3):449–65. doi: 10.1534/g3.114.014563 (PMC4349098; doi:10.1534/g3.114.014563)
Supplement: Supporting Information [file supp_g3.114.014563_FigureS2.pdf]

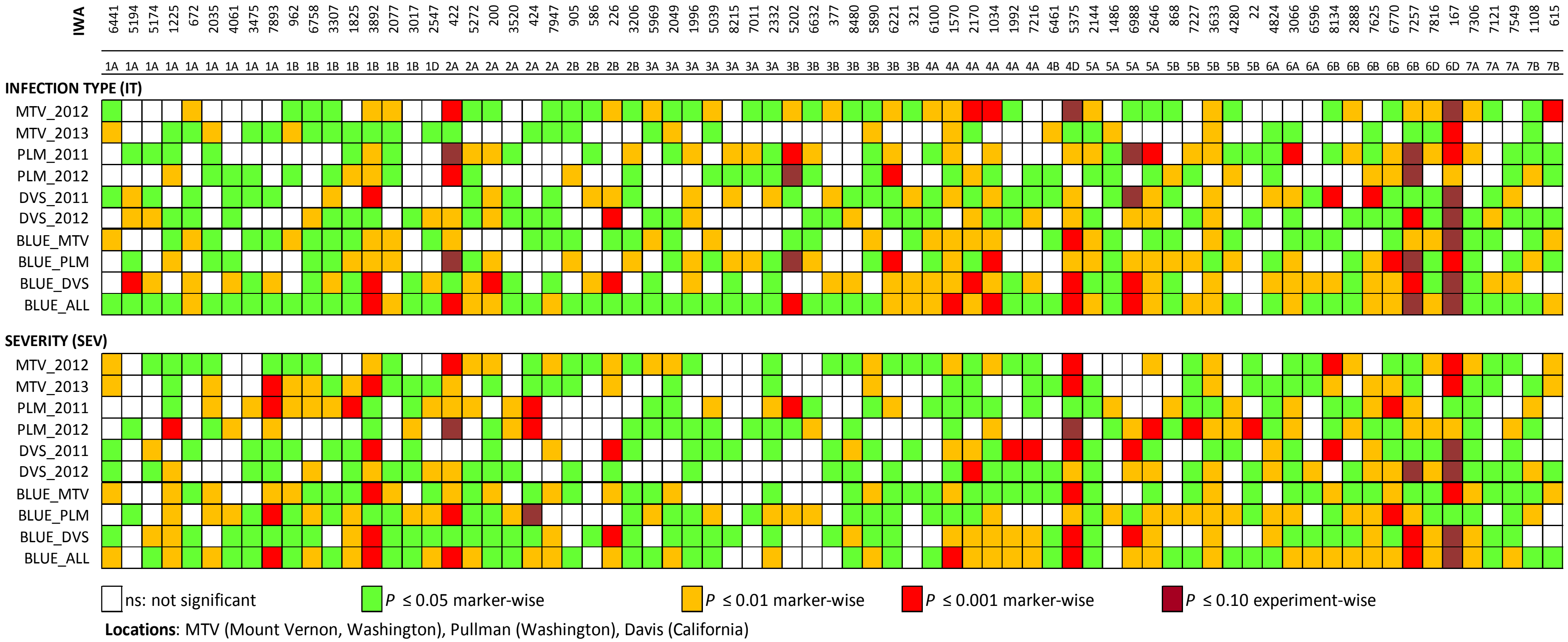

Figure S2 Selected QTL for *Pst* infection type (IT) and disease severity (SEV) in a collection of 875 spring hexaploid wheat. Only QTL significant in at least 3 environments (with at least one with *P*<0.01) are reported. Best Linear Unbiased Estimates (BLUEs) are calculated for each location separately and for the 6 combined environments (BLUE_ALL).
