# Supplementary material for: A Genome-Wide Association Study of Resistance to Stripe Rust (Puccinia striiformis f. sp. tritici) in a Worldwide Collection of Hexaploid Spring Wheat (Triticum aestivum L.)
Source: G3 (Bethesda). 2015 Jan 20;5(3):449–65. doi: 10.1534/g3.114.014563 (PMC4349098; doi:10.1534/g3.114.014563)
Supplement: Supporting Information [file supp_g3.114.014563_FigureS3.pdf]

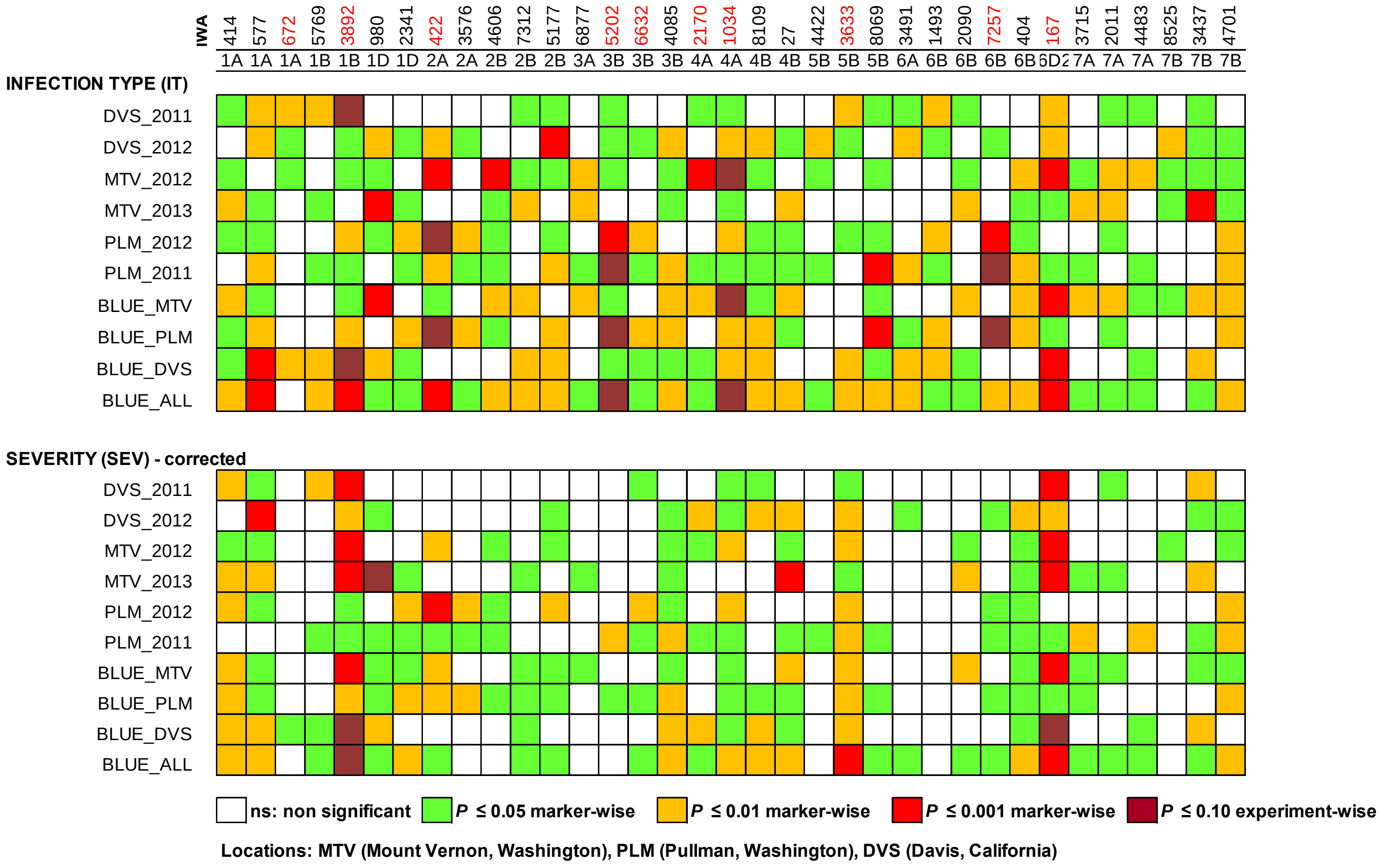

Figure S3 Selected QTL for partial resistance to *Pst* in a collection of 593 accessions of spring wheat with IT≥3. Only QTL significant in at least 3 environments (with at least one of them with $P<0.01$) are reported. SNP names in red are also significant in the complete analysis in **Figure S2**. Best linear unbiased estimates (BLUEs) are calculated for each location and for the 6 combined environments (BLUE_ALL).
