# Supplementary material for: A Genome-Wide Association Study of Resistance to Stripe Rust (Puccinia striiformis f. sp. tritici) in a Worldwide Collection of Hexaploid Spring Wheat (Triticum aestivum L.)
Source: G3 (Bethesda). 2015 Jan 20;5(3):449–65. doi: 10.1534/g3.114.014563 (PMC4349098; doi:10.1534/g3.114.014563)
Supplement: Supporting Information [file supp_g3.114.014563_FigureS4.pdf]

A

Infection Type (Y axis) *vs.* number of favorable SNP variants (X axis)

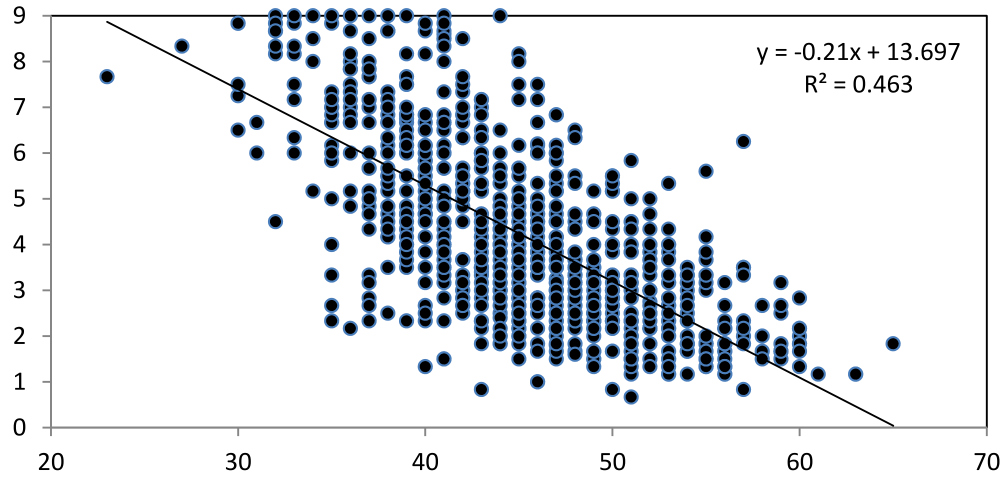

B

Disease severity (Y axis) *vs.* number of favorable SNP variants (X axis)

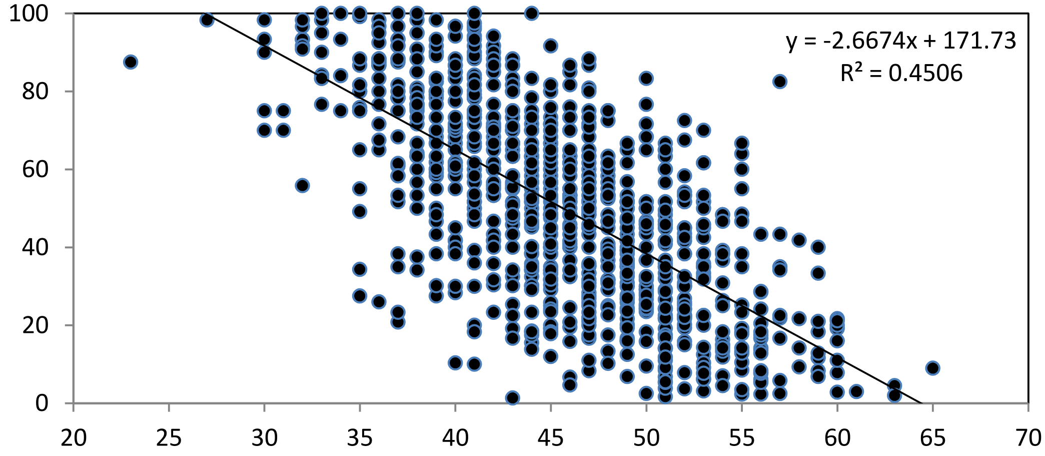

Figure S4 Regression between **(A)** Infection type (IT) and **(B)** disease severity (SEV) and the number of favorable alleles in each of the 875 lines. Both regressions were highly significant $P<0.0001$. Original data is available in **File S1**.
