# Supplementary material for: A Genome-Wide Association Study of Resistance to Stripe Rust (Puccinia striiformis f. sp. tritici) in a Worldwide Collection of Hexaploid Spring Wheat (Triticum aestivum L.)
Source: G3 (Bethesda). 2015 Jan 20;5(3):449–65. doi: 10.1534/g3.114.014563 (PMC4349098; doi:10.1534/g3.114.014563)
Supplement: Supporting Information [file supp_g3.114.014563_FigureS5.pdf]

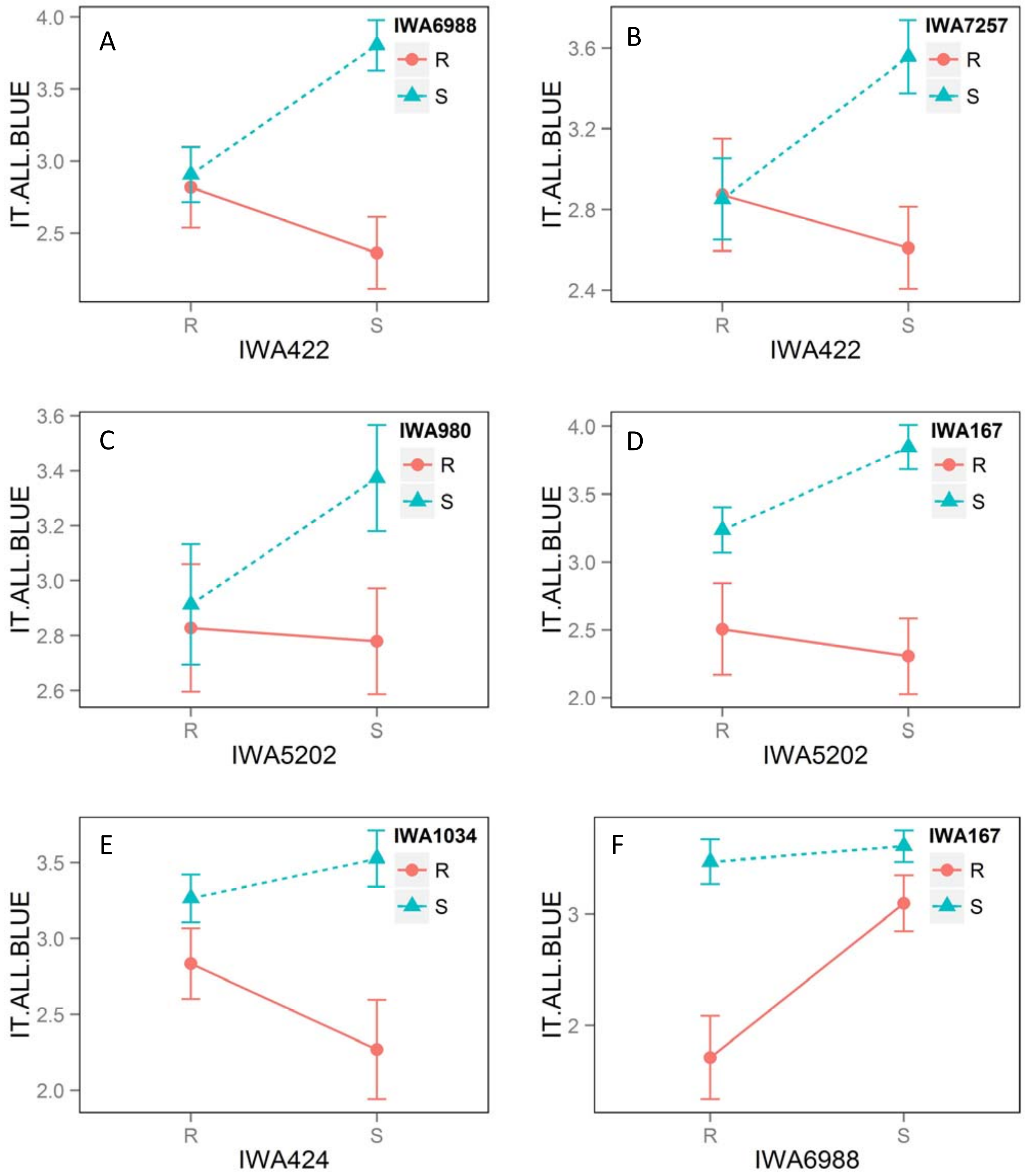

Figure S5 Significant interactions among 10 selected QTLs (**Table S8**). IT.ALL.BLUE values are the least square means from the full model ANOVA for BLUE values across all locations (**Table S8**) ± the standard error of the least square means.
