# Supplementary material for: A Genome-Wide Association Study of Resistance to Stripe Rust (Puccinia striiformis f. sp. tritici) in a Worldwide Collection of Hexaploid Spring Wheat (Triticum aestivum L.)
Source: G3 (Bethesda). 2015 Jan 20;5(3):449–65. doi: 10.1534/g3.114.014563 (PMC4349098; doi:10.1534/g3.114.014563)
Supplement: Supporting Information [file supp_g3.114.014563_FileS5.pdf]

A Genome-Wide Association Study of Resistance to Stripe Rust (*Puccinia striiformis* f. sp. *tritici*) in a Worldwide Collection of Hexaploid Spring Wheat (*Triticum aestivum* L.)

Marco Maccaferri[*,§1], Junli Zhang[*1], Peter Bulli[†1], Zewdie Abate[*], Shiaoman Chao[**], Dario Cantu[***], Eligio Bossolini[*], Xianming Chen[††], Michael Pumphrey[†], Jorge Dubcovsky[*,†††, 2]

[*] Department of Plant Sciences, University of California, Davis, CA 95616, USA

[§] Department of Agricultural Sciences (DipSA), University of Bologna, Bologna 40127, Italy

[†] Department of Crop and Soil Sciences, Washington State University, Pullman, WA 99164-6420, USA

[††] USDA-ARS, Wheat Genetics, Quality Physiology, and Disease Research Unit, and Department of Plant Pathology, Washington State University, Pullman, WA 99164, USA

[**] USDA-ARS, 1605 Albrecht Blvd, Fargo, ND 58105, USA

[***] Department of Viticulture and Enology, University of California, Davis, CA 95616, USA

[†††] Howard Hughes Medical Institute, Chevy Chase, MD 20815, USA

[1] The first three authors contributed equally to this study.

[2] Corresponding author: J. Dubcovsky. Email: jdubcovsky@ucdavis.edu Phone: (530) 752-5159

Supplemental File S5. Supporting data for Figure 6.

SUPPLEMENTAL INFORMATION FOR FIGURE 6

Table S11 in this supplemental file summarizes the information used to generate **Figure 6.** The 10 experiment-wise significant QTL described in **Table 3** and previously mapped *Pst* resistance genes and QTL were both projected onto the integrated map reported in supplemental **File S4**. These 10 regions are discussed in detail after the Table.

Table S11 includes chromosome name, QTL start and end position (as % of the total length of the chromosomes in the integrated map described in **Supplemental File S4**), the name of the gene or QTL, the name of the parent that contributed the resistant allele, the reference used to obtain this information and the ID number used in **Figure 6**. For genes included in the Catalogue of Gene Symbols for Wheat (McIntosh *et al.* 2013) or the 2013-2014 Supplement (http://wheat.pw.usda.gov/GG2/Triticum/wgc/2013/2013-2014_Supplement.pdf), "Wheat Catalogue" is indicated as reference, except if more recent or precise references are available (a single paper is selected).

For the named *Yr* genes previously mapped as Mendelian loci, the position in **Figure 6** is based on the projection of its reported position onto the integrated map, and the confidence interval is based on published flanking markers (**Table S11**). The 10 significant QTL identified in this study are presented with confidence interval of ± 1.6 cM as discussed in the main text. Gene positions and confidence intervals presented here should be considered as tentative because of the approximate nature of the integrated map (**Supplemental File S4**), which is caused in part by the limited numbers of common markers between SNP-based and SSR-based maps. The objective of this figure is to identify previously mapped *Pst* resistance genes and QTL present in the QTL regions identified in this study. Further allelism studies will be necessary to determine if closely mapped loci represent different resistance genes or alleles of the same gene.

Table S11. *Pst* resistance genes and QTL included in Figure 6 (organized by chromosome)

Chr	Start (%)	End (%)	*QTL*	Reference	Ref_No
1A	0.0	5.7	*QYrid.ui-1A_Rio Blanco*	Chen *et al.* 2012	67
1A	8.1	10.2	*QYr.tam-1A_Avocet-YrA*	Basnet *et al.* 2014b	5
1A	16.5	20.1	*QYr.sgi-1A.1_Kariega*	Prins *et al.* 2011	40
1A	21.7	31.8	*QYr.tam-1AS_TAM111*	Basnet *et al.* 2014a	6
1A	24.5	46.8	*QYr.sun-1A_Janz*	Bariana *et al.* 2010	4
1A	75.7	100.0	*QYr.tam-1AL_TAM112*	Basnet *et al.* 2014a	6
1A	77.8	80.3	*QYr.caas-1AL_Naxos*	Ren *et al.* 2012a	45
1A	81.9	83.0	*QYr.cim-1AL_Pastor*	Rosewarne *et al.* 2012	48
1B	0.0	1.7	*Yr10*	Ma *et al.* 2001	31
1B	1.7	9.3	*Yr9*	Lukaszewski 2000	68
1B	5.1	28.0	*QYr.cau-1BS_AQ24788-53*	Quan *et al.* 2013	42
1B	24.8	34.6	*YrAlp*	Wheat Catalogue [a]	66
1B	28.2	34.6	*QYr.caas-1BL.1RS_SHA3/CBRD*	Ren *et al.* 2012a	45
1B	30.1	32.1	*Yr15*	Cheng *et al.* 2014	10
1B	30.9	32.7	*YrH52*	Cheng *et al.* 2014	10
1B	32.7	34.5	*Yr64*	Cheng *et al.* 2014	10
1B	37.4	39.0	*Yr65*	Cheng *et al.* 2014	10
1B	39.0	41.3	*Yr24/Yr26*	Cheng *et al.* 2014	10

1B	41.2	54.5	*QYr.cim-1BS_Pastor*	Rosewarne *et al.* 2012	48
1B	61.3	67.2	*QYr-1B_Sachem*	Singh *et al.* 2013	51
1B	70.8	75.2	*YrExp1*	Wheat Catalogue	66
1B	75.3	100.0	*QYrex.wgp-1BL_Express*	Lin and Chen 2009	25
1B	80.0	96.7	*QYr.sun-1B_Kukri*	Bariana *et al.* 2010	4
1B	80.0	90.2	*QYr.sun-1B_CPI133872*	Zwart *et al.* 2010	65
1B	84.2	96.3	*QYr.sun-1B_Wollaroi*	Bansal *et al.* 2014	3
1B	90.0	100.0	*Yr29/Lr46*	Lan *et al.* 2014	21
1B	87.9	89.7	***QYr.ucw-1B (IWA3892)***	This study	
1B	90.1	94.8	*QYr.jic-1B_Guardian*	Melichar *et al.* 2008	35
1B	90.2	98.7	*QYr.tam-1B_Quaiu*	Basnet *et al.* 2014b	5
1B	90.2	98.7	*QYr.cim-1BL_Francolin*	Lan *et al.* 2014	21
1B	90.3	96.3	*QYr-1B_Saar*	Lillemo *et al.* 2008	24
1B	93.6	100.0	*QYr.cim-1BL_Pastor (Lr46/Yr29)*	Rosewarne *et al.* 2012	48
1D	0.8	8.5	*QYr.caas-1DS_Naxos*	Ren *et al.* 2012a	45
1D	1.4	10.1	*QYrst.orr-1DS_Stephens*	Vazquez *et al.* 2012	55
1D	5.1	8.5	*QYr.sun-1D_CPI133872*	Zwart *et al.* 2010	65
1D	24.0	25.6	***QYr.ucw-1D (IWA980)***	This study	
2A	0.0	6.1	*QYr.tam-2AS_TAM111*	Basnet *et al.* 2014a	6
2A	0.0	20.0	*Yr17* (2NS -2AS translocation)	Helguera *et al.* 2003	72
2A	1.9	16.8	*QYr.uga-2AS_26R61*	Hao *et al.* 2011	17
2A	2.0	6.3	*Yr56*	Wheat Catalogue	66
2A	2.9	4.0	***QYr.ucw-2A.2 (IWA422)***	This study	
2A	2.1	7.6	*QYr.ufs-2A_Cappelle-Desprez_Yr16*	Agenbag *et al.* 2012	1
2A	2.3	2.5	*QYr.sun-2A_Wollaroi*	Bansal *et al.* 2014	3
2A	2.5	7.6	*QYr.inra_2AS.1_Recital*	Dedryver *et al.* 2009	13
2A	4.0	15.0	*QYrva.vt-2AS_VA00W-38*	Christopher *et al.* 2013	11
2A	8.3	11.9	*QYr.inra-2AL_CampRemy*	Mallard *et al.* 2005	33
2A	9.9	13.1	*QYrst.orr-2AS_Stephens*	Vazquez *et al.* 2012	55
2A	6.7	14.1	*QYr.ucw-2A_PI610750*	Lowe *et al.* 2011	27
2A	26.3	27.4	***QYr.ucw-2A.3 (IWA424)***	This study	
2A	37.9	41.4	*QYr.sun-2AS_Kukri*	Bariana *et al.* 2010	4
2A	33.3	41.4	*Yrxy2*	Zhou *et al.* 2011	63
2A	48.3	59.5	*Yr32*	Eriksen *et al.* 2004	73
2A	75.9	83.2	*Yr1*	Wheat Catalogue	66
2A	79.1	100.0	*QYr.inra_2AL.2_Camp Remy*	Boukhatem *et al.* 2002	7
2B	10.0	15.5	*QYr.inra-2BS_Renan*	Dedryver *et al.* 2009	13
2B	10.6	11.4	*QYrst.orr-2B.1_Stephens*	Vazquez *et al.* 2012	55
2B	18.6	21.3	*QYr-2B-Attila (Yr27)*	Rosewarne *et al.* 2008	47
2B	19.9	30.4	*QYrlu.cau-2BS1_Luke*	Guo *et al.* 2008	16
2B	25.1	27.4	*QYrid.ui-2B.1_IDO444*	Chen *et al.* 2012	67
2B	27.4	30.0	*QYr.sgi-2B.1_Kariega*	Prins *et al.* 2011	40
2B	27.4	29.2	*YrP81*[b]	Wheat Catalogue	66

2B	27.5	29.1	*YrC51*	Zheng *et al.* 2014	62
2B	28.2	32.0	*QYr.sgi-2B.1_Kariega*	Ramburan *et al.* 2004	69
2B	29.6	47.9	*Yr41*	Wheat Catalogue	66
2B	29.1	32.2	*QYr.cim-2BS_(Yr31)_Chapio*	Yang *et al.* 2013	60
2B	29.6	44.5	*QYrlo.wpg-2BS_Louise*	Carter *et al.* 2009	8
2B	30.4	50.2	*QYrid.ui-2B.2_IDO444*	Chen *et al.* 2012	67
2B	30.4	36.4	*QYrst.orr-2BS.2_Stephens*	Vazquez *et al.* 2012	55
2B	30.6	32.3	*QYrlu.cau-2BS2_Luke*	Guo *et al.* 2008	16
2B	32.3	33.5	*YrH9014*	Ma *et al.* 2013	32
2B	32.3	49.1	*QYr.caas-2BS_Pingyuan 50*	Lan *et al.* 2010	70
2B	34.4	36.4	*Yr27*	Wheat Catalogue	66
2B	35.4	44.3	*QYr-2B_Opata 85*	Boukhatem *et al.* 2002	7
2B	36.6	47.9	*QYr.tam-2BL_TAM111*	Basnet *et al.* 2014a	6
2B	30.4	44.3	*YrKK*	Wheat Catalogue	66
2B	39.8	49.1	*QYr.ucw-2B_UC1110*	Lowe *et al.* 2011	27
2B	42.1	50.5	*QYr.inra-2B.1_Camp Remy*	Mallard *et al.* 2005	33
2B	44.3	47.9	*QYr.cim-2BS_Francolin*	Lan *et al.* 2014	21
2B	59.8	60.0	*QYr.inra-2B.2_CampRemy*	Mallard *et al.* 2005	33
2B	60.1	66.6	*QYr.caas-2BL_Naxos*	Ren *et al.* 2012a	45
2B	62.1	73.6	*QYraq.cau-2BL_Aquileja*	Guo *et al.* 2008	16
2B	62.1	64.5	*Yr5*	McGrann *et al.* 2014	34
2B	63.9	66.1	*Yr44*	Xu *et al.* 2013	58
2B	66.1	78.1	*Yr53*	Xu *et al.* 2013	58
2B	78.1	82.1	*Yr43*	Xu *et al.* 2013	58
2B	86.8	90.7	*Yr3*	Wheat Catalogue	66
2B	89.2	90.8	*QYr-2B_Avocet*	Rosewarne *et al.* 2008	47
2D	0.0	4.8	*QYr.caas-2DS_Libellula*	Lu *et al.* 2009	28
2D	34.2	36.9	*QYr.ufs-2DS_ Cappelle-Desprez*	Agenbag *et al.* 2012	1
2D	31.4	55.8	*QYr.inra-2DS_Camp Remy*	Mallard *et al.* 2005	33
2D	44.1	55.8	*QYr.caas-2DL_Naxos*	Ren *et al.* 2012a	45
2D	55.6	61.3	*QYr.jic-2D_Guardian*	Melichar *et al.* 2008	36
2D	68.4	79.5	*QYr.tam_2D_Quaiu*	Basnet *et al.* 2014b	5
2D	72.1	81.5	*QYr.jic-2D_Briagdier*	Jagger *et al.* 2011	20
2D	67.1	74.5	*Yr55*	Wheat Catalogue	66
2D	70.7	80.3	*Yr54*	Wheat Catalogue	66
3A	5.0	9.2	*QYr-1B_Saar*	Lillemo *et al.* 2008	24
3A	12.8	22.4	*QYrst.orr-3AL_Stephens*	Vazquez *et al.* 2012	55
3A	37.4	42.0	*QYr.cau-3AL_AQ24788-53*	Quan *et al.* 2013	42
3A	69.7	75.5	*QYr.cim-3A_Avocet*	Rosewarne *et al.* 2012	48
3B	0.0	6.2	*QYr-3B_Opata85*	Singh *et al.* 2000	50
3B	0.0	1.8	*Yr4*	Wheat Catalogue	66
3B	0.0	10.0	*Yr57*	Wheat Catalogue	66
3B	2.3	6.9	*Yr30*	Suenaga *et al.* 2003	53

3B	0.0	5.3	*QYr-3B.1-Pavon76*	Williams *et al.* 2006	57
3B	0.0	6.7	*QYr.cim-3BS_Chapio_Yr30*	Yang *et al.* 2013	60
3B	2.1	13.7	*QYr.tam-3B_Quaiu*	Basnet *et al.* 2014b	5
3B	2.3	14.9	*QYr.cim-3BS.2_Frankolin*	Lan *et al.* 2014	21
3B	2.3	7.0	*QYr-3B_Oligoculm*	Suenaga *et al.* 2003	53
3B	2.3	5.7	*QYr-3B_Alturas*	Zhao *et al.* 2012	61
3B	2.8	7.9	*QYr.inra-3BS_Renan*	Dedryver *et al.* 2009	13
3B	4.4	6.7	*QYr.ucw-3BS_UC1110*	Lowe *et al.* 2011	27
3B	6.5	8.0	***QYr.ucw-3B.2 (IWA5202)***	This study	
3B	6.7	11.6	*QYr.uga-3BS.1_AGS2000*	Hao *et al.* 2011	17
3B	14.9	16.5	*Yrns-B1*	Wheat Catalogue	66
3B	28.3	37.6	*QYr.sun-3B_Kukri*	Bariana *et al.* 2010	4
3B	42.1	60.7	*QYr.cim-3B_Pastor*	Rosewarne *et al.* 2012	48
3B	50.5	60.9	*QYr.inra-3Bcentr_Renan*	Dedryver *et al.* 2009	13
3B	51.2	65.1	*QYrpi.vt-3BL_VA00W-38*	Christopher *et al.* 2013	11
3B	68.6	71.9	*QYr.sun-3B_Wollaroi*	Bansal *et al.* 2014	3
3B	85.6	98.1	*QYrid.ui-3B_Rio Blanco*	Chen *et al.* 2012	67
3B	88.2	99.5	*QYrex.wgp-3BL_Express*	Lin and Chen 2009	25
3D	0.4	2.4	*Yr66*	Wheat Catalogue	66
3D	7.1	8.6	*Yr49*	Wheat Catalogue	66
3D	4.6	24.1	*QYr.tam-3D_Quaiu*	Basnet *et al.* 2014b	5
3D	60.2	72.0	*Yr45*	Wheat Catalogue	66
4A	70.5	80.8	*Yr51*	Randhawa *et al.* 2014	43
4A	74.2	79.1	*QYr-4A_Sachem*	Singh *et al.* 2013	51
4A	74.2	81.2	*QYr.orr-4AL_Stephens*	Vazquez *et al.* 2012	55
4A	74.4	80.2	*QYr.sgi-4A.2_Kariega*	Ramburan *et al.* 2004	69
4A	76.1	84.5	*QYr.sgi-4A.1_Kariega*	Ramburan *et al.* 2004	69
4A	78.8	85.7	*QYr.ui-4A_IDO444*	Chen *et al.* 2013	67
4A	80.1	94.8	*QYr.sgi-4A.1 and 4A.2_Kariega*	Prins *et al.* 2011	40
4A	83.3	85.7	*Yr60*	Wheat Catalogue	66
4A	84.3	85.8	***QYr.ucw-4A (IWA1034)***	This study	
4B	32.9	40.7	*QYr-4B_Sachem*	Singh *et al.* 2013	51
4B	34.6	51.9	*QYr.ufs-4B_Palmiet*	Agenbag *et al.* 2012	1
4B	36.3	64.0	*QYr.sun-4B_Janz*	Zwart *et al.* 2010	65
4B	39.0	68.6	*Yr50*	Liu *et al.* 2013	26
4B	39.4	51.9	*QYr-4B_Avocet*	William *et al.* 2006	57
4B	49.2	52.4	*QYr.caas-4BL_Libellula*	Lu *et al.* 2009	28
4B	49.7	53.8	*QYr.ui-4B_Rio Blanco*	Chen *et al.* 2014	67
4B	52.1	58.1	*Yr62*	Lu *et al.* 2014	29
4B	54.8	62.1	*QYr.jic-4B_Alcedo*	Jagger *et al.* 2011	20
4B	58.1	60.4	*QYr.vt-4BL_VA00W-38*	Christopher *et al.* 2013	11
4B	58.1	62.1	*QYr.jic-4B_Guardian*	Melichar *et al.* 2008	35
4B	65.8	73.3	*QYr-4B_Oligoculm*	Suenaga *et al.* 2003	53

4D	14.0	15.9	*YrAS2388*	Wheat Catalogue	66
4D	18.4	34.0	*QYr.cim-4DS_Pastor*	Rosewarne *et al.* 2012	48
4D	9.4	11.4	*Yr28*	Wheat Catalogue	66
4D	49.5	50.8	*Yr46/Lr67*	Herrera-Foessel *et al.* 2011	18
4D	45.4	53.7	*QYr.caas-4DL_Bainong64*	Ren *et al.* 2012b	44
4D	47.2	49.1	***QYr.ucw-4D (IWA5375)***	This study	
4D	54.0	62.3	*QYr-4D_Oligoculm*	Suenaga *et al.* 2003	53
5A	22.3	23.9	*QYr.cau-5AS_AQ24788-53*	Quan *et al.* 2013	42
5A	35.7	42.3	*QYr.cim-5AL_Francolin*	Lan *et al.* 2014	21
5A	66.8	79.2	*QYr-5A_Opata85*	Boukhatem *et al.* 2002	7
5A	66.8	79.9	*QYr.cim-5AL_Pastor*	Rosewarne *et al.* 2012	48
5A	81.2	84.6	*QYr.caas-5AL.2_SHA3/CBRD*	Ren *et al.* 2012a	45
5A	81.5	88.3	*QYr.caas-5AL_Pingyuan 50*	Lan *et al.* 2010	70
5A	86.8	100	*Yr48*	Wheat Catalogue	66
5A	84.5	100.0	*QYr.ucw-5AL_PI610750*	Lowe *et al.* 2011	27
5A	86.7	88.1	***QYr.ucw-5A.1 (IWA6988)***	This study	
5A	89.9	100.0	*Yr34*	Wheat Catalogue	66
5B	3.3	10.0	*Yr47*	Wheat Catalogue	66
5B	11.4	16.7	*QYr.uga-5B_AGS2000*	Hao *et al.* 2011	17
5B	20.8	37.0	*QYr.cim-5BL_Chapio*	Yang *et al.* 2013	60
5B	37.0	41.2	*QYr.ufs-5B_Cappelle-Desprez*	Agenbag *et al.* 2012	1
5B	37.5	38.9	*QYr.tem-5B.1_Flinor*	Feng *et al.* 2011	14
5B	42.1	46.5	*QYr.inra-5B.1_CampRemy*	Mallard *et al.* 2005	33
5B	53.8	55.2	*YrExp2*	Wheat Catalogue	66
5B	45.2	47.6	*QYr.caas-5BL.1_Libellula*	Lu *et al.* 2009	28
5B	47.6	52.3	*QYr-5B_Oligoculm*	Suenaga *et al.* 2003	53
5B	63.3	72.9	*QYr.sun-5B_Janz*	Bariana *et al.* 2010	4
5B	65.4	67.4	*QYr.caas-5BL.3_SHA3/CBRD*	Ren *et al.* 2012a	45
5B	70.3	75.7	*QYr.tem-5B.2_Flinor*	Feng *et al.* 2011	14
5B	70.3	73.2	*QYr.caas-5BL.2_Libellula*	Lu *et al.* 2009	28
5B	70.3	74.8	*QYr.inra-5BL.2_CampRemy*	Mallard *et al.* 2005	33
5B	84.4	97.1	*QYr.sun-5B_Wollaroi*	Bansal *et al.* 2014	3
5B	84.8	100.0	*QYr.ui-5B_IDO444*	Chen *et al.* 2012	67
5D	0.0	4.0	*Yr40*	Wheat Catalogue	66
6A	0.0	7.1	*QYr.uga-6AS_26R61*	Hao *et al.* 2011	17
6A	3.8	7.1	*QYr.wgp-6AS_Express*	Lin and Chen 2009	25
6A	7.6	17.8	*QYr.cim-6A_Avocet*	Rosewarne *et al.* 2012	48
6A	49.7	68.0	*QYr.cim-6AL_Francolin*	Lan *et al.* 2014	21
6A	56.9	67.3	*YrLM168*	Feng *et al.* 2014	15
6A	61.6	66.8	*QYr.ufs-6A_Kariega*	Prins *et al.* 2011	40
6A	62.7	74.6	*QYr-6A_Saar*	Lillemo *et al.* 2008	24
6A	63.9	73.1	*QYr-6A_Avocet*	Williams *et al.* 2006	57
6A	71.3	74.7	*QYr.orr-6AL_Stephens*	Vazquez *et al.* 2012	55

6B	6.9	12.3	*QYr.ufs-6B_Kariega*	Prins *et al.* 2011	40
6B	9.4	19.7	*QYr.tam-6BS_TAM111*	Basnet *et al.* 2014a	6
6B	10.3	16.4	*QYr.caas-6BS.2_Naxos*	Ren *et al.* 2012a	45
6B	0	14.9	*Yr35*	Wheat Catalogue	66
6B	12.6	16.4	*QYr.caas-6BS_Bainong64*	Ren *et al.* 2012b	44
6B	15.9	21.4	*QYr.wgp-6BS.2_Stephens*	Santra *et al.* 2008	49
6B	17.1	34.8	*QYr.sun-6B_Janz*	Bariana *et al.* 2010	4
6B	23.1	28.6	*QYr-6B_Oligoculm*	Suenaga *et al.* 2003	53
6B	23.1	25.1	*Yr36*	Uauy *et al.* 2005	54
6B	24.1	34.8	*QYr.wgp-6B.1_Stephens*	Santra *et al.* 2008	49
6B	27.4	42.3	*QYr.caas-6BS_Pingyuan 50*	Lan *et al.* 2010	70
6B	42.3	50.2	*QYr-6B_Pavon76*	Williams *et al.* 2006	57
6B	42.3	50.2	*QYr.inra-6B_Renan*	Dedryver *et al.* 2009	13
6B	56.7	58.3	***QYr.ucw-6B (IWA7257)***	This study	
6B	70.0	79.9	*QYr.cim-6BL_Pastor*	Rosewarne *et al.* 2012	48
6D	42.4	56.1	*QYr.ufs-6D_Cappelle-Desprez*	Agenbag *et al.* 2012	1
6D	47.9	49.8	***QYr.ucw-6D (IWA167)***	This study	
6D	71.6	77.8	*QYr-6D_W-7984*	Boukhatem *et al.* 2002	7
7A	0.0	6.1	*QYr.cim-7AS_Avocet*	Rosewarne *et al.* 2012	48
7A	6.5	10.1	*QYr.inra-7A_Recital*	Dedryver *et al.* 2009	13
7A	12.5	22.8	*QYr.caas-7A_Jingshuan16*	Ren *et al.* 2012b	44
7A	12.5	40.2	*QYr.sun-7A_CPI133872*	Zwart *et al.* 2010	65
7A	19.5	32.4	*Yr61*	Wheat Catalogue	66
7A	42.0	49.3	*Yrxy1*	Zhou *et al.* 2011	63
7A	69.9	77.1	*QYr.orr-7A_Stephens*	Vazquez *et al.* 2012	55
7A	74.4	85.5	*QYr.cim-7BL_Avocet*	Rosewarne *et al.* 2012	48
7A	74.6	100.0	*QYr.sgi-7A_Kariega*	Prins *et al.* 2011	40
7B	0.0	1.0	*Yr63*	Wheat Catalogue	66
7B	21.0	27.7	*QYr-7B_Oligoculm*	Suenaga *et al.* 2003	53
7B	27.5	48.1	*Yr39*	Lin and Chen 2007	71
7B	33.8	56.3	*QYr.caas-7B.1_SHA3/CBRD*	Ren *et al.* 2012a	45
7B	41.6	47.8	*QHtap.wsu-7BL_Alpowa*	Lin and Chen 2007	71
7B	41.8	45.9	*QYr.orr-7BS_Stephens*	Vazquez *et al.* 2012	55
7B	43.8	58.7	*QYr.sun-7B_Kukri*	Bariana *et al.* 2010	4
7B	68.8	75.2	*QYr-7B_Tiritea*	Imtiaz *et al.* 2004	19
7B	71.8	73.6	*QYr.caas-7BL.2_SHA3/CBRD*	Ren *et al.* 2012a	45
7B	73.5	87.6	*QYr.cim-7BL_Pastor*	Rosewarne *et al.* 2012	48
7B	82.7	87.8	*YrC591*	Wheat Catalogue	66
7B	80.4	82.7	*YrZH84*	Wheat Catalogue	66
7B	80.5	86.2	*QYr-7BL_Strongfield*	Singh *et al.* 2013	51
7B	75.5	77.5	*Yr67*	Wheat Catalogue	66
7B	81.6	83.2	*Yr52*	Wheat Catalogue	66
7B	82.6	88.4	*QYr-7B_Attila*	Rosewarne *et al.* 2008	47

7B	80.9	84.7	*Yr59*	Wheat Catalogue	66
7D	26.1	33.6	*Yr18/Lr34* (*csLV23*)	Yang *et al.* 2013	60
7D	49.8	60.2	*Yr33*	Wheat Catalogue	66

[a] "Wheat Catalogue" here represents both the Catalogue of Gene Symbols for Wheat (McIntosh *et al.* 2013) and the 2013-2014 Supplement (http://wheat.pw.usda.gov/GG2/Triticum/wgc/2013/2013-2014_Supplement.pdf).

[b] *YrP81* is completely linked to *YrC51* but there was not space to place in **Figure 6**.

COMPARISON OF QTL IDENTIFIED IN THIS STUDY WITH PREVIOUSLY MAPPED *PST* RESISTANCE GENES AND QTL

For this comparison the 10 highly-significant QTL identified in this study and previously mapped *Pst* resistance genes and QTL were both projected in the same integrated map in **File S4**. Distances expressed as % of total length were calculated by dividing the cM position by the total length of the respective map.

QYr.ucw-1B = IWA3892 (1BL)

IWA3892 was mapped on the long arm of chromosome 1B at position 153.1 cM (CI= 87.9 - 89.7%, **File S4**), 19 cM from the most distal marker available for this chromosome (172.4 cM= 100%). This GWAS study detected a second SNP marker (*IWA2077*) associated with *Pst* resistance In the distal region of the 1BL arm. *IWA2077* was mapped 17.8 cM distal to *IWA3892* (CI= 98.9 - 100%). Although *IWA2077* was not significant experiment-wise, it showed significant marker-wise associations for the three locations tested in this study (**Table S4** and **Figure S2**). These results suggest that there might be more than one *Pst* resistance gene in this region, a hypothesis also supported by the presence of non-overlapping QTL (**Figure S6**). For example, the CI of *IWA3892* overlaps with *QYr.sun-1B* (Zwart *et al.* 2010), whereas the CI of *IWA2077* corresponds better with *QYr.cim-1BL* (Rosewarne *et al.* 2012; Lan *et al.* 2014). This last QTL was attributed to the *Yr29/Lr46* locus, which confers partial adult plant resistance to both leaf rust and stripe rust (Bariana *et al.* 2001, 2010; William *et al.* 2003, 2006; Rosewarne *et al.* 2006, 2008, 2012; Lillemo *et al.* 2008; Melichar *et al.* 2008; Zwart *et al.* 2010; Jagger *et al.* 2011; Lan *et al.* 2014). Allelism studies will be required to determine the relationship among the different sources of *Pst* resistance mapped to the distal region of chromosome arm 1BL and the two QTL identified in this study.

QYr.ucw-1D = IWA980 (1DS)

IWA980 was mapped on the centromeric region of the short arm of chromosome 1D, at position 49.3 cM (24.7%, **File S4**). The three QTL for *Pst* resistance previoulsy reported on chromosome 1D (Zwart *et al.* 2010; Vazquez *et al.* 2012; Ren *et al.* 2012a) were mapped on the distal region of chromosme arm 1DS (10% of 1D arm length). Therefore, QTL IWA980 is most likely a novel *Pst* resistance QTL.

QYr.ucw-2A.2 = IWA422 (2AS)

IWA422 was mapped on the distal region of the short arm of chromosome 2A at position 9.9 cM (3.4%, **File S4**). The short arm of chromosome 2A includes *Pst* resistance genes *Yr56* (Catalogue of Gene Symbols for Wheat, Supplement 2013-2014) and *Yr17* (Helguera *et al.* 2003). *Yr56* was mapped between SSR markers *barc124* and *gwm512* (3.2% of 2A) in a very similar position as

IWA422. Yr17 is located within a large distal translocation (25–38 cM= 20% of 2A) of chromosome 2NS from *T. ventricosum* into wheat chromosome 2AS (Helguera *et al.* 2003), which does not recombine with the wheat chromosomes. *IWA422* is likely different from *Yr17* because most new *Pst* races in the western USA are virulent on *Yr17*, whereas the QTL associated with *IWA422* was effective in all three locations tested in this study. At least nine QTL have been identified in the distal region of chromosome arm 2AS (Mallard *et al.* 2005; Dedryver *et al.* 2009; Lowe *et al.* 2011; Hao *et al.* 2011; Vazquez *et al.* 2012; Agenbag *et al.* 2012; Christopher *et al.* 2013; Basnet *et al.* 2014a; Bansal *et al.* 2014). *IW422*, *Yr56*, and the previous nine QTL have been all mapped within the distal 17% of chromosome 2A, which will complicate the separation of the different resistance loci using allelism tests. The colinear regions for IWA422 in *Brachypodium* and rice include multiple NB-LRR and LRR-receptor-like kinases suggesting that this region may include an ancestral R gene cluster.

QYr.ucw-2A.3 = IWA424 (2AS)

IWA424 was mapped on the short arm of chromosome arm 2A at position 78.3 cM (26.9%, **File S4**). This relative chromosome position is proximal to *IWA422* and the nine linked QTL described above, whose confidence intervals do not overlap with *IWA424* (**Figure 6**). Resistance gene *Yrxy2* (Zhou *et al.* 2011) and *QYr.sun-2AS* (Bariana *et al.* 2010) both map between 38% and 41% of chromosome 2A (long arm centromeric region) with no overlap with *QYr.ucw-2A.3* QTL (26.5-27.4%). These results suggest that *IWA424* may be a novel *Pst* resistance locus.

QYr.ucw-3B.2 = IWA5202 (3BS)

IWA5202 was mapped on the short arm of chromosome 3B at position 15.4 cM (7.2%, **File S4**). Four different named *Pst* resistance genes have been mapped on the short arm of chromosome 3B. The most distal one is *Yr4* (=*YrRub*, Bansal *et al.* 2010), which is 5 cM distal to *Yr30* based on common marker *barc75* (Singh *et al.* 2000; Suenaga *et al.* 2003) and 4 cM distal to *Yr57* based on allelism tests (Catalogue of gene Symbols for Wheat: Supplement 2013-2104). The last two genes were mapped roughly between 3.4 and 7% of the chromosome length, and partially overlap with this QTL (5.6-7.6%), suggesting that it may be the effect of the same resistance gene. Finally, *Yrns-B1* (Khlestkina *et al.* 2007) maps between 14.9% and 16.5% of the arm length, outside the CI of this QTL. In addition to the named *Yr* genes, the distal region of chromosome arm 3BS (15% of the 3B length) includes ten additional QTL (**Table S11**). One of these QTL, *QYr.ucw*-3B.1 (Lowe *et al.* 2011), is no longer effective against the new *Pst* races present in California and is likely different from *QYr.ucw*-3B.2 reported in this study. The *Brachypodium* and rice regions colinear to wheat *IWA5202* include several NB-LRR and LRR-receptor-like kinases suggesting that this region may include an ancestral R gene cluster.

QYr.ucw-4A = IWA1034 (4AL)

IWA1034 was mapped on the long arm of chromosome 4 at position 181.8 cM (85%, **File S4**) in the region translocated from chromosome 7BS. *IWA1034* was mapped within the confidence interval of *QYr.sgi-4A.1/4A.2* (80.1% to 94.8%, Prins *et al.* 2011) and may represent the same gene. *IWA2170* (167.3 cM =78.3%) was mapped proximal to *IWA1034* and was significantly associated with *Pst* resistance in all three locations tested in this study (BLUE DVS $P<0.001$, MTV $P<0.01$ and PLM $P<0.05$) but not in the experiment-wise Bonferroni test. *IWA2170* overlaps with *QYr-4A* (Singh *et al.* 2013) and *QYr.orr-4A* (Vazquez *et al.* 2012) and may represent the same locus.

QYr.ucw-4D = IWA5375 (4DL)

IWA5375 was mapped on the long arm of chromosome 4D at position 82 cM (48.1%, **File S4**). This position overlaps with previously mapped *Yr46/Lr67* gene complex (Hiebert *et al.* 2010; Herrera-Foessel *et al.* 2011) and *QYr.caas-4DL* (Ren *et al.* 2012a) suggesting that they may represent the same locus. Additional indirect evidence supports this hypothesis: The resistant allele for *IWA5375* occurs at high frequency only in South Asia (subpopulations 4C frequency = 0.88) and *Yr46* was transferred from a *T. aestivum* accession from Pakistan (PI 250413). The introgressed segment from PI 250413 was linked to SSR loci *cfd71*, *barc98*, *cfd23*, and *wmc457* (Hiebert *et al.* 2010), which defines a region from 36.7% to 49.6% in our integrated map that overlaps with *IWA5475*. Finally, marker *csSNP856* (=*Kasp856*), which was tightly linked to *Lr67/Yr46* (Forrest *et al.* 2014) is in linkage disequilibrium with *IWA5375* (r^2 value = 0.41, **File S3**).

QYr.ucw-5A.1 = IWA6988 (5AL)

IWA6988 was mapped on the distal region of chromosome arm 5AL (195.8 cM, 87.4%, **File S4**), close to previously mapped partial resistance genes *Yr48* (Lowe *et al.* 2011) and *Yr34* (Bariana *et al.* 2006). However, the interpretation of the relationship between *IWA6988* and these two *Yr* genes is complicated by the existence of a linked SNP in this region that is also associated with *Pst* resistance in the GWAS. Marker *IWA2646* is 5.6 cM distal to *IWA6988* (**FileS4**) and showed significant associations with *Pst* resistance in five out of the six environments tested in this study (P<0.01 three locations and P<0.001 two locations, **Table S4** and **Figure S2**). However, *IWA6988* and *IWA2646* are not in LD (r^2 = 0.04) suggesting that they may be associated to different resistance genes. A high-density map developed in our laboratory showed that *Yr48* is more closely linked to *IWA2646* than to *IWA6988.* This result was further supported by the presence of deletions for *IWA2646* but not for *IWA6988* in susceptible radiation mutants of *Yr48* (Hegarty and Dubcovsky personal communication). Based on these results, *IWA2646* seems to be closer to *Yr48* than *IWA6988. IWA6988* may be associated to a different resistance gene. *Pst* resistance genes *Yr34* has been also mapped on the distal region of chromosome arm 5AL (projected at 98.3%, **Table S11**), but the relationship with *Yr48* is not known because there are no close molecular markers linked to *Yr34* (Bariana *et al.* 2006). The determination of the precise relationship between *Yr34, Yr48* and the two significant SNP detected in our GWAS (*IWA6988* and *IWA2646*) will require further allelism tests.

QYr.ucw-6B = IWA7257 (6BL)

IWA7257 was mapped on the long arm of chromosome 6B at position 112.3 cM (57.5%, **File S4**). Three studies identified *Pst* resistance QTL in the proximity of this QTL but their confidenc intervals do not overlap with the confidence interval of *QYr.ucw-6B* (56.5 - 58.5%). *QYr-6B-Pavon76* (Williams et al. 2006) and *QYr.inra-6B* (Dedryver *et al.* 2009) CI extends between 42% and 50%, whereas *QYr.cim-6BL* was mapped between 70% and 80% of the chromosome length (**File S4**). Therefore, *QYr.ucw-6B* confidence interval seems to be different from the closest published QTL and may represent a novel *Pst* resistance locus.

QYr.ucw-6D = IWA167 (6DS)

IWA167 was mapped on the short arm of chromosome 6D at position 82.2 cM (48.8%, **File S4**). This position overlaps with the confidence interval of *QYr.ufs-6D* (Agenbag *et al.* 2012), which was projected onto the integrated map (**File S4**) between 42.4%

and 56% (**Table S11**). However, the peak of *QYr.ufs-6D* was mapped on the long arm of chromosome 6D (Agenbag *et al.* 2012), whereas *IWA167* is located on the short arm of chromosome 6D. The strength of the QTL also seems to be different. *QYr.ufs-6D* was associated with a small effect on *Pst* resistance and was not consistent across years, whereas *QYr.ucw-6D* was the most significant QTL in our study and showed highly significant effects in every environment (**Figure S2**). Finally, the *IWA167* allele associated with *Pst* resistance is present at high frequency only in South Asia (**Table S6**), making unlikely its presence in Cappelle-Desprez, the donor of the resistance allele at *QYr.ufs-6D*. Taken together, these observations suggest that these two QTL represent the effect of different genes. More detailed comparative maps will be required to test this hypothesis.

LITERATURE CITED

Agenbag, G. M., Z. A. Pretorius, L. A. Boyd, C. M. Bender, and R. Prins, 2012 Identification of adult plant resistance to stripe rust in the wheat cultivar Cappelle-Desprez. Theor. Appl. Genet. 125: 109–120.

Bansal, U. K., M. J. Hayden, M. B. Gill, and H. S. Bariana, 2010 Chromosomal location of an uncharacterised stripe rust resistance gene in wheat. Euphytica 171: 121–127.

Bansal, U. K., A. G. Kazi, B. Singh, R. A. Hare, and H. S. Bariana, 2014 Mapping of durable stripe rust resistance in a durum wheat cultivar Wollaroi. Mol. Breed. 33: 51–59.

Bariana, H. S., U. K. Bansal, A. Schmidt, A. Lehmensiek, J. Kaur *et al.*, 2010 Molecular mapping of adult plant stripe rust resistance in wheat and identification of pyramided QTL genotypes. Euphytica 176: 251–260.

Bariana, H. S., M. J. Hayden, N. U. Ahmed, J. A. Bell, P. J. Sharp *et al.*, 2001 Mapping of durable adult plant and seedling resistances to stripe rust and stem rust diseases in wheat. Aust. J. Agric. Res. 52: 1247–1255.

Bariana, H. S., N. Parry, I. R. Barclay, R. Loughman, R. J. McLean *et al.*, 2006 Identification and characterization of stripe rust resistance gene *Yr34* in common wheat. Theor. Appl. Genet. 112: 1143–1148.

Basnet, B. R., A. M. H. Ibrahim, X. Chen, R. P. Singh, E. R. Mason *et al.*, 2014a Molecular mapping of stripe rust resistance in hard red winter wheat TAM 111 adapted to the U.S. high plains. Crop Sci. 54: 1361-1373.

Basnet, B. R., R. P. Singh, A. M. H. Ibrahim, S. A. Herrera-Foessel, J. Huerta-Espino *et al.*, 2014b Characterization of *Yr54* and other genes associated with adult plant resistance to yellow rust and leaf rust in common wheat Quaiu 3. Mol. Breed. 33: 385–399.

Boukhatem, N., P. V. Baret, D. Mingeot, and J. M. Jacquemin, 2002 Quantitative trait loci for resistance against yellow rust in two wheat-derived recombinant inbred line populations. Theor. Appl. Genet. 104: 111–118.

Carter, A. H., X. M. Chen, K. Garland-Campbell, and K. K. Kidwell, 2009 Identifying QTL for high-temperature adult-plant resistance to stripe rust (*Puccinia striiformis* f. sp. *tritici*) in the spring wheat (*Triticum aestivum* L.) cultivar “Louise.” Theor. Appl. Genet. 119: 1119–1128.

Chen, J., C. Chu, E. J. Souza, M. J. Guttieri, X. Chen *et al.*, 2012 Genome-wide identification of QTL conferring high-temperature adult-plant (HTAP) resistance to stripe rust (*Puccinia striiformis* f. sp. *tritici*) in wheat. Mol. Breed. 29: 791–800.

Cheng, P., L. S. Xu, M. N. Wang, D. R. See, and X. M. Chen, 2014 Molecular mapping of genes *Yr64* and *Yr65* for stripe rust resistance in hexaploid derivatives of durum wheat accessions PI 331260 and PI 480016. Theor. Appl. Genet. 127: 2267–2277.

Christopher, M. D., S. Liu, M. D. Hall, D. S. Marshall, M. O. Fountain *et al.*, 2013 Identification and mapping of adult plant stripe rust resistance in soft red winter wheat VA00W-38. Crop Sci. 53: 871-879.

Dedryver, F., S. Paillard, S. Mallard, O. Robert, M. Trottet *et al.*, 2009 Characterization of genetic components involved in durable resistance to stripe rust in the bread wheat "Renan". Phytopathology 99: 968–973.

Eriksen, L., F. Afshari, M. J. Christiansen, R. A. McIntosh, A. Jahoor *et al.*, 2004 *Yr32* for resistance to stripe (yellow) rust present in the wheat cultivar Carstens V. Theor. Appl. Genet. 108: 567–575.

Feng, J., G. Chen, Y. Wei, Y. Liu, Q. Jiang *et al.*, 2014 Identification and genetic mapping of a recessive gene for resistance to stripe rust in wheat line LM168-1. Mol. Breed. 33: 601–609.

Feng, J., L. L. Zuo, Z. Y. Zhang, R. M. Lin, Y. Y. Cao *et al.*, 2011 Quantitative trait loci for temperature-sensitive resistance to *Puccinia striiformis* f. sp. *tritici* in wheat cultivar Flinor. Euphytica 178: 321–329.

Forrest, K., V. Pujol, P. Bulli, M. Pumphrey, C. Wellings *et al.*, 2014 Development of a SNP marker assay for the *Lr67* gene of wheat using a genotyping by sequencing approach. Mol. Breed. 34: 2109–2118.

Guo, Q., Z. J. Zhang, Y. B. Xu, G. H. Li, J. Feng *et al.*, 2008 Quantitative trait loci for high-temperature adult-plant and slow-rusting resistance to *Puccinia striiformis* f. sp. *tritici* in wheat cultivars. Phytopathology 98: 803–809.

Hao, Y., Z. Chen, Y. Wang, D. Bland, J. Buck *et al.*, 2011 Characterization of a major QTL for adult plant resistance to stripe rust in US soft red winter wheat. Theor. Appl. Genet. 123: 1401–1411.

Helguera, M., I. A. Khan, J. Kolmer, D. Lijavetzky, L. Zhong-qi *et al.*, 2003 PCR assays for the cluster of rust resistance genes and their use to develop isogenic hard red spring wheat lines. Crop Sci. 43: 1839–1847.

Herrera-Foessel, S. A., E. S. Lagudah, J. Huerta-Espino, M. J. Hayden, H. S. Bariana *et al.*, 2011 New slow-rusting leaf rust and stripe rust resistance genes *Lr67* and *Yr46* in wheat are pleiotropic or closely linked. Theor. Appl. Genet. 122: 239–249.

Hiebert, C. W., J. B. Thomas, B. D. McCallum, D. G. Humphreys, R. M. DePauw *et al.*, 2010 An introgression on wheat chromosome 4DL in RL6077 (Thatcher*6/PI 250413) confers adult plant resistance to stripe rust and leaf rust (Lr67). Theor. Appl. Genet. 121: 1083–1091.

Imtiaz, M., M. Ahmad, M. G. Cromey, W. B. Griffin, and J. G. Hampton, 2004 Detection of molecular markers linked to the durable adult plant stripe rust resistance gene *Yr18* in bread wheat (*Triticum aestivum* L.). Plant Breed. 123: 401–404.

Jagger, L. J., C. Newell, S. T. Berry, R. MacCormack, and L. A. Boyd, 2011 The genetic characterisation of stripe rust resistance in the German wheat cultivar Alcedo. Theor. Appl. Genet. 122: 723–733.

Khlestkina, E. K., M. S. Röder, O. Unger, A. Meinel, and A. Börner, 2007 More precise map position and origin of a durable non-specific adult plant disease resistance against stripe rust (*Puccinia striiformis*) in wheat. Euphytica 153: 1–10.

Lan, C., S. Liang, X. Zhou, G. Zhou, Q. Lu *et al.*, 2010 Identification of genomic regions controlling adult-plant stripe rust resistance in chinese landrace pingyuan 50 through bulked segregant analysis. Phytopathology 100: 313–318.

Lan, C., G. M. Rosewarne, R. P. Singh, S. A. Herrera-Foessel, J. Huerta-Espino *et al.*, 2014 QTL characterization of resistance to leaf rust and stripe rust in the spring wheat line Francolin#1. Mol. Breed. 34: 789–803.

Lillemo, M., B. Asalf, R. P. Singh, J. Huerta-Espino, X. M. Chen *et al.*, 2008 The adult plant rust resistance loci *Lr34/Yr18* and *Lr46/Yr29* are important determinants of partial resistance to powdery mildew in bread wheat line Saar. Theor. Appl. Genet. 116: 1155–1166.

Lin, F., and X. M. Chen, 2007 Genetics and molecular mapping of genes for race-specific all-stage resistance and non-race-specific high-temperature adult-plant resistance to stripe rust in spring wheat cultivar Alpowa. Theor. Appl. Genet. 114: 1277–1287.

Lin, F., and X. M. Chen, 2009 Quantitative trait loci for non-race-specific, high-temperature adult-plant resistance to stripe rust in wheat cultivar Express. Theor. Appl. Genet. 118: 631–642.

Liu, J., Z. Chang, X. Zhang, Z. Yang, X. Li *et al.*, 2013 Putative *Thinopyrum intermedium*-derived stripe rust resistance gene *Yr50* maps on wheat chromosome arm 4BL. Theor. Appl. Genet. 126: 265–274.

Lowe, I., D. Cantu, and J. Dubcovsky, 2011 Durable resistance to the wheat rusts: integrating systems biology and traditional phenotype-based research methods to guide the deployment of resistance genes. Euphytica 179: 69–79.

Lu, Y., C. Lan, S. Liang, X. Zhou, D. Liu *et al.*, 2009 QTL mapping for adult-plant resistance to stripe rust in Italian common wheat cultivars Libellula and Strampelli. Theor. Appl. Genet. 119: 1349–1359.

Lu, Y., M. Wang, X. Chen, D. See, S. Chao *et al.*, 2014 Mapping of *Yr62* and a small-effect QTL for high-temperature adult-plant resistance to stripe rust in spring wheat PI 192252. Theor. Appl. Genet. 127: 1449–1459.

Lukaszewski, A. J., 2000 Manipulation of the 1RS.1BL translocation in wheat by induced homoeologous recombination. Crop Sci. 40: 216-225.

Ma, D., D. Y. Hou, M. Tang, H. Wang, Q. Li *et al.*, 2013 Genetic analysis and molecular mapping of a stripe rust resistance gene YrH9014 in wheat line H9014-14-4-6-1. J. Integr. Agric. 12: 638–645.

Ma, J., R. Zhou, Y. Dong, L. Wang, X. Wang *et al.*, 2001 Molecular mapping and detection of the yellow rust resistance gene *Yr26* in wheat transferred from *Triticum turgidum* L. using microsatellite markers. Euphytica 120: 219–226.

Mallard, S., D. Gaudet, A. Aldeia, C. Abelard, A. L. Besnard *et al.*, 2005 Genetic analysis of durable resistance to yellow rust in bread wheat. Theor. Appl. Genet. 110: 1401–1409.

McGrann, G. R., P. H. Smith, C. Burt, G. R. Mateos, T. N. Chama *et al.*, 2014 Genomic and genetic analysis of the wheat race-specific yellow rust resistance gene *Yr5*. J. Plant Sci. Mol. Breed. 3: http://dx.doi.org/10.7243/2050-2389-3-2.

McIntosh, R. A., Y. Yamazaki, J. Dubcovsky, W. J. Rogers, C. F. Morris *et al.*, 2013 Catalogue of gene symbols for wheat in *12th Int. Wheat Genet. Symp.*, edited by R.A. McIntosh, Yokohama, Japan

Melichar, J. P. E., S. Berry, C. Newell, R. MacCormack, and L. A. Boyd, 2008 QTL identification and microphenotype characterisation of the developmentally regulated yellow rust resistance in the UK wheat cultivar Guardian. Theor. Appl. Genet. 117: 391–399.

Prins, R., Z. A. Pretorius, C. M. Bender, and A. Lehmensiek, 2011 QTL mapping of stripe, leaf and stem rust resistance genes in a Kariega × Avocet S doubled haploid wheat population. Mol. Breed. 27: 259–270.

Quan, W., G. Hou, J. Chen, Z. Du, F. Lin *et al.*, 2013 Mapping of QTL lengthening the latent period of *Puccinia striiformis* in winter wheat at the tillering growth stage. Eur. J. Plant Pathol. 136: 715–727.

Ramburan, V. P., Z. A. Pretorius, J. H. Louw, L. A. Boyd, P. H. Smith *et al.*, 2004 A genetic analysis of adult plant resistance to stripe rust in the wheat cultivar Kariega. Theor. Appl. Genet. 108: 1426–1433.

Randhawa, M., U. Bansal, M. Valárik, B. Klocová, J. Doležel *et al.*, 2014 Molecular mapping of stripe rust resistance gene *Yr51* in chromosome 4AL of wheat. Theor. Appl. Genet. 127: 317–324.

Ren, Y., Z. He, J. Li, M. Lillemo, L. Wu *et al.*, 2012a QTL mapping of adult-plant resistance to stripe rust in a population derived from common wheat cultivars Naxos and Shanghai 3/Catbird. Theor. Appl. Genet. 125: 1211–1221.

Ren, Y., Z. Li, Z. He, L. Wu, B. Bai *et al.*, 2012b QTL mapping of adult-plant resistances to stripe rust and leaf rust in Chinese wheat cultivar Bainong 64. Theor. Appl. Genet. 125: 1253–1262.

Rosewarne, G. M., R. P. Singh, J. Huerta-Espino, S. A. Herrera-Foessel, K. L. Forrest *et al.*, 2012 Analysis of leaf and stripe rust severities reveals pathotype changes and multiple minor QTLs associated with resistance in an Avocet × Pastor wheat population. Theor. Appl. Genet. 124: 1283–1294.

Rosewarne, G. M., R. P. Singh, J. Huerta-Espino, and G. J. Rebetzke, 2008 Quantitative trait loci for slow-rusting resistance in wheat to leaf rust and stripe rust identified with multi-environment analysis. Theor. Appl. Genet. 116: 1027–1034.

Rosewarne, G. M., R. P. Singh, J. Huerta-Espino, H. M. William, S. Bouchet *et al.*, 2006 Leaf tip necrosis, molecular markers and β1-proteasome subunits associated with the slow rusting resistance genes *Lr46/Yr29*. Theor. Appl. Genet. 112: 500–508.

Santra, D. K., X. M. Chen, M. Santra, K. G. Campbell, and K. K. Kidwell, 2008 Identification and mapping QTL for high-temperature adult-plant resistance to stripe rust in winter wheat (*Triticum aestivum* L.) cultivar "Stephens." Theor. Appl. Genet. 117: 793–802.

Singh, R., M. Matus-Cádiz, M. Båga, P. Hucl, and R. N. Chibbar, 2010 Identification of genomic regions associated with seed dormancy in white-grained wheat. Euphytica 174: 391–408.

Singh, R. P., J. C. Nelson, and M. E. Sorrells, 2000 Mapping *Yr28* and other genes for resistance to stripe rust in wheat. Crop Sci. 40: 1148–1155.

Singh, A., M. P. Pandey, A. K. Singh, R. E. Knox, K. Ammar *et al.*, 2013 Identification and mapping of leaf, stem and stripe rust resistance quantitative trait loci and their interactions in durum wheat. Mol. Breed. 31: 405–418.

Suenaga, K., R. P. Singh, J. Huerta-Espino, and H. M. William, 2003 Microsatellite markers for genes *Lr34/Yr18* and other quantitative trait loci for leaf rust and stripe rust resistance in bread wheat. Phytopathology 93: 881–890.

Uauy, C., J. C. Brevis, X. Chen, I. Khan, L. Jackson *et al.*, 2005 High-temperature adult-plant (HTAP) stripe rust resistance gene *Yr36* from *Triticum turgidum* ssp. *dicoccoides* is closely linked to the grain protein content locus *Gpc-B1*. Theor. Appl. Genet. 112: 97–105.

Vazquez, M. D., C. J. Peterson, O. Riera-Lizarazu, X. Chen, A. Heesacker *et al.*, 2012 Genetic analysis of adult plant, quantitative resistance to stripe rust in wheat cultivar "Stephens" in multi-environment trials. Theor. Appl. Genet. 124: 1–11.

William, M., R. P. Singh, J. Huerta-Espino, S. O. Islas, and D. Hoisington, 2003 Molecular marker mapping of leaf rust resistance gene lr46 and its association with stripe rust resistance gene *Yr29* in wheat. Phytopathology 93: 153–159.

William, H. M., R. P. Singh, J. Huerta-Espino, G. Palacios, and K. Suenaga, 2006 Characterization of genetic loci conferring adult plant resistance to leaf rust and stripe rust in spring wheat. Genome 49: 977–990.

Xu, L. S., M. N. Wang, P. Cheng, Z. S. Kang, S. H. Hulbert *et al.*, 2013 Molecular mapping of *Yr53*, a new gene for stripe rust resistance in durum wheat accession PI 480148 and its transfer to common wheat. Theor. Appl. Genet. 126: 523–533.

Yang, E.-N., G. M. Rosewarne, S. A. Herrera-Foessel, J. Huerta-Espino, Z.-X. Tang *et al.*, 2013 QTL analysis of the spring wheat “Chapio” identifies stable stripe rust resistance despite inter-continental genotype × environment interactions. Theor. Appl. Genet. 126: 1721–1732.

Zhao, L., J. Feng, C.-Y. Zhang, X.-D. Xu, X.-M. Chen *et al.*, 2012 The dissection and SSR mapping of a high-temperature adult-plant stripe rust resistance gene in American spring wheat cultivar Alturas. Eur. J. Plant Pathol. 134: 281–288.

Zheng, J., Z. Yan, L. Zhao, S. Li, Z. Zhang *et al.*, 2014 Molecular mapping of a stripe rust resistance gene in wheat line C51. J. Genet. 93: 443–450.

Zhou, X. L., W. L. Wang, L. L. Wang, D. Y. Hou, J. X. Jing *et al.*, 2011 Genetics and molecular mapping of genes for high-temperature resistance to stripe rust in wheat cultivar Xiaoyan 54. Theor. Appl. Genet. 123: 431–438.

Zwart, R. S., J. P. Thompson, A. W. Milgate, U. K. Bansal, P. M. Williamson *et al.*, 2010 QTL mapping of multiple foliar disease and root-lesion nematode resistances in wheat. Mol. Breed. 26: 107–124.
